# The role of expertise and culture in visual art appreciation

**DOI:** 10.1038/s41598-022-14128-7

**Published:** 2022-06-23

**Authors:** Kohinoor M. Darda, Emily S. Cross

**Affiliations:** 1 Institute of Neuroscience and Psychology, University of Glasgow, Glasgow, UK; 2 Department of Cognitive Science, Macquarie University, Sydney, Australia; 3 Penn Center for Neuroaesthetics, University of Pennsylvania, Philadelphia, PA USA; 4 MARCS Institute for Brain, Behaviour and Development, Western Sydney University, Sydney, Australia

**Keywords:** Psychology, Human behaviour

## Abstract

Is art appreciation universal? Previous evidence suggests a general preference for representational art over abstract art, and a tendency to like art originating from one’s own culture more than another culture (an ingroup bias), modulated by art expertise. However, claims about universality are difficult given that most research has focused on Western populations. Across two pre-registered and statistically powered experiments, we explore the role of culture and art expertise in the aesthetic evaluation of Indian and Western paintings and dance depicting both abstract and representational content, by inviting expert and art-naïve Indian and Western participants to rate stimuli on beauty and liking. Results suggest an ingroup bias (for dance) and a preference for representational art (for paintings) exists, both modulated by art expertise. As predicted, the ingroup bias was present only in art-naïve participants, and the preference for representational art was lower in art experts, but this modulation was present only in Western participants. The current findings have two main implications: (1) they inform and constrain understanding of universality of aesthetic appreciation, cautioning against generalising models of empirical aesthetics to non-western populations and across art forms, (2) they highlight the importance of art experience as a medium to counter prejudices.

## Introduction

Across millennia, humans have expressed themselves through the medium of art. Art is often considered to be a society’s collective memory, preserving what fact-based historical records may not be able to—how it felt to exist in a particular time and in a particular space. More recently, the function of art to communicate and soothe, and to bring people together has been emphatically highlighted during the COVID-19 pandemic. Whether it is people singing to each other from their balconies^1^, musicians performing orchestral works over Zoom^2^, or artists tapping into their creativity to explain public health guidelines^3^, art has given hope and provided much-needed human connection, across geographic, racial, and cultural boundaries. In a world facing increasing societal stresses due to racism, political polarisation, xenophobia, and other geostrategic fractures, art proves its ability to bring people together.

In the words of Danish-Icelandic contemporary artist Olafur Eliasson, art “… helps us identify with one another and expands our notion of we—from the local to the global”^4^. Engagement with art is not always a solitary event. Instead, it represents one of the few areas in society where people can come together to share experiences even when they have radically different beliefs or worldviews. But to what extent can art really bind us together, and can it transcend boundaries of culture or country? Do we share much more than what divides us? Or do our in-group biases and preferences persist when watching dance performances or viewing paintings?

The degree to which aesthetic preferences are universal or shared across cultures, as opposed to being highly individual in nature and moderated by our in-group biases is an important question in empirical aesthetics. To date, however, little insight has been gained into the universality of psychological underpinnings of aesthetic appreciation, as most research has exclusively examined perceptions and preferences among Western European and North American populations^5^. Moreover, influence of the visual properties of the artwork (such as its content), as well as observers’ attributes (such as their culture or art expertise) on aesthetic preferences have been extensively studied for the fine arts (including paintings, drawings and sculpture), while our knowledge of such attributes for other artforms remains limited (e.g.,^6–8^). This is somewhat surprising, given the ubiquity and importance of a range of artforms beyond the fine arts, including music, theatre, poetry, and dance, across many cultures. The current work attempts to begin to bridge several of these considerable gaps in knowledge regarding the human aesthetic experience by one, evaluating whether universal primitives underpin people’s appreciation in fine and performing arts; and two, examining the extent to which cultural background shapes these preferences. To accomplish this, we have combined paintings and dance choreography under a common analytical framework, with exemplars from “western” (Anglo-European) and “eastern” (Indian) artistic practices. Addressing the universality of aesthetic preference, and its modulation by expertise and culture in paintings and dance should lead to a better understanding and a fresh and culturally inclusive reconceptualization of long-debated issues in empirical aesthetics such as the nature of aesthetic judgements and evaluations, and how a beholder’s attributes shape their aesthetic experience.

Theoretical accounts of aesthetic processing have proposed the influence of cultural contexts, as well as the differing meaning of beauty across cultures (e.g.,^9^). There is also some evidence to suggest cross-species universal aesthetic appreciation and perception of visual patterns such as symmetry^10^, and the universality of musical aesthetic processing (e.g.,^11^). However, our focus in the current empirical work is on cross-cultural differences (or similarities) in the visual (fine and performing) arts, specifically paintings and dance, and therefore we focus the following review of extant literature primarily on empirical cross-cultural investigations of paintings and dance.

### The universality of the preference for representational art and its modulation by expertise

The creation and appreciation of art finds a place in all cultures, serving different social, religious, economic, and political functions^12,13^. If engagement with art is indeed universal, as has been argued, then it seems plausible that the processes underpinning aesthetic appreciation are also shared across cultures. Indeed, evidence suggests that people from different cultures base their aesthetic appreciation on a common set of features such as symmetry, contrast, colour, brightness, complexity, and proportion (for a review, see^5^). Nevertheless, on face value, nothing seems more subjective than the human appreciation of art. People differ in their aesthetic preferences—some may like contemporary art, while others have intense negative feelings toward it^14^. Previous work also demonstrates how individual differences such as art expertise, understanding, and knowledge, as well as personality traits influence aesthetic evaluations^15–18^.

Aesthetic judgements and ratings of visual art have also been found to be highly idiosyncratic depending on the content depicted in the art^6,19^, or the contextual framing with which artworks are introduced^20^. Across a range of studies, a number of research teams report that paintings and images with representational content, i.e. those depicting landscapes, people, still life scenes, and so on, are preferred and assigned higher ratings of aesthetic qualities such as beauty and liking, compared to paintings or images with abstract content that do not represent anything concrete or figurative^6,8,17,21–23^. Preferences for representational content are also more reliable and consistent compared to abstract content. For example, Schepman and colleagues show greater agreement across people for representational compared to abstract paintings and images, and the semantic associations generated by viewers for these artworks are also more convergent across individuals for representational art compared to abstract art^6,23^. One proposed explanation for this preference and agreement across viewers for representational content focuses on the meaningfulness of the depiction: people may prefer art that they find meaningful, and semantic associations may be better shared for meaningful stimuli compared to abstract ones^21^.

The meaning drawn from the content of an artwork also depends on the observer’s experience, expertise, and knowledge of the artworks. Indeed, previous evidence has shown increased aesthetic ratings for abstract artworks among those with expertise and knowledge of art^24–26^. Therefore, although a general preference for representational artworks compared to abstract artworks seems to exist, evidence also suggests that art expertise modulates this preference such that the preference for representational (compared to abstract) artworks among art experts is more attenuated than that reported among art-naïve participants. However, the universality of this preference for visual art can be contested on the grounds that all previous research in this domain has focused exclusively on static paintings or images, and the evaluations of participants from western cultures (primarily Western Europe and North America).

It is important to note that representational and abstract artworks are not restricted to the fine arts. Dance forms across many cultures also have both representational and abstract content, such as dance that involves movements, tropes, or symbols to depict certain social or cultural themes and characters (representational dance), and dance that is purely for an aesthetic but non-symbolic, non-representative purpose (abstract dance; ^27–29^). Characteristics of a dance piece, such as its complexity, acceleration, predictability, uniformity, difficulty or reproducibility, movement amplitude, and evocativeness have been evidenced to predict the aesthetic ratings of dance^30–32^. A growing body of research has focused on the characteristics of the observer or spectator such as their visual and motor expertise with dance, and familiarity and competency with the dance movement vocabulary and how this affects aesthetic ratings^33–37^. Similar to paintings, the aesthetic ratings for dance are also higher for dance experts, and are modulated by similar features such as evocativeness, familiarity, and complexity. Yet, the representativeness of the content of dance and its modulation by dance expertise has to date received little attention in dance, even though, like paintings, dance can be representative or abstract^30^.

Therefore, in the current study, to address our first research question, we use mixed effects models to test whether preferences for representational art shows evidence for cultural as well as artform universality, and is modulated in a similar manner by expertise across participants from Indian and Western cultures across the domains of paintings and dance. If a universal preference for representational visual art generalises across art forms (paintings and dance), we would expect to find a preference for representational paintings and dance over abstract paintings and dance, modulated by art/dance expertise such that this preference is attenuated among painting/dance experts compared to painting/dance naïve participants. In addition, a universal preference for representational art should also emerge across cultures, and be modulated by expertise in a similar way across participants belonging to different cultures.

It is possible that a preference for representational art may be because of the familiarity or complexity of a representational artwork compared with an abstract artwork, as opposed to the abstractness of the content itself. To isolate the abstractness of the content of the paintings, we also investigated whether the universal preference for representational art and its modulation by expertise would further persist above and beyond subjective ratings of complexity, familiarity, evocativeness, reproducibility, and technical competency that have been demonstrated to influence aesthetic ratings and preferences for paintings and dance.

### The universality of the ingroup bias for visual arts and its modulation by expertise

Art across the world can differ in its subject matter, production methods, the role(s) played by the artist and the spectator, and its categorisation into different art forms and styles. Cultural differences can perhaps also explain why some artworks are thought to be beautiful to some spectators and not to others. Artists from diverse cultures often report distinct aesthetic experiences when looking at the same visual art displays, and use varied geometric and metaphorical perspectives to represent the visual world in their artworks, employing specific ways to depict spatial and temporal information^7,38,39^. For instance, Western representational paintings can be very precise reproductions of the world at that time point, whereas in Indian and Chinese paintings, several periods of time can appear on the artwork at the same time^7^. Similarly, popular forms of Indian classical dance feature religious symbolism and depictions with a strong spiritual connection, whereas well-known western classical dance forms, such as ballet, do not^40^.

Previous studies investigating cultural differences in aesthetic appreciation of visual art have mainly focused on the visual processing of scenes and objects, and cultural similarities and differences in the processing of formal features such as colour perception and curvature^39,41–43^. In the domain of dance, research on cultural differences in dance appreciation is extremely sparse. One study to date has reported that Indian participants who had more visual experience or visual familiarity with Indian dance (Bharatanatyam) showed enhanced cortico-spinal excitability when viewing Bharatanatyam videos compared to Western dance (ballet) videos, whereas Western participants who have more visual experience with ballet showed higher cortico-spinal excitability when viewing ballet videos, suggesting enhanced motor resonance when watching a movement style that is more familiar^44^. However, the extent to which and how aesthetic preferences for dance might differ across cultures remains unstudied.

The few studies that have looked at the influence of cultural differences on aesthetic evaluations of artwork suggest that individuals show a preference for artworks that belong to their own culture, or correspond to their cultural traditions, compared to artworks that belong to another culture^7,43^. Specifically, Yang et al.^43^ found that Western participants showed higher valence values when viewing Western paintings, but Chinese participants did not show this effect. Bao et al.^7^ showed a double dissociation such that Chinese participants rated Chinese paintings higher on beauty compared to Western paintings, and Western participants rated Western paintings higher on beauty ratings compared to Chinese paintings. One explanation put forth for this double dissociation is a simple in-group bias^45^. Group biases (typically in-group favouritism and out-group dislike) are prevalent in day-to-day interactions wherein individuals show in-group favouritism for members of their own race, culture, ethnicity, and sex (e.g.,^46–50^). In a similar manner, individuals looking at artworks from their own culture may feel a sense of cultural identity and belongingness, and therefore rate it higher on aesthetic ratings compared to artworks from other cultures^7^. This preference can be implicit, i.e., when individuals are not explicitly aware that the painting belongs to their own cultural background. In contrast, the preference may only exist or be heightened when individuals have explicit knowledge of cultural closeness and can identify the painting as belonging to their own cultures.

The feeling of cultural identity, however, may not be uniform across participants with different levels of art experience, sensitivity, expertise, or knowledge. While previous research has not directly investigated the modulation of ingroup bias by the expertise of the spectator, some evidence suggests that people who are interested in art agree on their aesthetic judgements irrespective of their individual cultural backgrounds^51,52^. These studies, however, systematically manipulate only either the cultural background of the participants or artworks belonging to different cultures, but not both in the same experiment. Therefore, an intriguing open question remains whether a sense of cultural identity is higher in art naïve participants who may show a higher ingroup bias compared to experts who may show an attenuated ingroup bias compared to non-experts.

Therefore, to address our second research question, we use mixed effects models to test the extent to which an ingroup bias exists for both cultures such that Western participants prefer Western paintings and dance, and Indian participants prefer Indian paintings and Indian dance, compared to paintings and dance belonging to the other culture. We predict that art/dance expertise should modulate this ingroup bias similarly for both Indian and Western participants, such that experts should show no (or a reduced) preference compared to non-experts for paintings or dance belonging to their own culture compared to another culture. This ingroup bias and its modulation by expertise should further persist above and beyond the subjective ratings of complexity, familiarity, evocativeness, reproducibility, and technical competency that have been known to have an influence on aesthetic ratings and preferences of paintings and dance.

## Methods

### Open science statement

Across all experiments, we report how the sample size was determined, all data exclusions, and all measures used in the study^53,54^. For both experiments, data pre-processing, statistical analyses, and data visualisations were performed using R (R Core Team, 2018), unless otherwise specified. Following open science initiatives^55^, all raw data are available online for other researchers to pursue alternative questions of interest, along with analysis scripts and stimuli used (https://osf.io/vtw54/). Data analyses for both experiments were preregistered on AsPredicted.org (Experiment 1: https://aspredicted.org/65Y_W6X, Experiment 2: https://aspredicted.org/VBB_T2F). The datasets generated and/or analysed during the current study are available in the Open Science Framework (OSF) repository [https://osf.io/vtw54/].

For both Experiments 1 and 2, mixed effects model analyses were executed using the ordinal package (v.2019.12–10) in R v.1.3.1093. (R Core Team). Post-hoc tests were executed using the emmeans package (v.1.5.1). We used an alpha of 0.05 to make inferences, and controlled for multiple comparisons using Tukey-HSD in post-hoc tests. Model fit was compared using the anova() function (Chi-square test).

### Experiment 1—Paintings

#### Sample size justification

We determined the sample size based on a simulation-based power analysis approach using the simr R package^56^. First, we used pilot data (N = 22, 14 females, 10 art experts, Mean_age_ = 29.71, SD_age_ = 9.86) for beta weight estimation for the following linear mixed effects model: beauty ~ category*expertise + (1|subject) + (1|item). Second, we simulated data by extending along the sample size, i.e., as a function of different sample sizes. Our main focus was the interaction between the category of the painting and the art expertise of participants, and the power analysis suggested that we required a sample size of 50 participants (25 experts and 25 non-experts) with 35 items to have > 80% power to detect a significant category*expertise interaction (more details on the power analyses and the code can be found on OSF and in the supplementary material, see Figure S1). We therefore aimed to stop data collection when over 100 participants finished the entire survey, with an aim to recruit approximately 50 Indian participants and 50 Western participants with 25 experts and 25 non-experts within each culture.

#### Participants

Participants were recruited using the online data collection tool PsyToolkit^57,58^. Participants were primarily recruited from India and UK/Europe and classified into either Indian or Western culture participants (see Supplementary Table 2 for a geographic distribution of the sample) by advertisement on social media. All participants provided informed consent, and had normal or corrected-to-normal vision. Ethical approval was obtained from the University of Glasgow ethics review board (300190209), and all experiments were performed in accordance with the Declaration of Helsinki. Participants were reimbursed with an Amazon gift card of either 6 GBP or Rs. 550 INR.

A total of 145 participants started the online experiment, with 113 participants completing the full experiment. Participants were excluded if they did not pass our attention check questions (see section “Tasks and Procedures”; N = 19), or did not provide required demographic information (age, gender, and culture; N = 2). The final sample consisted of 92 participants (17 males, 75 females; Mean_age_ = 25.52, SD_age_ = 3.96) which included 45 Indian participants (21 experts, 24 non-experts) and 47 Western participants (21 experts, 26 non-experts). All participants provided informed consent, and had normal or corrected-to-normal vision (a geographic distribution of the participant sample is provided in Table S2). 

#### Stimuli

**Figure 1:**
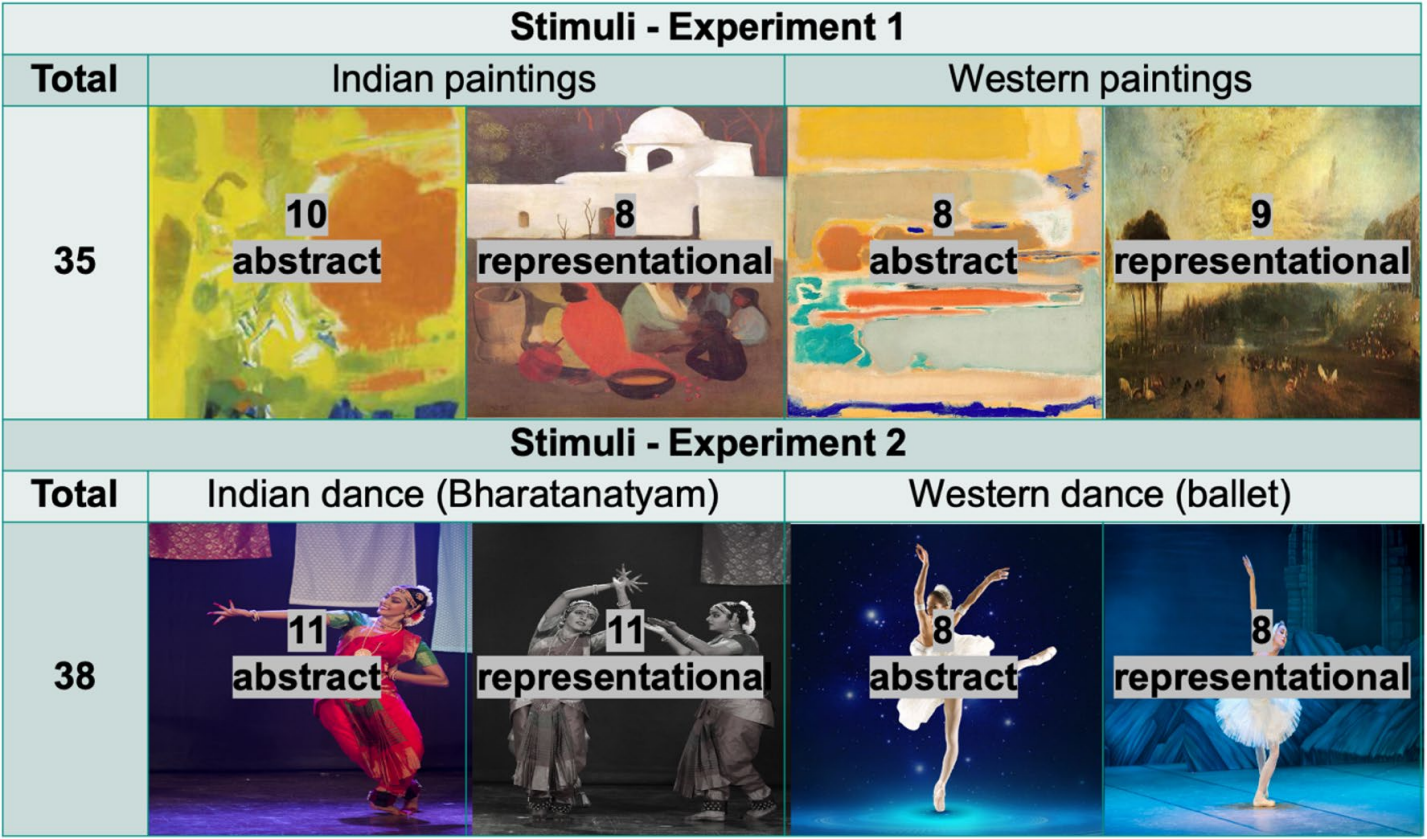
Categories of paintings and dance (abstract/representational) across different sources of painting or dance style (Indian/Western). Note: All images used in Figs. 1 and 2 are in the public domain, and/or we have informed consent from individuals for publication of their image in an online open access publication. Images used to depict abstract and representational paintings and dance in Figure 1 are not images of the actual stimuli used. Stimuli used in the current study are available online on the OSF.

An independent sample of art naïve participants (N = 21, 13 females, Mean_age_ = 29.14, SD_age_ = 6.73) rated the first pool of images for abstract and representational paintings on familiarity, complexity, and evocativeness, and categorized them into ‘abstract’ or ‘representational’. A total of 120 paintings were selected and resized to 500 × 500 pixels: 60 by Indian painters, 60 by Western painters, out of which 30 each were abstract and representational paintings. Paintings were categorized by the experimenters as either ‘abstract’ or ‘representational’ depending on the content of the painting. That is, paintings depicting representational or figurative content (such as still life or landscapes) were categorized as ‘representational’ and paintings depicting content that was abstract (or not representative of anything concrete or figurative) were classified as ‘abstract.’ Participants also categorized the paintings into whether they thought the painting was abstract or representational. The final stimulus set consisted of paintings with ratings most similar to each other on the three variables of familiarity, complexity, and evocativeness. These paintings were also accurately classified as either ‘abstract’ or ‘representational’ i.e. the categorization by the participants matched the categorization made by the experimenters (more details along with the analysis code can be found here: https://osf.io/vtw54/). The final stimulus set consisted of 35 paintings—10 Indian abstract, 8 Western abstract, 8 Indian representational, and 9 Western representational paintings (see Fig. 1; note that the number of stimuli is unbalanced across categories because of the type of analysis we used to balance paintings on the variables of familiarity, complexity, and evocativeness. See the Supplementary material for more information). The paintings were resized to 500 × 500 pixels, and matched for mean luminance using the SHINE toolbox in MATLAB^59^. Therefore, the final stimulus set was closely matched across all four categories of paintings (Indian Abstract, Indian Representational, Western Abstract, Western Representational) on variables of luminance, familiarity, complexity, and evocativeness (for mean ratings for each painting category, please refer to Table S1).

#### Tasks and procedure

**Figure 2:**
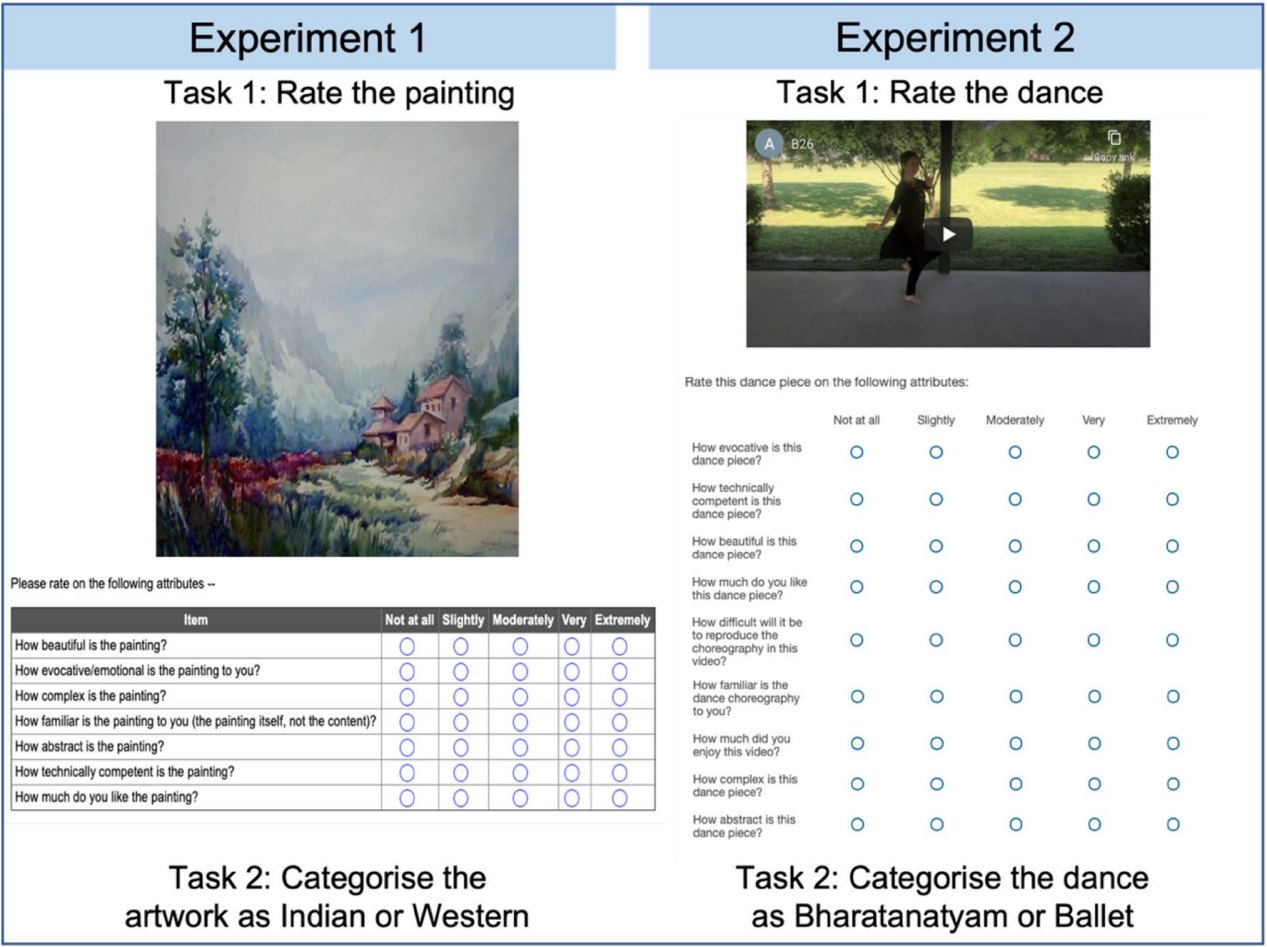
Graphical representation of the rating task and categorisation task for Experiments 1 and 2.

Participants completed two tasks—a rating task and a categorization task (see Fig. 2). In the rating task, participants saw a painting on the screen, and were asked to rate it on a 5-point likert scale from low (1) to high (5) with ‘1’ corresponding to ‘not at all’, ‘2’ corresponding to ‘slightly’, ‘3’ corresponding to ‘moderately’, ‘4’ corresponding to ‘very’, and ‘5’ corresponding to ‘extremely’ on the following variables:

1. Familiarity (how familiar is the painting?)
2. Complexity (how complex is the painting?)
3. Evocativeness (how evocative or emotional is the painting?)
4. Abstractness (how abstract is the painting?)
5. Technical competency (how technically competent is the painting or the painter who made the painting?)
6. Beauty (how beautiful do you find the painting?)
7. Liking (how much do you like the painting?)

The order in which these questions were presented was randomized for each item, and the order in which the items (35 paintings) were presented was also randomized across participants. Two additional questions appeared randomly during the rating task which served as attention check questions: “how attentive are you while doing this experiment?” and “how honest are you while doing this experiment?” The 5-point likert scale remained the same as for the other variables. Participants who responded < 4 on the 5-point likert scale were excluded from the analyses.

In the categorisation task, participants categorized the same 35 paintings they saw during the rating task into either ‘Indian’ or ‘Western’ depending on whether they thought the painting was produced by a painter of Indian origin or a painter of Western origin. The order of the items (35 paintings) was randomized across participants. Participants also completed two questionnaires—the Art Experience Questionnaire^60^ to gauge their art expertise, and (unrelated to the current study) the Vienna Art Interest and Art Knowledge (VAIAK) questionnaire^61^. They were also asked if they were art professionals or not—participants were categorized as ‘non-experts’ if they were art naïve or had no experience or qualifications related to the arts, and participants were categorized as art ‘experts’ if they worked as art professionals or had completed their Masters in an arts-related field (fine arts, or arts history; detailed data of arts expertise in participants based on academic and professional qualifications, as well as their scores on the Art Experience Questionnaire, can be found on OSF). The experiment started with some demographic questions, the Art Experience Questionnaire, and then participants completed the rating task, the categorisation task, and the VAIAK questionnaire. The order of tasks and questionnaires remained constant across participants, and the tasks were self-paced. The entire experiment did not last for more than 60 min for most participants (Mean_timetaken_ = 50.86 min, SD_timetaken_ = 39.25). The script used for online experiment presentation in Psytoolkit is provided on the OSF.

#### Data analysis

We recorded ratings for each item for each participant on all variables for the rating task. For the categorisation task, we recorded which items were classified as either Indian or Western by participants (source of painting as rated by participants). We also calculated accuracy by calculating the percentage of items that were correctly categorized as Indian or Western i.e. when the actual source of the painting matched the participant’s response.

RQ1: Do expertise and culture influence aesthetic judgements of representational and abstract art (preregistered and confirmatory)?

Beauty and liking ratings were analysed separately. The current analyses differ from our pre-registered analyses in three ways:

1. Our study was powered to detect a category*expertise interaction with N = 50. We aimed to collect N = 50 for both cultures, and include the category*expertise*culture interaction as a fixed effect in the current model. We were able to collect N = 45 Indian and N = 47 Western participants i.e. a total N of 92 participants. Therefore, while we are powered to detect the category*expertise interaction in the total sample, we are not sufficiently powered to detect the three-way interaction of culture*expertise*category. Therefore, any findings we report are suggestive and exploratory, and not confirmatory.
2. We pre-registered a linear mixed effects analysis using the ‘lme4’ package in R^62^. However, because the data were ordinal in nature, we decided to analyse the ordinal data using cumulative link mixed models by using the ‘ordinal’ package in R^63^. Analysing the data using ‘lme4’ yielded similar results.
3. In the preregistered analyses, we included category (abstract, representational) and art expertise (expert, nonexpert) as categorical fixed effects of interest, and the by-subject and by-item intercept as a random factor for the model. However, given recommendations for the “keep it maximal” approach to multilevel modeling^64^, we further included the maximal number of random effects that the design permitted.

The categorical variables were coded using a deviation coding style where factors sum to zero and the intercept can then be interpreted as the grand mean and the main effects can be interpreted similarly to a conventional ANOVA^65^. As such, the categorical variables of category, expertise, culture, and source of painting were coded as 0.5 (representational/expert/Indian/Indian) and − 0.5 (abstract/nonexpert/Western/Western). An ordinal logistic regression was employed in the form of a cumulative-link mixed model (ordinal package, “clmm” function; 63) using logit (log-odds) as link, and flexible thresholds between the ordinal scores. We chose this approach because the dependent or outcome variables ‘beauty’ and ‘liking’ ratings were ordinal in nature (ratings on a Likert scale 1–5). The model thus measures the probability of specific ratings being above certain thresholds without the assumption that the thresholds are symmetric or equidistant from each other. In order to address our first question of interest i.e. whether representational and abstract art judgements are modulated by art expertise, and whether this is similar for both cultures, we included the three way interaction of category (abstract, representational), expertise (expert, nonexpert), and culture (Indian, Western) as a fixed effect in the model. For random effects, we included the maximal number of random effects that the design permitted. The complexity of the random structure was reduced if the results showed failure in model convergence or a singular fit. The final model used was –

$${\text{clmm(beauty}}/{\text{liking}} \sim 1 + {\text{category}}*{\text{expertise}}*{\text{culture}} + (1 + {\text{category}}|{\text{subject}}) + (1 + {\text{ culture}}*{\text{expertise}}|{\text{item}}),{\mathrm{link}} = {\text{``}}{\mathrm{logit}}{\text{''}},{\text{threshold}} = {\text{``}}{\mathrm{flexible}}{\text{''}})$$

To test whether category, expertise, and culture modulated beauty and liking ratings above and beyond the subjective factors that participants rated the paintings on (familiarity, complexity, evocativeness, technical competency), we further added the subjective variables as fixed effects to the model.

$${\text{clmm}}({\text{beauty}}/{\text{liking}} \sim {\text{category}}*{\text{expertise}}*{\text{culture}} + {\text{familiarity}} + {\text{evocativeness}} + {\text{complexity}} + {\text{technical competency}} + (1 + {\text{category}}|{\text{subject}}) + (1 + {\text{culture}}*{\text{expertise}}|{\text{item}}),{\text{link}} = {\text{``}}{\mathrm{logit}}{\text{''}},{\text{threshold}} = {\text{``}}{\mathrm{flexible}}{\text{''}})$$

RQ2: Does expertise shape the ingroup bias for aesthetic judgements (preregistered but exploratory)?

We preregistered the hypotheses for our second research question, but we note that the present study was not powered to detect a three-way interaction of culture, source of painting, and expertise. Therefore, any conclusions we draw from these analyses are exploratory and suggestive, and not confirmatory.

In order to investigate the ingroup bias in aesthetic judgements, we tested for an interaction between culture of participants and the source of painting, and how this was modulated by expertise. Beauty and liking ratings were analysed separately. The categorical variables were coded using a deviation coding style. As such, expertise, culture, and source of painting were coded as 0.5 (Indian/expert/Indian) and − 0.5 (Western/nonexpert/Western). In order to address our second question of interest i.e. whether the ingroup bias exists, and is modulated by art expertise, we included the three way interaction of source of painting (Indian, Western), expertise (expert, nonexpert), and culture (Indian, Western) as a fixed effect in the model. For random effects, we included the maximal number of random effects that the design permitted. The complexity of the random structure was reduced if the results showed failure in model convergence or a singular fit. The final model used was –

$${\text{clmm}}({\text{beauty}}/{\text{liking}} \sim {\text{source}}\_{\mathrm{of}}\_{\mathrm{painting}}*{\text{culture}}*{\text{expertise}} + (1 + {\text{source}}\_{\mathrm{of}}\_{\mathrm{painting}}|{\text{subject}}) + (1 + {\text{culture}}*{\text{expertise}}|{\text{item}}),{\text{link}} = {\text{``}}{\mathrm{logit}}{\text{''}},{\text{threshold}} = {\text{``}}{\mathrm{flexible}}{\text{''}})$$

To test whether source of painting, expertise, and culture modulated beauty and liking ratings above and beyond the subjective factors that participants rated the paintings on (familiarity, complexity, evocativeness, technical competency), we further added the subjective variables as fixed effects to the model.

$${\text{clmm(beauty}}/{\text{liking}} \sim {\text{source}}\_{\mathrm{of}}\_{\mathrm{painting}}*{\text{culture}}*{\text{expertise}} + {\text{familiarity}} + {\text{evocativeness}} + {\text{complexity}} + {\text{technical competency}} + (1 + {\text{source}}\_{\mathrm{of}}\_{\mathrm{painting}}|{\text{subject}}) + (1 + {\text{culture}}*{\text{expertise}}|{\text{item}}),{\text{link}} = {\text{``}}{\mathrm{logit}}{\text{''}},{\text{threshold}} = {\text{``}}{\mathrm{flexible}}{\text{''}}{)}$$

In the analyses above, the factor ‘source of painting’ was coded according to whether a painting was actually painted by an ‘Indian’ artist or a ‘Western’ artist. However, participants were not explicitly made aware while doing the rating task that the paintings were Indian or Western. If an ingroup bias does exist in this case, the preference for paintings from their own cultural background might be reported irrespective of whether participants can accurately identify the painting as Indian or Western in an explicit sense. In contrast, preferences for artworks from one’s own culture may only arise or may be heightened when participants themselves classify the painting as ‘Indian’ or ‘Western’ and have an explicit knowledge of cultural closeness, irrespective of whether or not the paintings were actually made by an ‘Indian’ painter or ‘Western’ painter. In order to test this, we repeated the mixed effects model analyses with the factor ‘source of painting—ppt’ coded as ‘Indian’ or ‘Western’ as categorized by the participants in the categorisation task.

### Experiment 2—Dance

#### Sample size justification

We determined the sample size based on a simulation-based power analysis approach using the simr R package^56^. First, we used pilot data (N = 21, 17 females, 12 dance experts, Mean_age_ = 29.71, SD_age_ = 9.86) for beta weight estimation for the following model: beauty ~ category*expertise + (1|subject) + (1|item). Second, we simulated data by extending along the sample size and plotted statistical power as a function of different sample sizes (see Figure S9; more details on the power analyses and the code can be found here: https://osf.io/vtw54/). Our main focus was the interaction between the category of the dance and the dance expertise of participants, and the power analysis suggested that we required a sample size of 50 participants (25 experts and 25 non-experts) with 38 items to have 80% power to detect a significant category*expertise interaction. We therefore aimed to recruit 50 Indian participants and 50 Western participants with approximately 25 experts and 25 non-experts in each culture.

#### Participants

Participants completed the experiment on Qualtrics. Participants were primarily recruited from India and UK/Europe and classified as either from Indian or Western culture (see the supplementary table S10 for a geographic distribution of the sample). All participants provided informed consent, and had normal or corrected-to-normal vision. Ethical approval was obtained from the University of Glasgow ethics review board (300190209), and all experiments were performed in accordance with the Declaration of Helsinki. Participants were reimbursed with an Amazon gift card of either 6 GBP or Rs. 550 INR.

A total of 161 participants started the online experiment, with 110 participants completing the full experiment. Participants were excluded if they did not pass our attention check questions (see “section Tasks and procedure”; N = 16), and did not provide required demographic information (age, gender, and culture; N = 2). Two participants were further excluded as they did not fit in either the ‘Indian’ or ‘Western’ culture group of participants (a geographic description of our participant sample is provided in the Table S10). The final sample consisted of 90 participants (79 females, 8 males, 3 non-binary; Mean_age_ = 25.94, SD_age_ = 7.51) which included 48 Indian participants (23 experts, 25 non-experts) and 42 Western participants (22 experts, 20 non-experts). All participants provided informed consent, and had normal or corrected-to-normal vision.

#### Stimuli

We invited a professional dancer trained in classical ballet and Bharatanatyam, Sophia Salingaros^66^, to record Bharatanatyam and ballet dance videos. We recorded both ballet and Bharatanatyam dance videos featuring movement sequences that either were intended to represent something in the external world (e.g. humans, animals, birds, nature, etc.) or not represent anything in particular (pure dance, referred to as *nrtta* in Bharatanatyam, or non-representational abstract dance). The videos were edited in iMovie into 10–12 s clips, and the first 0.5 s of the video faded in, and the last 0.5 s faded out to a black screen. An independent sample of participants (all non-dancers, N = 13, 8 females, Mean_age_ = 28.85, SD_age_ = 8.93) rated the first pool of stimuli of abstract and representational dance videos on familiarity, complexity, and evocativeness. Out of a total of 91 videos (46 Bharatanatyam/Indian dance videos, out of which 18 were abstract, and 45 ballet/Western dance videos, out of which 25 were abstract), the final stimulus set was selected by selecting dance videos with ratings most similar to each other on the three variables of familiarity, complexity, and evocativeness (more details along with the analysis code can be found on the OSF). The final stimuli consisted of 38 dance videos—11 Indian abstract, 8 Western abstract, 11 Indian representational, and 8 Western representational dances, matched on the variables of familiarity, complexity, and evocativeness (see Supplementary Material for more details). Mean ratings for each video as rated in the pilot study are reported in Table S9, and all videos are available on the OSF.

#### Tasks and procedure

Participants completed two tasks—a Rating task and a Categorization task (see Fig. 2). In the rating task, participants saw a dance video on the screen, and were asked to rate it on a 5 point likert scale similar to Experiment 1 from low (1) to high (5) on the following variables:

1. Familiarity (how familiar is the dance?)
2. Complexity (how complex is the dance?)
3. Evocativeness (how evocative or emotional is the dance?)
4. Abstractness (how abstract is the dance?)
5. Technical competency (how technically competent is the dance?)
6. Reproducibility (how reproducible is the dance?)
7. Beauty (how beautiful do you find the dance?)
8. Liking (how much do you like the dance?)
9. Enjoyability (how much did you enjoy the dance?)

The order in which these questions were presented was randomized for each item, and the order in which the items (38 dance videos) were presented was also randomized across participants. Two additional attention check questions were also presented randomly during the task (same as Experiment 1) and participants who responded < 4 on the likert scale on these questions were excluded from the analyses.

In the categorisation task, participants categorized the same 38 dance videos they saw during the rating task into either ‘Bharatanatyam’ or ‘Ballet’ depending on whether they thought the dance was of Indian origin or Western origin (the instructions given to the participants were whether they thought the dance was ballet i.e., western classical dance/ of Western origin, or Bharatanatyam i.e., Indian classical dance/ of Indian origin). The order of the items (38 dance videos) was randomized across participants. Participants also completed a dance experience questionnaire similar to the Art Experience Questionnaire (^60^, see the supplementary material for the exact set of questions used to measure dance experience). Participants were asked if they were dance professionals or not—participants were categorized as ‘non-experts’ if they were dance naïve or had no experience with or qualifications related to dance, and participants were categorized as dance ‘experts’ if they worked as dance professionals or had more than 8 years of training in either ballet or Bharatanatyam (a detailed distribution of dance expertise in participants and their scores on the dance experience questionnaire can be found in the supplementary material). The experiment started with some demographic questions, followed by the rating task and the categorisation task. The tasks and questionnaires were self-paced, and the order remained constant across participants. The experiment was self-paced but lasted ~ 120 min for most participants (Mean_time_ = 120.19 min, SD_time_ = 242.40). Results were similar when participants who were 3SD away from the mean time taken by participants to finish the experiment were excluded from the analyses.

#### Data analysis

The data analysis pipeline was the same as Experiment 1 with the following changes: 1) instead of categorizing paintings as Indian and Western, participants categorized dance as either Ballet or Bharatanatyam (labelled as ‘dance style’ instead of ‘source of painting’; 2) we added two additional variables: enjoyability as a dependent variable, and reproducibility as a subjective variable. Therefore, the model with subjective variables for Experiment 2 includes reproducibility along with other variables (the same as Experiment 1), and analyses are performed separately for beauty, liking, and enjoyability dependent/outcome variables.

## Results

### Experiment 1—Paintings

#### Rating task

**Figure 3:**
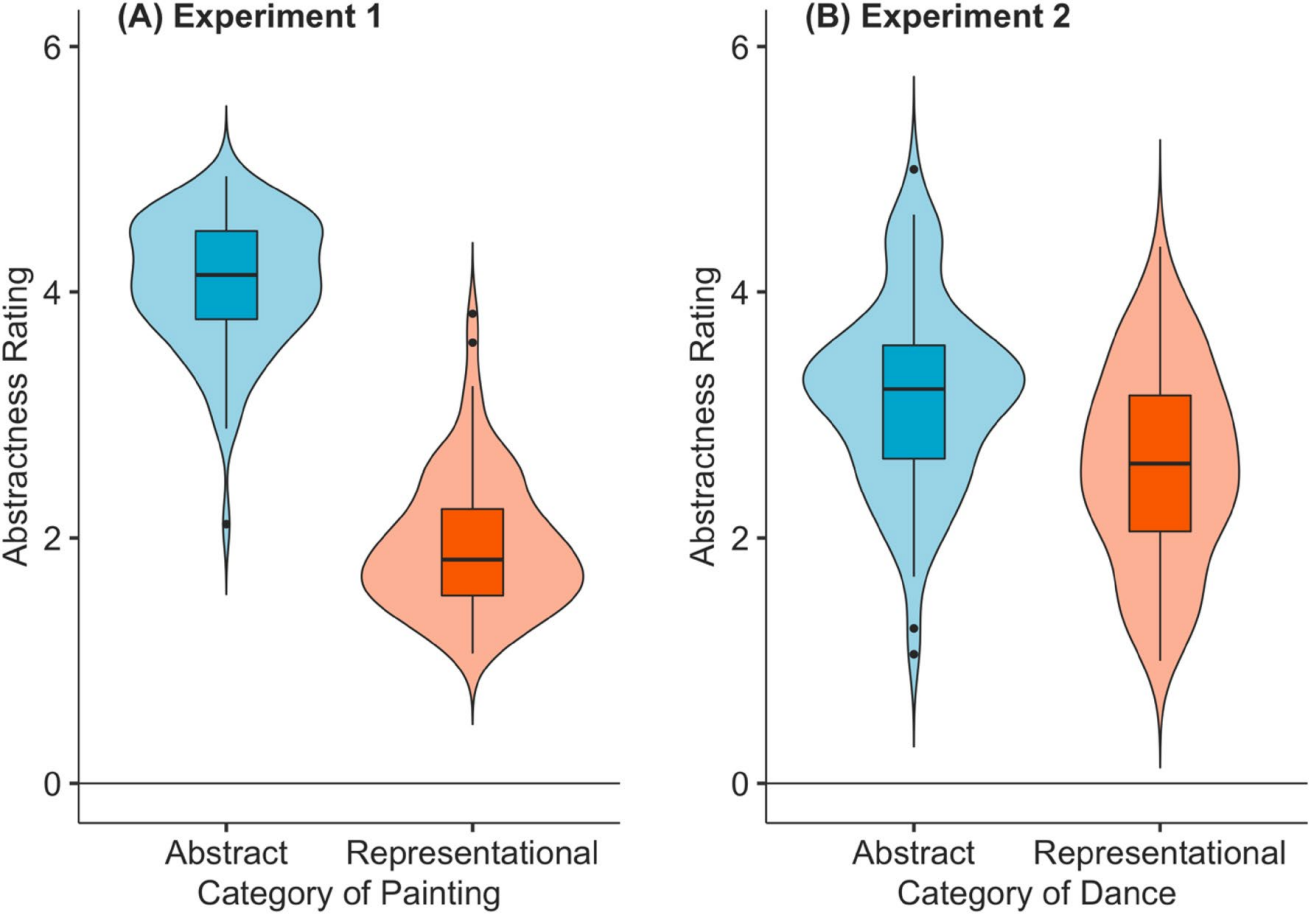
Abstractness ratings (on a 5-point likert scale where 1 = not at all abstract, and 5 = extremely abstract) for abstract and representational paintings (A; Experiment 1) and abstract and representational dance (B; Experiment 2).

Mean ratings for familiarity, complexity, evocativeness, technical competency, beauty, liking, and abstractness across abstract and representational art for experts and non-experts of Indian and Western cultures and Indian and Western art are provided in Table S3 and in Figures S2-S8. To check whether participants perceived abstract and representational paintings as more abstract and less abstract respectively, participants were also asked to rate paintings on abstractness. Overall, a paired samples t-test suggested that participants rated abstract paintings (Mean = 4.08, SD = 0.52) higher on abstractness compared to representational paintings (mean = 1.94; SD = 0.55; t(91)  = 29.44, *p* < 0.001, 95% CI [2.01, ∞]; see Fig. 3).

#### Categorisation task

**Figure 4:**
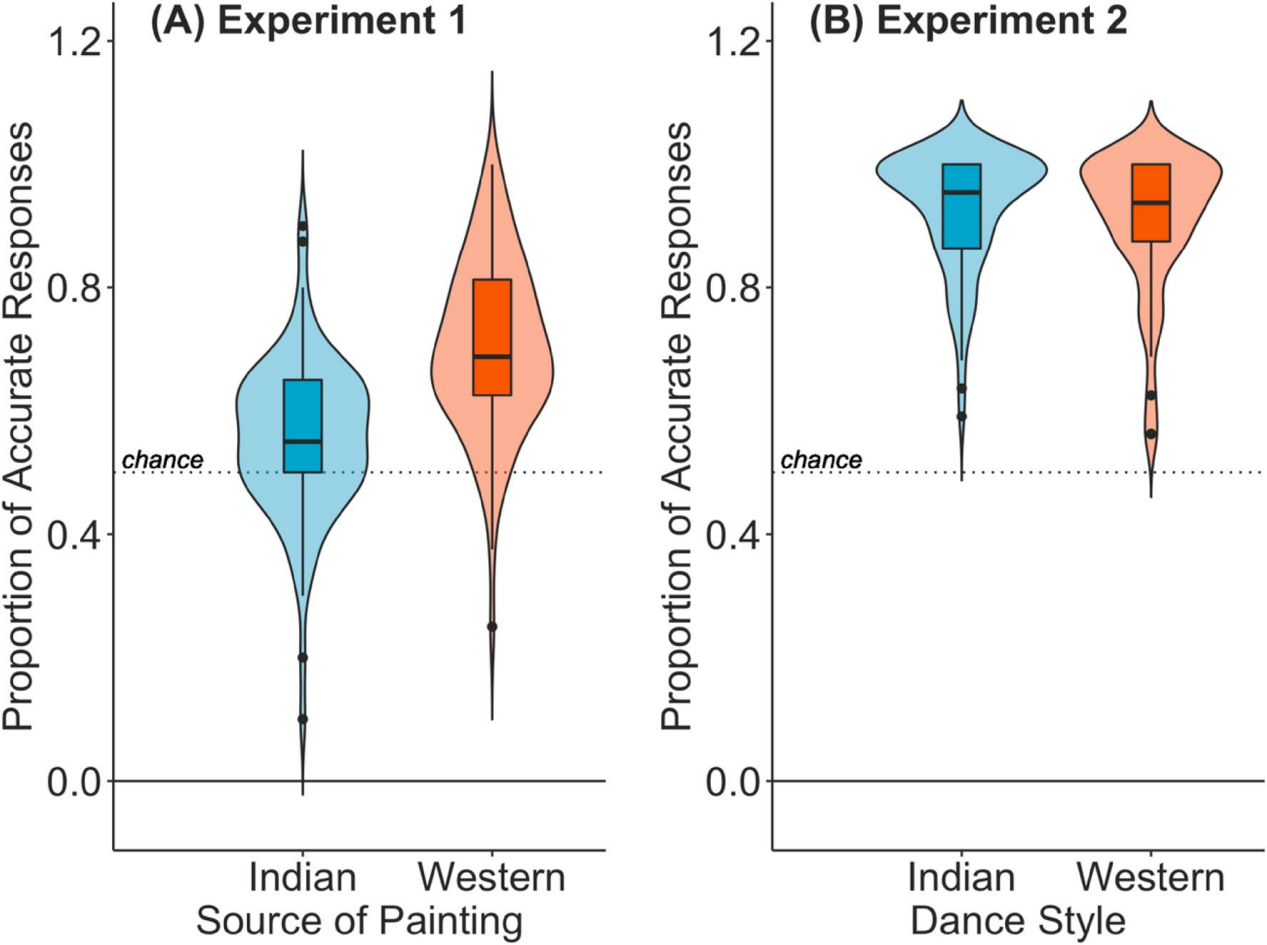
Proportion of accurate responses for Experiment 1 (**A**) and Experiment 2 (**B**) of all participants for Indian and Western paintings and dance styles. Dashed line represents 50% accuracy (or chance).

Accuracy on the categorisation task across category, source of painting, expertise, and culture are provided in Table S17. One sample t-tests suggested that for both Indian and Western paintings, participants could accurately categorise paintings as “Indian” or “Western” greater than chance (Indian paintings: Mean = 0.55, SD = 0.20, t(91) = 4.18, *p* < 0.001, 95% CI [0.53, ∞]; Western paintings: Mean = 0.70, SD = 0.24, t(91) = 14.06, *p* < 0.001, 95% CI [0.68, ∞]; see Fig. 4A). Accuracy for Western paintings was higher than Indian paintings (t (91) = 7.35, *p* < 0.001, 95% CI [0.19, 0.11]).

#### RQ1: Do expertise and culture shape aesthetic judgements of representational and abstract art (preregistered and confirmatory)?

**Figure 5:**
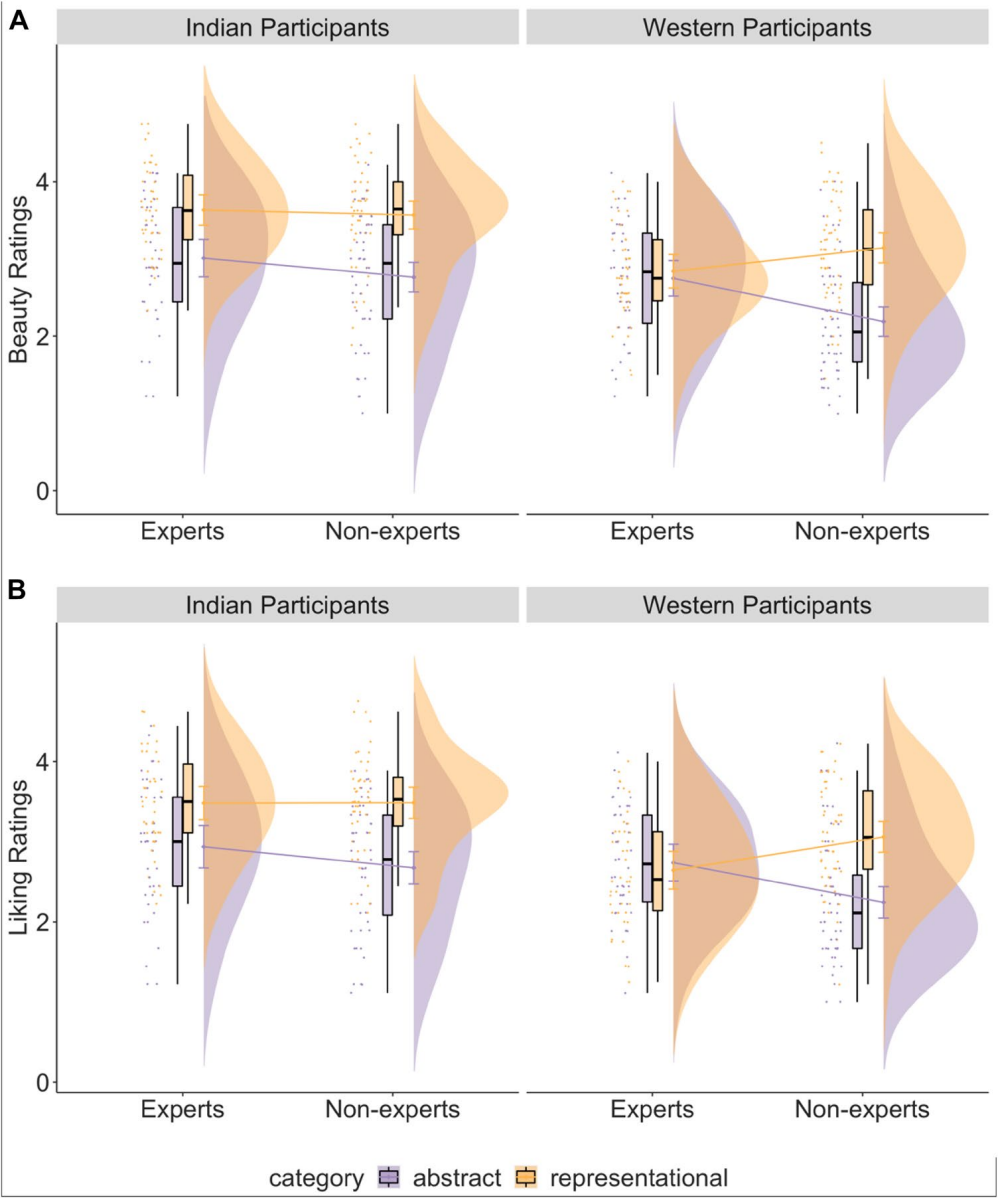
The effect of category (abstract or representational paintings), expertise (art experts or non-experts) and culture (Indian participants or Western participants) on the ratings of beauty (**A**) and liking (**B**).

The results of a cumulative link mixed effects model for beauty and liking ratings showed that category (beauty: β = 1.32, *p* < 0.001; liking: β = 1.01, *p* < 0.001), culture (beauty: β = 1.09, *p* < 0.001; liking: β = 0.91, *p* < 0.001) the interaction between category and expertise (beauty: β = 1.12, *p* < 0.001; liking: β = 1.14, *p* < 0.001), the interaction between culture and category (liking: β = 0.60, *p* = 0.04) and the three-way interaction between category, expertise, and culture (beauty: β = 1.41, *p* < 0.001; liking: β = 1.26, *p* = 0.04) had an effect on the ratings of beauty and liking (Table S4). Post-hoc tests revealed that all participants (except Western experts) showed higher ratings of beauty and liking for representational paintings compared with abstract paintings, and overall ratings by Indian participants were higher than Western participants (see Table S8). Specifically, to test our hypothesis whether the difference in beauty and liking ratings of abstract and representational paintings would be higher in non-experts compared to experts, we computed an interaction contrast for both Indian and Western culture participants separately. The contrast revealed that the difference between beauty and liking ratings for abstract and representational paintings was higher in non-experts than experts, but only for Western participants (beauty: M = 1.83, SE = 0.42, *p* < 0.001; liking: M = 1.77, SE = 0.44, *p* < 0.001) and not for Indian participants (beauty: M = 0.42, SE = 0.43, *p* = 0.33, Fig. 5A; liking: M = 0.51, SE = 0.43, *p* = 0.24, Fig. 5B).

As our study was powered to detect a two-way interaction between category and expertise, we additionally ran a model to test for this interaction for Indian and Western culture participants separately:

$${\text{clmm(beauty}}/{\text{liking}} \sim {\text{category}}*{\text{expertise}} + (1 + {\text{category}}|{\text{subject}}) + (1 + {\text{expertise}}|{\text{item}}),{\text{link}} = {\text{``}}{\mathrm{logit}}{\text{''}},{\text{threshold}} = {\text{``}}{\mathrm{flexible}}{\text{''}})$$

Similar to the above findings, we found that beauty and liking ratings were modulated by an interaction between category and expertise only for Western participants (beauty: β = 1.76, *p* < 0.001; liking: β = 1.66, *p* < 0.001), and not for Indian participants (β = 0.44, *p* = 0.32; liking: β = 0.53, *p* = 0.25; see Table S7).

**Figure 6:**
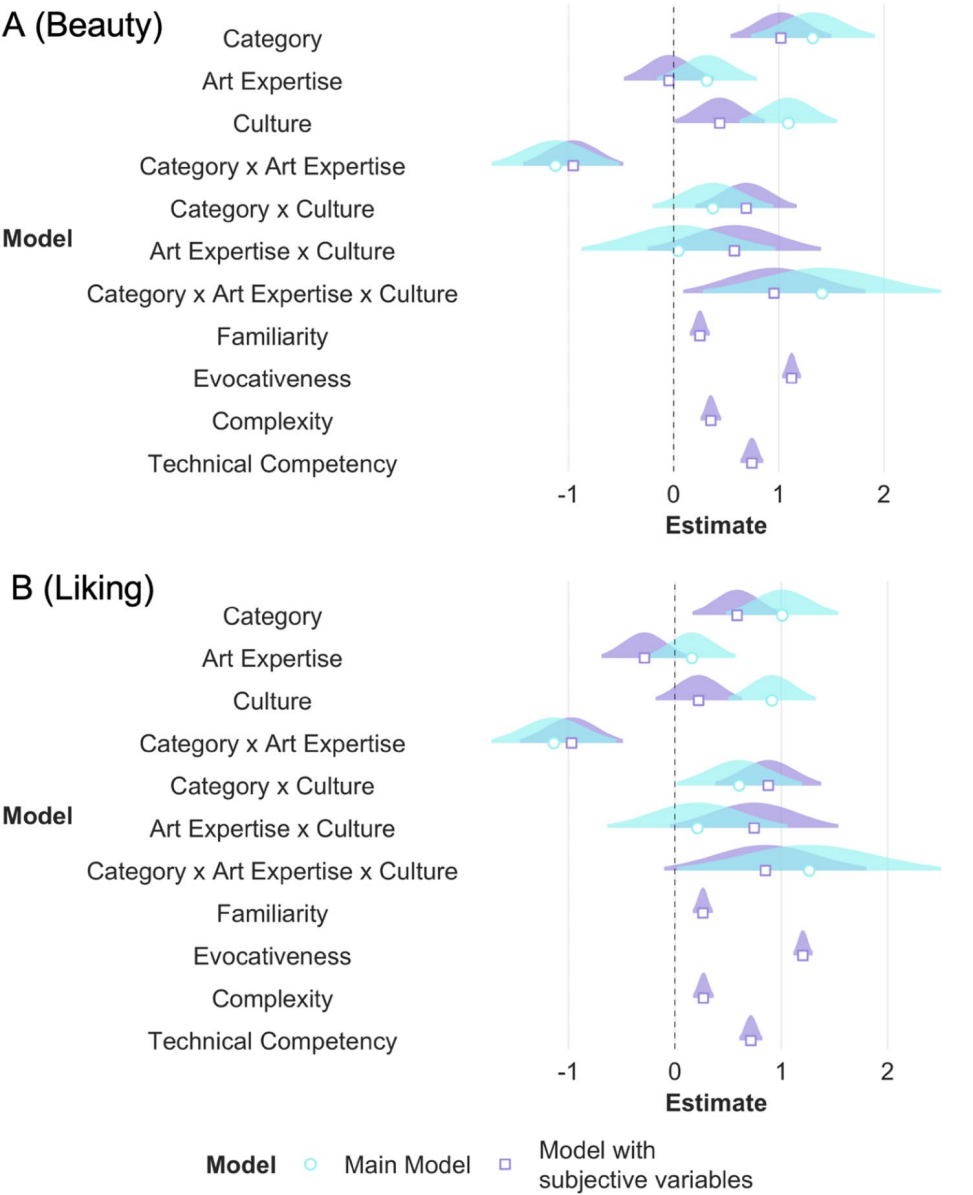
For the outcome variables of beauty (**A**) and liking (**B**), beta estimates for the main model (in aqua blue) and the model with subjective variables (in purple) are plotted for each predictor variable along with their corresponding uncertainties (95% confidence interval width for a normal distribution for each estimate). Distributions are rescaled to match the height of each distribution. This figure (and other similar figures) is made using the plot_summs function in the jtools R package (v. 2.1.0; Long, 2020).

Additionally, we added the subjective variables of familiarity, evocativeness, complexity, and technical competency as fixed effects to our main model to investigate whether the three-way interaction modulated beauty and liking ratings irrespective of the contribution of the subjective variables. As expected, all subjective variables predicted ratings of beauty and liking, with higher familiarity, complexity, evocativeness, and technical competency predicting higher ratings of beauty and liking. Adding the subjective variables significantly improved our main models (beauty: AIC_main_ = 8492.53, AIC_subj_ = 7035.66, *p* < 0.001; liking: AIC_main_ = 8893.61, AIC_subj_ = 7391.98, *p* < 0.001) and the three-way interaction of category, expertise, and culture influenced ratings of beauty even when accounting for possible contributions of subjective variables. For liking ratings, the three-way interaction estimate confidence intervals partially overlapped with zero but still influenced ratings of liking even when accounting for possible contributions of subjective variables (see Table S4; Fig. 6A,B).

#### RQ2: Does expertise influence the ingroup bias for aesthetic judgements (preregistered but exploratory)?

**Figure 7:**
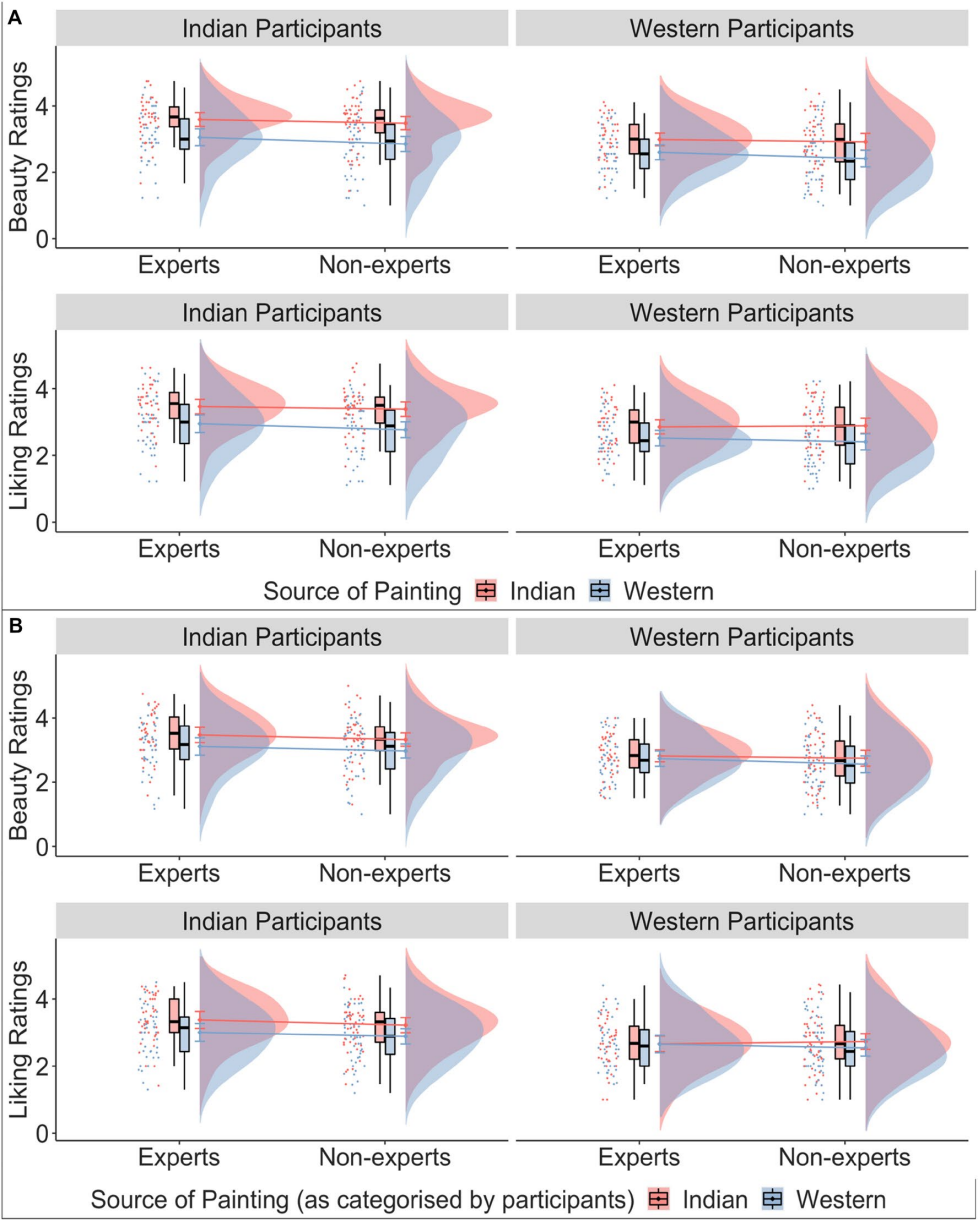
The effect of source of painting (A) and the source of painting as categorized by participants (B; Indian or Western paintings depicted in red and blue respectively), expertise (art experts or non-experts) and culture (Indian participants or Western participants) on the ratings of beauty and liking.

The results of a cumulative link mixed effects model for beauty and liking ratings showed that source of painting (beauty: β = 1.05, *p* < 0.001; liking: β = 1.01, *p* < 0.001) and culture (beauty: β = 1.05, *p* < 0.001; liking: β = 0.91, *p* < 0.001) had an effect on the ratings of beauty and liking (Table S5). Specifically Indian paintings were liked more and rated as more beautiful than Western paintings by all participants. Indian participants (beauty: M = 0.43, SE = 0.23, 95% CI [− 0.03, 0.88]) showed higher ratings of beauty and liking for all paintings compared to Western participants (beauty: M = − 0.62, SE = 0.24, 95% CI [− 1.08, − 0.16]; *p* < 0.001). There was no evidence of an ingroup bias or its modulation by art expertise: there were no other significant main effects or two-way or three-way interactions (see Table S5, Fig. 7A,B).

**Figure 8:**
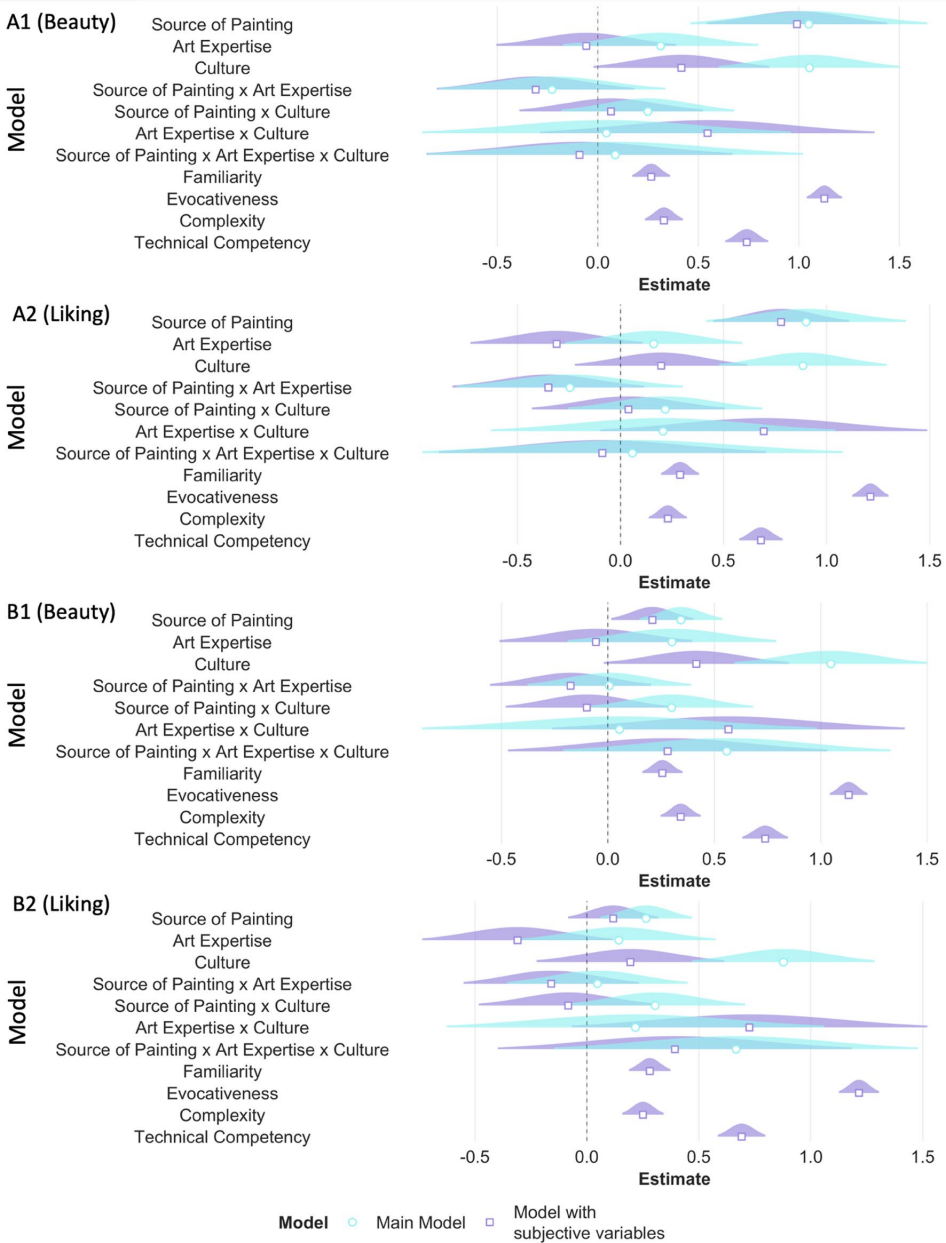
For the outcome variables of beauty and liking, beta estimates for the main model (in aqua blue) and the model with subjective variables (in purple) are plotted for each predictor variable along with their corresponding uncertainties (95% confidence interval width for a normal distribution for each estimate). Distributions are rescaled to match the height of each distribution. Figures A1 and A2 display models with source of painting as classified by the experimenter i.e., according to the origin of the artists. Figures B1 and B2 display models with source of painting as classified by the participants themselves.

Similar to RQ1, we ran an additional model adding the subjective variables of familiarity, evocativeness, complexity, and technical competency as fixed effects. As expected, all subjective variables influenced the ratings of beauty and liking. Adding the subjective variables significantly improved our main models (beauty: AIC_main_ = 8636.42, AIC_subj_ = 7087.03, *p* < 0.001; liking: AIC_main_ = 8893.61, AIC_subj_ = 7391.98, *p* < 0.001). The main effects of culture (although marginally significant) and source of painting still influenced beauty and liking ratings even when accounting for the possible contributions of the subjective variables (see Table S5, Fig. 8A,B).

**Figure 9:**
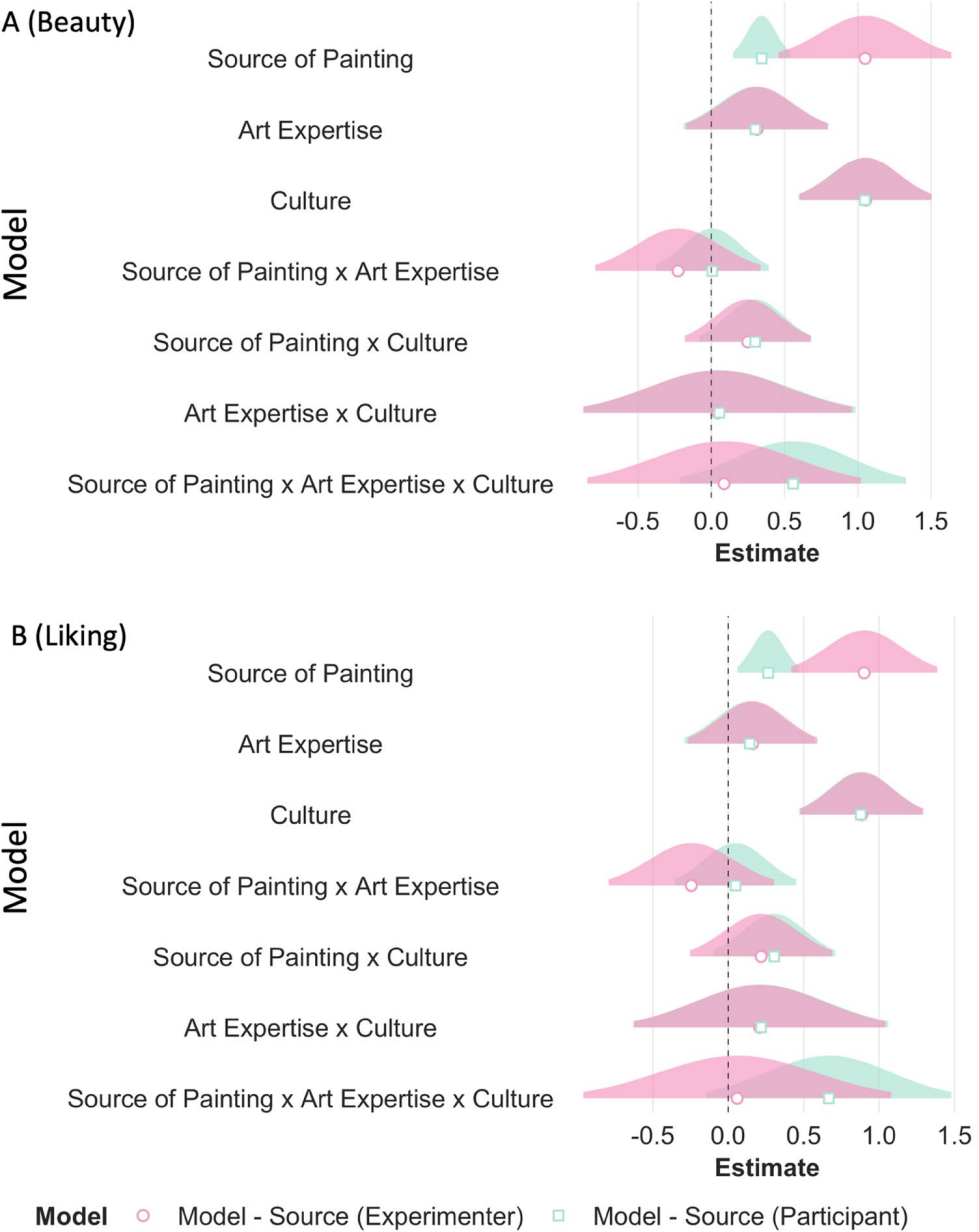
For the outcome variables of beauty (**A**) and liking (**B**), beta estimates for the model with source of painting as categorized by the experimenter (in pink) and the model with source of paintings as categorized by the participant (in green) are plotted for each predictor variable along with their corresponding uncertainties (95% confidence interval width for a normal distribution for each estimate). Distributions are rescaled to match the height of each distribution.

**Figure 10:**
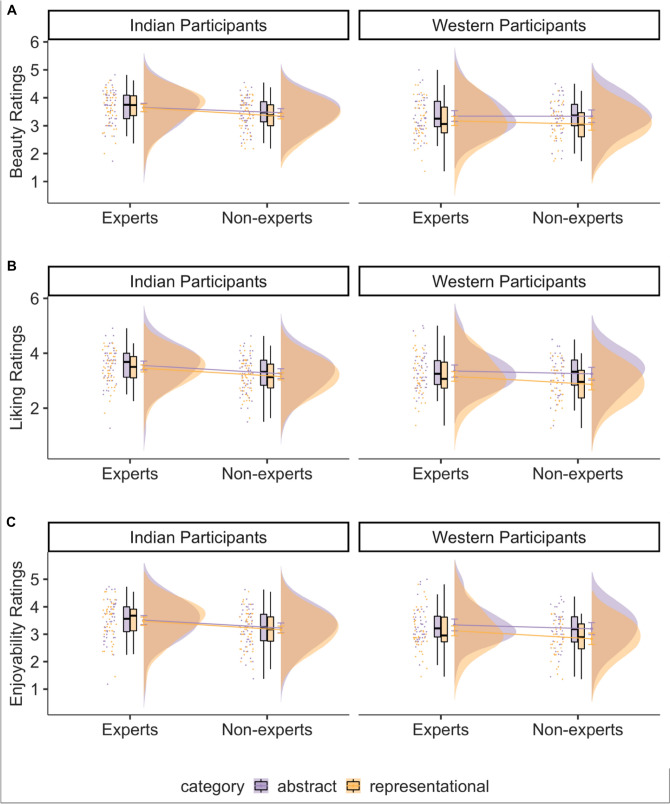
The effect of dance expertise (experts or non-experts) and culture (Indian or Western participants) on beauty, liking, and enjoyability ratings of abstract (in purple) and representational (in yellow) dance videos.

Results were similar when the factor ‘source of painting’ was coded as either ‘Indian’ or ‘Western’ as categorized by the individual participants in the Categorization task (see Table S6, Figs. 9 and 10). That is, source of painting and culture influenced beauty and liking ratings, but no other two-way or three-way interactions were found. However, a visual inspection of the data suggests that although a three-way interaction did not pass our statistical threshold (i.e. *p* < 0.05), a trend for a three way interaction can be observed such that Western experts no longer showed higher ratings of beauty and liking for Indian paintings when paintings were categorized by the participants (see Figs. 7A,B, 9A,B).

### Experiment 2—Dance

#### Rating task

Mean ratings for familiarity, complexity, evocativeness, technical competency, reproducibility, and abstractness across abstract and representational dance for experts and non-experts of Indian and Western cultures for Bharatanatyam and ballet dance styles are provided in Table S11 and in Figures S10-S17. To check whether participants perceived abstract and representational dance as more abstract and less abstract respectively, participants were also asked to rate the dance videos on abstractness. Overall, a paired-samples t-test suggested that participants rated abstract dance videos (Mean = 3.16, SD = 0.76) higher on abstractness compared to representational dance (mean = 2.59; SD = 0.79; t(89) = 4.81, *p* < 0.001, 95% CI [0.37, ∞]; see Fig. 3B).

#### Categorisation task

Accuracy across dance style, category, expertise, and culture are provided in Table S18. One sample t-tests suggested that for both Indian and Western dance styles, participants could accurately categorise dance videos as “Bharatanatyam” or “ballet” greater than chance (Bharatanatyam: Mean = 0.93, SD = 0.14, t(89) = 43.08, *p* < 0.001, 95% CI [0.91, ∞]; ballet: Mean = 0.91, SD = 0.16, t(89) = 35.24, *p* < 0.001, 95% CI [0.89, ∞]; see Fig. 4B). There was no difference in accuracy for Bharatanatyam or ballet dance styles (t (89) = 1.13, *p* = 0.26, 95% CI [− 0.01, 0.05]).

#### RQ1: Do expertise and culture shape aesthetic judgements of representational and abstract dance (preregistered and confirmatory)?

The results of a cumulative link mixed effects model for beauty, liking, and enjoyability ratings showed that culture (beauty: β = 0.75, *p* = 0.004; liking: β = 0.46, *p* = 0.07; enjoyability: β = 0.50, *p* = 0.06), category (beauty: β = 0.41, *p* = 0.06; liking: β = 0.51, *p* = 0.01; enjoyability: β = 0.43, *p* = 0.03) and dance expertise (beauty: β = 0.42, *p* = 0.09; liking: β = 0.62, *p* = 0.01; enjoyability: β = 0.63, *p* = 0.01) had an impact on the ratings of beauty, liking, and enjoyability. Ratings by Indian participants (beauty: M = 1.22, SE = 0.19, 95% CI [0.84, 1.59]; liking: M = 0.745, SE = 0.19, 95% CI [0.37, 1.10]; enjoyability: M = 0.75, SE = 0.20, 95% CI [0.36, 1.14]) were overall higher than ratings made by Western participants (beauty: M = 0.46, SE = 0.21, 95% CI [0.04, 0.88], *p* = 0.004; liking: M = 0.28, SE = 0.20, 95% CI [− 0.12, 0.68]; enjoyability: M = 0.25, SE = 0.21, 95% CI [− 0.16, 0.66]). Abstract dance videos (beauty: M = 1.04, SE = 1.87, 95% CI [0.68, 1.41]; liking: M = 0.76, SE = 0.18, 95% CI [0.41, 1.11]; enjoyability: M = 0.72, SE = 0.18, 95% CI [0.36, 1.08]) were rated as more beautiful, more enjoyable, and were liked more than representational dance videos (beauty: M = 0.63, SE = 0.19, 95% CI [0.26, 1.01], *p* = 0.06; liking: M = 0.25, SE = 0.18, 95% CI [− 0.10, 0.61]; enjoyability: M = 0.28, SE = 0.19, 95% CI [− 0.08, 0.65]). Dance experts (beauty: M = 1.05, SE = 0.19, 95% CI [0.68, 1.43]; liking: M = 0.82, SE = 0.19, 95% CI [0.45, 1.18]; enjoyability: M = 0.82, SE = 0.20, 95% CI [0.43, 1.20]) rated all dance videos higher on beauty, liking, and enjoyability than non-dancers (beauty: M = 0.63, SE = 0.21, 95% CI [0.22, 1.04], *p* = 0.09; liking: M = 0.20, SE = 0.20, 95% CI [− 0.20, 0.59]; enjoyability: M = 0.18, SE = 0.21, 95% CI [− 0.23, 0.59]). No other two-way or three-way interactions emerged for beauty and liking ratings (see Table S12 and Fig. 10A–C). A two-way interaction between category and culture influenced enjoyability ratings (enjoyability: β = 0.51, *p* = 0.05). Post hoc tests revealed that abstract dance videos (M = 0.59, SE = 0.23, 95% CI [0.13, 1.05]) were rated as more enjoyable compared to representational dance videos (M = − 0.09, SE = 0.24, 95% CI [− 0.57, 0.38], *p* = 0.005) only by Western participants and not by Indian participants.

As our study was powered to detect a two-way interaction between category and expertise, we additionally ran a model to test for this interaction for Indian and Western culture participants separately:

$${\text{clmm}}({\text{beauty}}/{\text{liking}}/{\text{enjoyability}}\sim {\text{category}}*{\text{expertise}} + (1 + {\text{category}}|{\text{subject}}) + (1 + {\text{expertise}}|{\text{item}}),{\text{link}} = {\text{``}}{\mathrm{logit}}{\text{''}},{\text{threshold}} = {\text{``}}{\mathrm{flexible}}{\text{''}})$$

For Indian participants, dance expertise had a marginal effect on ratings of beauty, liking, and enjoyability, with expert dancers showing higher ratings than non-dancers (beauty: β = 0.59, *p* = 0.08; liking: β = 0.68, *p* = 0.04; enjoyability: β = 0.67, *p* = 0.07), and for Western participants, abstract dance videos were rated as more beautiful, were liked more, and were rated as more enjoyable than representational dance videos (beauty: β = 0.58, *p* = 0.04; liking: β = 1.01, *p* = 0.005; enjoyability: β = 0.69, *p* = 0.054; see Table S15). No two-way interaction between category and art expertise emerged.

**Figure 11:**
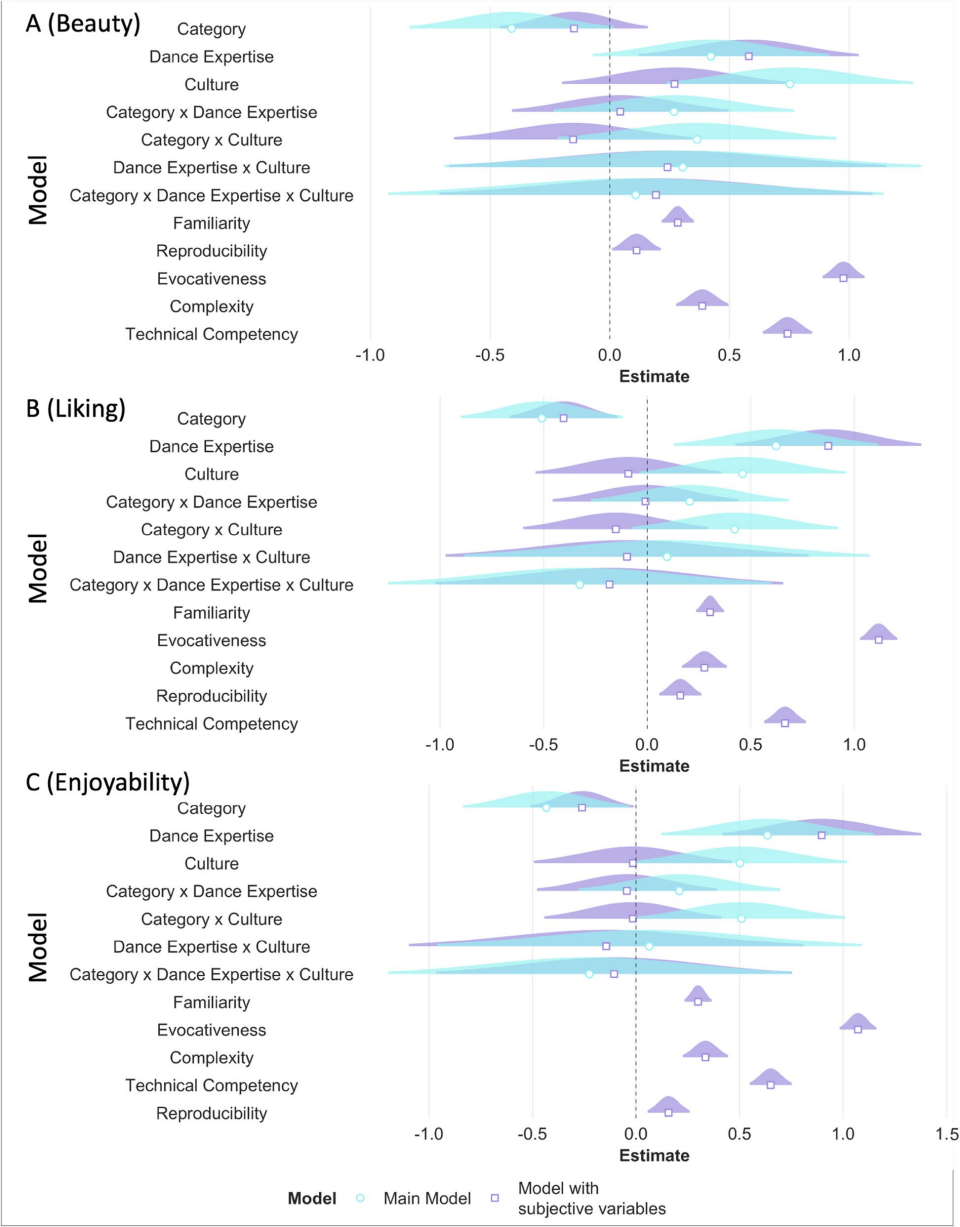
RQ1: The effect of category, expertise, and culture. For the outcome variables of beauty (**A**), liking (**B**), and enjoyability (**C**) beta estimates for the main model (in aqua blue) and the model with subjective variables (in purple) are plotted for each predictor variable (fixed effect) along with their corresponding uncertainties (95% confidence interval width for a normal distribution for each estimate). Distributions are rescaled to match the height of each distribution.

Additionally, we added the subjective variables of familiarity, evocativeness, complexity, reproducibility, and technical competency as fixed effects to our full model to investigate whether the main effects of category, expertise, and culture modulated beauty, liking, and enjoyability ratings irrespective of the contribution of the subjective variables. As expected, all subjective variables predicted ratings of beauty, liking, and enjoyability with higher familiarity, complexity, evocativeness, reproducibility, and technical competency predicting higher ratings of beauty, liking, and enjoyability. Adding the subjective variables significantly improved our main models (beauty: AIC_main_ = 8469.90, AIC_subj_ = 7001.71, *p* < 0.001; liking: AIC_main_ = 8862.6, AIC_subj_ = 7307.13, *p* < 0.001; enjoyability: AIC_main_ = 8889.0, AIC_subj_ = 7366.1, *p* < 0.001). Dance expertise influenced beauty, liking and enjoyability ratings, and category influenced liking and enjoyability ratings, even when accounting for possible contributions of subjective variables. In this particular analysis, we did not find that culture particularly influenced beauty, liking, and enjoyability ratings suggesting that the effects of subjective variables could perhaps explain the effect of culture seen in the main models (see Table S12, Fig. 11A–C).

#### RQ2: Does expertise influence the ingroup bias for aesthetic judgements (preregistered but exploratory)?

**Figure 12:**
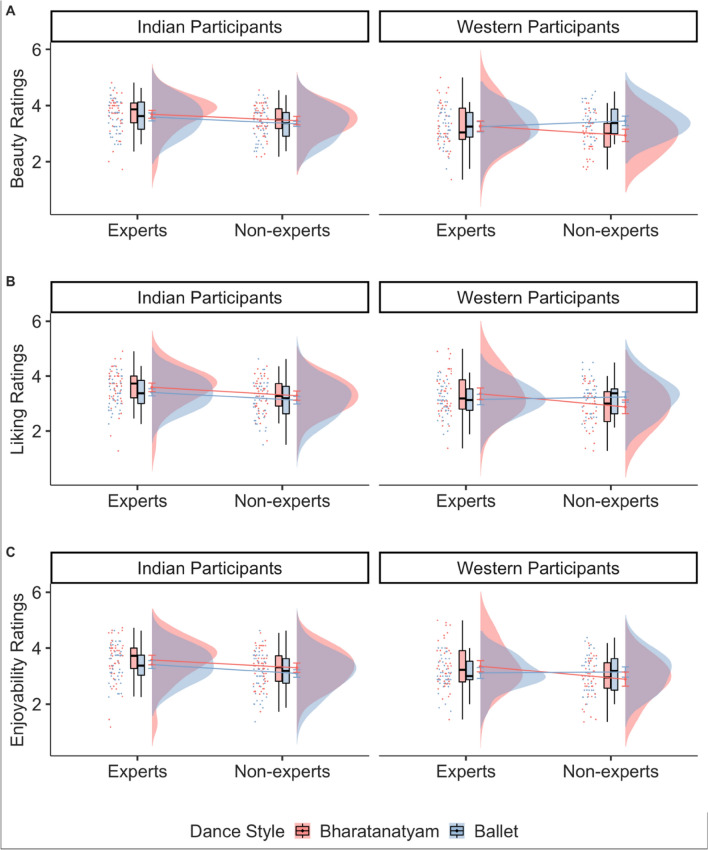
The effect of dance style (Bharatanatyam or ballet depicted in red and blue respectively), expertise (art experts or non-experts) and culture (Indian participants or Western participants) on the ratings of beauty (**A**), liking (**B**), and (**C**) enjoyability.

The results of a cumulative link mixed effects model for beauty and liking ratings showed that culture (beauty: β = 0.70, *p* = 0.006; liking: β = 1.01, *p* = 0.09; enjoyability: β = 0.47, *p* = 0.07), dance expertise (liking: β = 0.58, *p* = 0.02; enjoyability: β = 0.60, *p* = 0.02) the two-way interactions of dance style and expertise (beauty: β = 0.65, *p* = 0.01; liking: β = 0.69, *p* = 0.02; enjoyability: β = 0.53, *p* = 0.06), and dance style and culture (beauty: β = 0.77, *p* = 0.006) and the three way interaction of dance style, expertise, and culture (beauty: β = 1.32, *p* = 0.01; liking: β = 1.30, *p* = 0.02; enjoyability: β = 1.36, *p* = 0.01) had an effect on the ratings of beauty, liking, and enjoyability (Table S13). Specifically, while the two-way culture by dance style interaction suggests that Western participants rated ballet as more beautiful than Bharatanatyam, and Indian participants rated Bharatanatyam as more beautiful than ballet, post hoc tests revealed than an ingroup bias was present such that only Western non-experts showed higher ratings of beauty, liking, and enjoyability for ballet (beauty: M = 1.01, SE = 0.34, 95% CI [0.34, 1.67]; liking: M = 0.46, SE = 0.35, 95% CI [− 0.24, 1.15] ; enjoyability: M = 0.27, SE = 0.34, 95% CI [− 0.39, 0.93]) compared to Bharatanatyam (beauty: M = − 0.18, SE = 0.35, 95% CI [− 0.86, 0.50], *p* < 0.001; liking: M = − 0.35, SE = 0.37, 95% CI [− 1.08, 0.38], *p* = 0.05; enjoyability: M = − 0.31, SE = 0.37, 95% CI [− 1.03, 0.42], *p* = 0.05; see Table S13, Fig. 12A–C).

**Figure 13:**
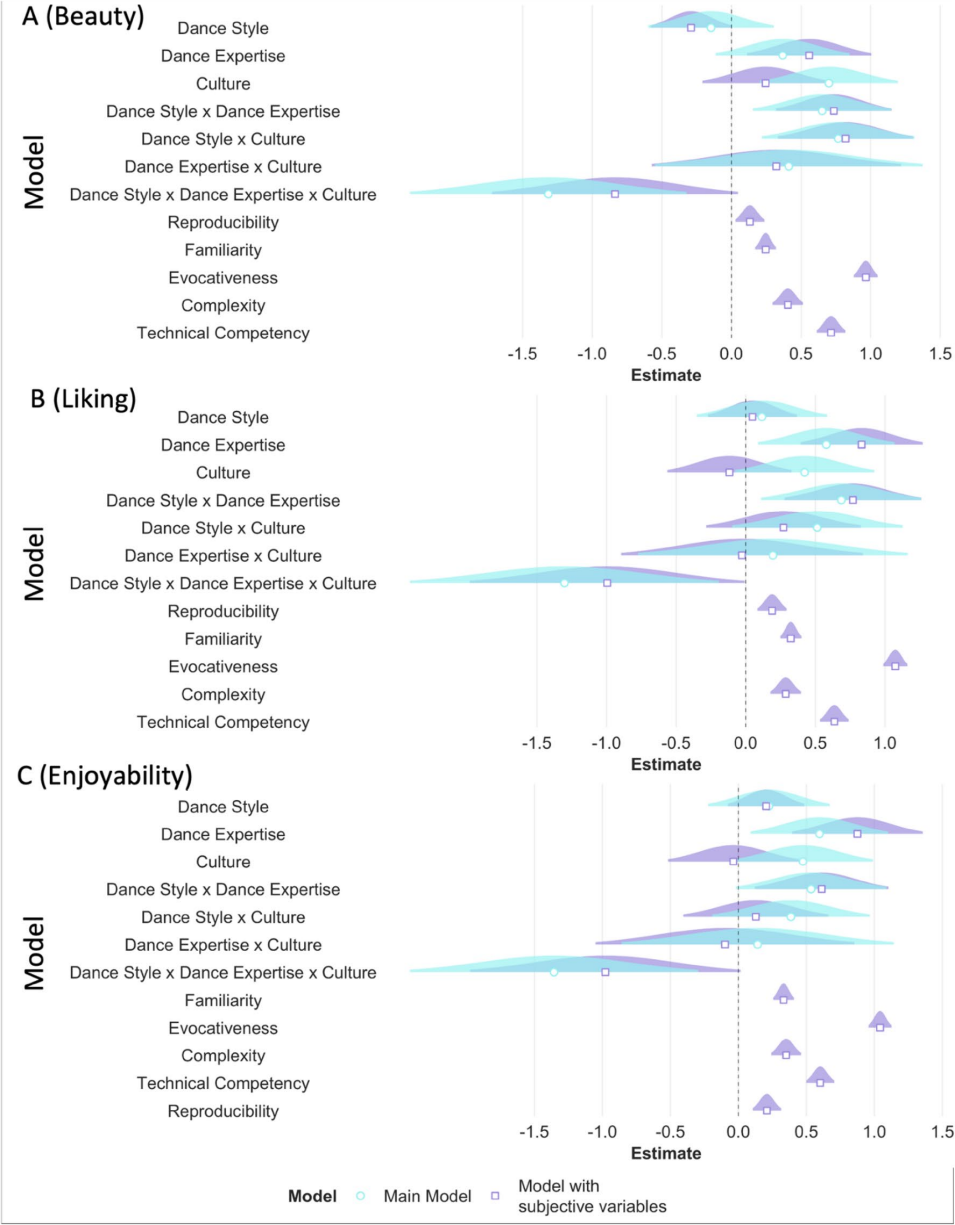
RQ2: The effect of Expertise, Culture, and Dance Style. For the outcome variables of beauty (**A**), liking (**B**), and enjoyability (**C**) beta estimates for the main model (in aqua blue) and the model with subjective variables (in purple) are plotted for each predictor variable along with their corresponding uncertainties (95% confidence interval width for a normal distribution for each estimate). Distributions are rescaled to match the height of each distribution.

Similar to RQ1, we ran an additional model adding the subjective variables of familiarity, evocativeness, complexity, reproducibility, and technical competency as fixed effects. As expected, all subjective variables influenced the ratings of beauty and liking such that higher ratings of familiarity, evocativeness, complexity, technical competency, and reproducibility predicted higher ratings of beauty and liking. Adding the subjective variables significantly improved our main models (beauty: AIC_main_ = 8636.42, AIC_subj_ = 6977.28, *p* < 0.001; liking: AIC_main_ = 8743.3, AIC_subj_ = 7241.4, *p* < 0.001; enjoyability: AIC_main_ = 8814.7, AIC_subj_ = 7307.7, *p* < 0.001). Importantly, the three-way interaction still influenced beauty, liking, and enjoyability ratings even when accounting for the possible contributions of the subjective variables (beauty: β = 0.84, *p* = 0.06; liking: β = 0.99, *p* = 0.05; enjoyability: β = 0.98, *p* = 0.05); see Table S13, Fig. 13A–C).

Results were similar when the factor ‘dance style’ was coded as either ‘Bharatanatyam’ or ‘ballet’ as categorized by the individual participants in the Categorization task (see Table S14, Figures S19-21).

### Art expertise and art experience questionnaires

We also computed art/dance expertise scores using the Art Expertise Questionnaire for paintings (art expertise, Table S19) and dance (dance expertise, Table S20) between Indian and Western experts and non-experts for Experiments 1 and 2. Independent samples t-tests suggested that experts (Experiment 1: Mean = 31.43, SD = 9.66; Experiment 2: Mean = 14.44, SD = 5.13) scored higher than non-experts (Experiment 1: Mean = 14.02, SD = 5.94; Experiment 2: Mean = 8.95, SD = 4.79) on the art expertise questionnaires (Experiment 1: t(90) = 10.58, *p* < 0.001, 95% CI [14.14, 20.68]; Experiment 2: t(88) = 5.25, *p* < 0.001, 95% CI [3.41, 7.57]). Expertise scores for Indian experts (Experiment 1: Mean = 31.19, SD = 9.65; Experiment 2: Mean = 14.48, SD = 4.91) and Western experts (Experiment 1: Mean = 31.67, SD = 9.91; Experiment 2: Mean = 14.41, SD = 5.47) were not significantly different from each other (Experiment 1: t(40) =  − 0.16, *p* = 0.87, 95% CI [− 6.57, 5.62]; Experiment 2: t(43) = 0.04, *p* = 0.96, 95% CI [− 3.05, 3.19]).

### Demographic variables

For both Experiments 1 and 2, and RQ1 and RQ2, our findings were similar even when accounting for the demographic variables of age and education. That is, even when age and level of education were included in the models as control variables, our findings were similar to those reported above. We do not report these analyses in the main manuscript as they were not a part of our preregistered analyses but provide these data online for researchers to pursue related questions of interest.

## Discussion

The aim of the current work was to evaluate the extent to which people show universal aesthetic preferences across fine and performing arts, and to examine the extent to which one’s cultural background and experience with an art form shape these preferences. Across two experiments analysed using mixed effects models, we investigated whether the nature of aesthetic preference and the role of expertise in aesthetic judgements generalises across artforms (paintings and dance) and cultures (Indian, Western). As hypothesized, we found a preference for representational art (only for paintings) and an ingroup bias (only for dance), both modulated by painting/dance expertise. In both cases, however, the modulation by expertise emerged only among Western participants, and both the preference for representational art and an ingroup bias for aesthetic judgement did not manifest across artforms (paintings and dance) in a similar manner. In the following sections, we evaluate and position our findings within a broader context, and discuss implications for the field of empirical aesthetics in particular, and psychology more generally.

### The preference for representational art and its modulation by expertise

In Experiment 1, we found that participants belonging to either Indian or Western cultures prefer representational paintings more than abstract paintings. Representational paintings were assigned higher ratings of beauty and liking compared to abstract paintings, consistent with previous research reporting a preference for representational compared to abstract images and paintings^6,8,17,21–23^. Furthermore, art expertise modulated beauty and liking ratings for representational and abstract paintings in the current study. As hypothesized, and in accordance with previous work, beauty and liking rating differences between representational and abstract art were smaller among art experts compared to non-experts^17,24–26^. This modulation, however, was present only among our Western participant sample. While both Indian experts and non-experts showed higher ratings of beauty and liking for representational paintings compared to abstract paintings, the difference between ratings for representational and abstract paintings did not differ across Indian experts compared to non-experts. In other words, the preference for representational paintings was more attenuated for Western experts compared to Western art naïve participants, but this attenuation did not emerge among the expert Indian participants.

While we expected a similar pattern of results across Indian and Western participants, to the best of our knowledge, all prior research investigating the modulation of art expertise on aesthetic ratings of abstract and representational art has solely examined participants from Northern America or Western Europe. The modulation of art expertise could represent a lower preference for representational art by experts compared to non-experts, a higher preference for abstract art by experts compared to non-experts, or a combination of both. Figure 5 suggests that the interaction between category of the painting (representational or abstract) and the expertise of the participants (experts or non-experts) in Western compared to Indian participants might be driven by lower ratings of beauty and liking for representational paintings by Western experts (or higher ratings for representational paintings by Indian participants).

One possible explanation for the three-way interaction between culture, category, and expertise could therefore be the familiarity and representativeness of the paintings for Indian and Western participants. It is possible that Indian participants are more familiar with the representative content of both Indian and Western paintings to a greater extent because of their exposure to Western culture in mainstream media^7^. Further, Western art forms a major part of the syllabus for art education in India, whereas exposure to Indian art and culture may be more limited in Western art education^67^. Indian participants may consequently report higher ratings for representational paintings from both Indian and Western painters compared to Western participants if we assume that higher familiarity with a painting leads to higher aesthetic ratings. While this explanation seems unlikely as the three-way interaction of culture, category, and expertise holds even when accounting for the contribution of the familiarity of the painting to the spectator, it remains possible that the meaningfulness of the representative content differs for Indian and Western art experts. Another possible explanation is that Indian experts do not show a preference for abstract art to the same degree as Western participants. Indeed, Fig. 5 suggests that the difference between Indian expert and non-expert ratings of abstract paintings is lower compared to Western participants. A key point of consideration here is the abstract-representational categorisation of paintings, which is not perceived similarly across cultures. In contrast to the Euro-American idea that abstraction is purely a formal phenomenon (related to the form and composition of a painting), abstraction in Indian art is considered more of a rupture in a narrative or a form of symbolism^68^. Therefore, it is possible that Indian experts might have perceived all abstract paintings differently than Western expert participants. An additional point of consideration here is the personality dimension of openness to experience. Previous evidence suggests an association between openness to experience and a preference for abstract art as well as novelty in art^69^. It is possible that some of our between-culture differences can be explained by this personality construct i.e., Indian art experts may score lower on the dimension of openness to experience, and therefore show a smaller preference for abstract art compared to western art experts who score higher on this dimension. However, previous research provides mixed evidence for cross-cultural differences in openness to experience^70,71^. Similarly, socioeconomic status and social class have been known to influence aesthetic preferences^72^. An important avenue for future research would be to explore the link between preferences for abstract art and their link to individual differences such as social class, socioeconomic status, and the personality dimension of openness to experience across different cultures.

In contrast to the findings from Experiment 1, we did not find a preference for representational dance in Experiment 2. Instead, participants from both Indian and Western cultures rated abstract Indian and Western dance videos higher on ratings of beauty and liking compared to representational Indian and Western dance videos. This preference for abstract dance was not modulated by expertise or culture, except for a modulation of enjoyability ratings by culture. Specifically, western participants enjoyed abstract ballet more than representational ballet (but Indian participants did not show this preference). Replicating previous findings that suggest experts show higher aesthetic ratings for artworks (e.g.,^25,72^), we also found a main effect of dance expertise such that dance experts belonging to either Indian or Western culture reported overall higher ratings for the dance videos than dance naïve participants (c.f.^16^). Indian participants showed higher overall ratings of beauty, liking, and enjoyability for all dance videos compared to Western participants.

While we preregistered an expectation for similar findings be across both paintings and dance, our results are perhaps not that surprising, given differences between the fine arts and performing arts. Researchers have argued that “all visual art must obey the laws of the visual system”^73^ implying common neurobiological underpinnings of perceiving paintings and dance^74^. However, painting is a static form whereas dance is dynamic, although both can convey cultural and social stories and contexts, and have the potential to evoke aesthetic responses^75^. Further, representativeness these two art forms is conveyed differently. Figure 3A,B suggest that both abstract and representational paintings and dance videos were rated as significantly different from each other on abstractness ratings. But representational paintings can be a realistic representation of the visual world, and dance may convey objects or characters in the outside world using symbols and gestures, making its abstractness or representativeness more ambiguous, especially for dance-naïve spectators. Indeed, in our study, the difference in abstractness ratings between abstract and representational art was higher in paintings than in dance, suggesting a more clear-cut categorisation between abstract and representational paintings compared to abstract and representational dance. At the centre of much debate, especially in the context of Western ballet and modern dance, has been the distinction between abstract and representational dance^76–78^. While existing in theory, its mutual exclusiveness in actual practice (or performance) is more difficult for viewers to discern. In other words, even in the most narrative, dramatic, and expressive sequence, the structure of the choreography is driven both by formal, abstract features as well as its narrative content. In the same vein, any abstract dance will still involve some communication, representation, or expression, also because it involves a human body in motion (and the perception of the human body in motion by the spectators^79^).

### The ingroup bias and its modulation by expertise

Contrary to previous research and our predictions, we found no evidence of an ingroup bias for paintings in Experiment 1. Participants did not assign higher ratings to paintings belonging to their own cultural background compared to those belonging to another cultural background. Instead, overall aesthetic ratings assigned by Indian participants were higher than those assigned by Western participants, and Indian paintings overall were rated higher on beauty and liking compared to Western paintings by both Indian and Western participants. Findings were similar when the source of painting was categorised by the participants themselves. However, Fig. 7 shows that while all participants rated Indian paintings higher on beauty and liking, the difference in ratings between Indian and Western paintings among Western participants reduced when participants themselves categorised the paintings as ‘Indian’ or ‘Western.’ This observation suggests an attenuation of the preference for Indian paintings among Western participants when cultural background is explicitly referenced (although it remains important to note that an interaction between culture and source of painting was not statistically significant). The current results overall point toward an absence of the ingroup bias when aesthetically evaluating paintings, both when participants were not explicitly aware that the paintings belonged to their cultural background, and when they identified the painting as belonging to their own cultures.

Our results contradict previous (albeit limited) research that suggests people prefer artworks belonging to their own culture or country^6,43,80^. Indian paintings used in the current study were more contemporary than Western paintings. Therefore, it is possible that more recent paintings were perhaps more relatable and therefore showed higher ratings of beauty and liking. However, all paintings were matched on mean luminance, and the absence of ingroup bias persisted even when controlling for other subjective variables such as familiarity, evocativeness, complexity, and technical competency, and accounted for within-participant and within-item variance in our mixed effects models. While it is unlikely that an absence of an ingroup bias, and higher overall ratings for Indian paintings compared to Western paintings by both Indian and Western participants, were due to differences between Indian and Western paintings on luminance or subjective variables, it remains possible that other low-level features such as contrast, symmetry, and so on may explain differences between Indian and Western paintings.

We further speculate that Indian participants assigned overall higher ratings because they have more experience with both Indian and Western paintings (compared to Western participants for whom the content and style of the Indian paintings is relatively less familiar; ^67^). It is however also possible that overall higher ratings for Indian paintings by both Indian and Western participants can also be a result of more openness toward Indian culture by Western participants. Given the growing multicultural natures of western societies, it is plausible that Western cultures in general are more open or exposed to non-western cultures^81^. Another possible explanation is the “uncertainty-identity” hypothesis proposed by Mastandrea and colleagues^80^. In their recent study, Mastandrea and colleagues propose that national identity may be used to a greater extent as a heuristic to evaluate art when there is uncertainty about oneself. That is, when participants are less experienced or less familiar with art but still need to form an artistic opinion, they resort to group identification to resolve their uncertainty. In the current work, it is possible that participants in the first experiment (both experts and non-experts) were more familiar and experienced with art (and therefore had more information to form their evaluation) compared to participants in previous research that show an ingroup bias in art evaluation. Moreover, eastern artworks are also often considered more “exotic”, a symbol of splendour and opulence, compared to their western counterparts^82^. This could in part explain the bias toward Indian paintings in general found in the current work. Overall, however, the absence of an ingroup bias in Experiment 1 suggests that individuals share aesthetic preferences and experiences when evaluating artworks belonging to different cultures, and are not reliably biased by ingroup favouritism when viewing paintings.

In contrast to findings from Experiment 1, but in line with our hypotheses, in Experiment 2, we found an ingroup bias such that Indian participants preferred Bharatanatyam more than classical ballet, while Western participants preferred ballet over Bharatanatyam. This ingroup bias was modulated by dance expertise. Consistent with our preregistered hypotheses, non-experts showed an ingroup bias in the predicted direction (ingroup favouritism), while this bias was absent among expert participants. This modulation by expertise, however, was only present among our Western participant sample, and did not emerge among the Indian participants. The modulation of expertise in Experiment 2 may be explained by the uncertainty-identity hypothesis where national or cultural identity may be used as a heuristic to evaluate art when one is uncertain about one’s opinions due to a lack of experience or familiarity. Therefore, experts do not show an ingroup bias as they do not need to use an additional heuristic to base their evaluations on, whereas non-experts identify with a group and show ingroup favouritism to resolve their uncertainty^80^.

Similar to the first research question, while we expected the modulation of expertise to manifest similarly across cultures, we only found expertise modulation among our Western participant sample. In order words, both dance naïve and dance expert participants among our Indian participant sample preferred Bharatanatyam to ballet. No difference emerged in the ingroup bias between the two expertise groups. If we assume that non-experts identify with a group and show ingroup favouritism to resolve their identity, a lack of modulation by expertise in our Indian participants suggests that our Indian experts may continue to use an identity heuristic to evaluate artworks, and therefore prefer Bharatanatyam over ballet. Whether this is because of more experience in Bharatanatyam and/or lower experience with ballet is a question for future research. Another possible but less likely explanation for the discrepancy between cultures relates to the dancer featured in the stimuli we created for the current study. Our dancer (who is trained in both Bharatanatyam and ballet dance styles) wore Indian clothing (a *kurta* and leggings) but is of western origin. It is possible that even at this very subtle level, ingroup judgement by participants of Indian or Western origin might have been reinforced to different degrees. Future research should investigate this possibility with multiple dancers.

An important point of consideration is the multi-dimensional nature of aesthetic ratings. For instance, how “beauty” is defined may differ between cultures, and liking something because one feels a sense of national/cultural identity may be different to liking something that feels exotic. That is, even with similar Likert ratings for liking, the aesthetic experience of Indians when liking an Indian painting might be different to the aesthetic experience of Western participants when liking an Indian painting. A considerable but important challenge for future investigations into multicultural aesthetic experiences will be to tease apart different kinds of aesthetic experiences by using indicators that overcome limitations of the Likert-type scales used in the current work.

The modulation of the ingroup bias by dance expertise has important implications for the field of social psychology more generally. As we become increasingly more aware of ingroup biases and prejudices, attempts are being made to counter them. This is especially the case in professional settings and areas where such biases may lead to overt discrimination and poor outcomes in healthcare, law enforcement, education and additional contexts (see^83^ for a review). The modulation by dance expertise of an ingroup bias that we report here highlights the importance of knowledge-based and educational interventions, training, and exposure to different art styles (or cultures, languages, or practices) to help reduce biases against the less familiar. One exciting avenue for future research could be to investigate whether expertise in arts (or training in arts knowledge, or exposure to art) transfers to other domains and leads to a reduction of ingroup biases in non-art contexts. Given that the arts create a snapshot about a culture and society in a particular point in time, place, and context, multicultural art education holds great potential to reduce stigmatisation of other cultures, groups, positions and worldviews, a position advocated by researchers in previous work on art education (e.g.,^84^), and further supported by the current findings.

It is further important to note that no significant difference between Indian and Western participants emerged among the scores on the painting/dance experience questionnaires across both experiments. Thus, a lack of modulation by expertise in both Experiments 1 and 2 in the Indian context cannot be explained by a difference in expertise between the two cultures.

## Conclusion

More than a decade ago, in a seminal paper titled “The Weirdest People in the World?”, Heinrich and colleagues highlighted the variability in populations across the world in major domains that are associated with fundamental aspects of motivation, behaviour, and psychology^85^. Samples drawn from Northern America and Western Europe (which is the case for most previous research in empirical aesthetics) are hardly reflective of the entire population and cannot be used to justify that a behavioural phenomenon is universal. The current cross-cultural investigation thus begins to illuminate which features and aspects of aesthetic experience endure across culture (in this case, primarily Western European and Indian cultures). Across these two cultures, we found a preference for representational fine arts and abstract performing arts. We also found that ingroup biases emerged between cultures when evaluating the performing arts but not the fine arts. Both the preference for representational fine art and an ingroup bias for dance were modulated by prior relevant artistic expertise only for Western participants. Thus, the evidence reported here highlights that both cultural specifics and anthropological universals of human art creation and appreciation exist, and are aesthetically relevant across the fine and performing arts, as well as Indian and Western cultures. In an increasingly divided and fractured world where many seek to fortify boundaries based on culture, race, and country, our results point to the potential of art and art experience as a unique equaliser that can bind people together.

## Supplementary Information

Supplementary Information.
