# Supplementary material for: The role of expertise and culture in visual art appreciation
Source: Sci Rep. 2022 Jun 23;12:10666. doi: 10.1038/s41598-022-14128-7 (PMC9219380; doi:10.1038/s41598-022-14128-7)
Supplement: Supplementary file 1 — Supplementary Information. [file 41598_2022_14128_MOESM1_ESM.docx]

**A Unifying Model of Visual Art Appreciation:**

**The Role of Expertise and Culture – SUPPLEMENTARY MATERIAL**

All data, code, and stimuli used can be found on the Open Science Framework - https://osf.io/vtw54/.

**EXPERIMENT 1.**

1. **Stimuli Creation**

A first pool of 120 images of paintings were chosen randomly from online databases: 60 by Indian painters, 60 by Western painters, out of which 30 each were abstract and representational paintings. Paintings were categorized by the experimenters as either ‘abstract’ or ‘representational’ depending on the content of the painting. That is, paintings depicting representational or figurative content (such as still life or landscapes) were categorized as ‘representational’ and paintings depicting content that was abstract (or not representative of anything concrete or figurative) were classified as ‘abstract.’ The final set of stimuli were chosen as follows: first, an independent sample of participants (N=21, 13 females, Mean_age_=29.14, SD_age_=6.73) rated the first pool of 120 images for abstract and representational paintings on familiarity, complexity, and evocativeness. Participants were not professional artists. Ten participants had no experience with art, while 11 participants had taken some art classes. For more details on this, please refer to the rawdata for pilot 1 in the OSF repository. They also answered two forced choice questions – whether they thought the painting was abstract or representational, and whether they thought the painting was by an Indian painter or Western painter. For the ratings of familiarity, evocativeness, and complexity, a likert scale from 1 (low/not at all) to 5 (high/extremely) was used.

Next, all paintings with a familiarity>2 were excluded. This was because we wanted participants to be unfamiliar with the paintings. This resulted in 85 paintings. To choose the final set of paintings, we used Macros in Microsoft Excel (code is available on OSF: https://osf.io/vtw54/). Out of the 85 paintings, we identified paintings (with the requirement that there should be a minimum 8 paintings per condition: Indian Abstract, Western Abstract, Indian Representational, Western Representational) that matched most closely on their average ratings of familiarity, evocativeness, and complexity. This was because we wanted all paintings to be as similar to each other as possible such that any differences between abstract and representational paintings of Indian and Western cultures could not be accounted for by these subjective variables (we also controlled for these variables in the main analyses). The final stimulus set thus consisted of 35 paintings – 10 Indian abstract, 8 Western abstract, 8 Indian representational, and 9 Western representational paintings. The paintings were resized to 500 x 500 pixels, and matched for mean luminance using the SHINE toolbox in MATLAB (Willenbocket et al., 2010). Therefore, the final stimulus set was closely matched across all four categories of paintings (Indian Abstract, Indian Representational, Western Abstract, Western Representational) on variables of luminance, familiarity, complexity, and evocativeness. Average ratings on familiarity, evocativeness, complexity, and luminance are provided in Table S1. The final stimulus set of 35 paintings is available on the OSF (https://osf.io/vtw54/).

*Table S1*. Mean ratings on familiarity, evocativeness, and complexity (for Pilot 1) for the selected 35 paintings that are used in the main experiment.

|  | Mean Familiarity | Mean Complexity | Mean Evocativeness | Mean HSV (Luminance) |
| --- | --- | --- | --- | --- |
| Indian Abstract | 1.54 | 3.20 | 2.42 | 0.54 |
| Western Abstract | 1.51 | 3.28 | 2.42 | 0.54 |
| Indian Representational | 1.83 | 3.25 | 2.55 | 0.19 |
| Western Representational | 1.87 | 3.34 | 2.53 | 0.19 |

1. **Power Analyses**

We determined the sample size based on a simulation-based power analysis approach using the simr R package (Green & Macleod, 2016). First, we used pilot data (N=22, 14 females, 10 art experts, Mean_age_ = 29.71, SD_age_= 9.86) for beta weight estimation for the following mixed effects model: beauty ~ category*expertise + (1|subject) + (1|item). Second, we simulated data by extending along the sample size, i.e., as a function of different sample sizes (see Figure S1). Our main focus was the interaction between the category of the painting and the art expertise of participants. We used the test “fcompare” i.e. we determined the sample size required for us to detect a significant category by art expertise interaction in the model compared to a simpler model of just the main effects and random effects of subject and item. The power analysis suggested that we required a sample size of 50 participants (25 experts and 25 non-experts) with 35 items to have >90% power to detect a significant category*expertise interaction. We therefore aimed to stop data collection when over 100 participants finished the entire survey, with an aim to recruit approximately 50 Indian participants and 50 Western participants with 25 experts and 25 non-experts within each culture.


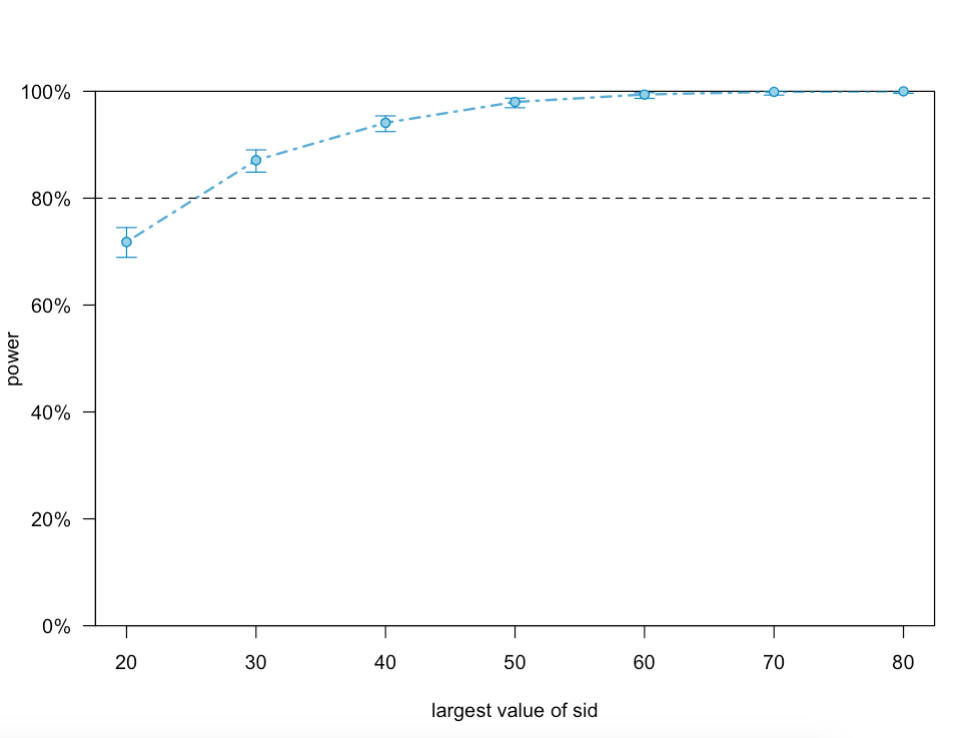


*Figure S1.* Power for the category*art expertise interaction (on the y-axis) as a function of sample size (on the x-axis) for beauty ratings.

1. **Main Experiment**

A] Participant sample geographic distribution.

*Table S2. Geographic distribution of the participant sample for the main experiment (N=92).*

| Geographic region | N |
| --- | --- |
| India | 44 |
| Nepal* | 1 |
| Czech Republic | 1 |
| England | 34 |
| France | 2 |
| Germany | 1 |
| Jordan | 1 |
| Netherlands | 1 |
| Poland | 2 |
| Romania | 1 |
| Russia | 1 |
| Slovenia | 1 |
| United States of America | 1 |

*Participant from Nepal was included in the Indian group because of cultural similarity. Results are similar with and without the participant being included in the analysis.

B] Mean ratings for each category and source of painting across culture and expertise.

*Table S3.* Mean ratings for each category and source of painting on familiarity (mean_fam), complexity (mean_com), evocativeness (mean_evo), technical competency (mean_techcomp), beauty (mean_beauty), liking (mean_liking), and abstractness (mean_abs) across culture and expertise.


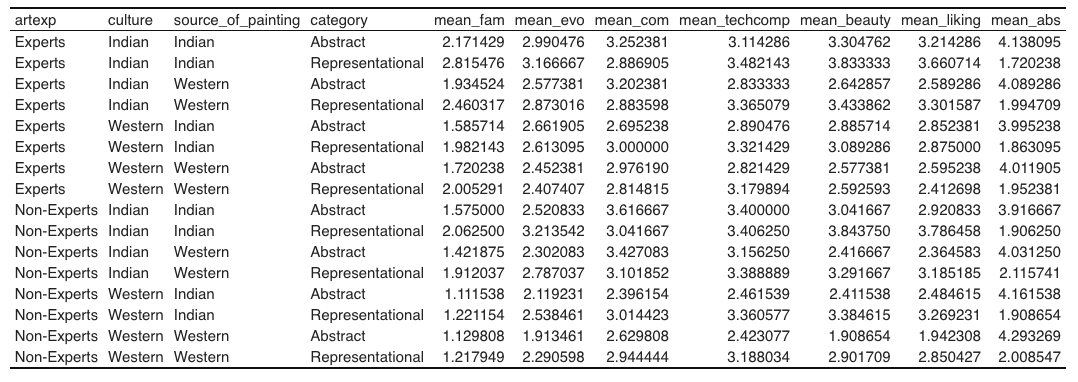


*Figure S2. Mean ratings of familiarity across category (abstract, representational), culture (Indian/Western), source of painting (Indian, Western), and art expertise (expert, nonexpert).*

*
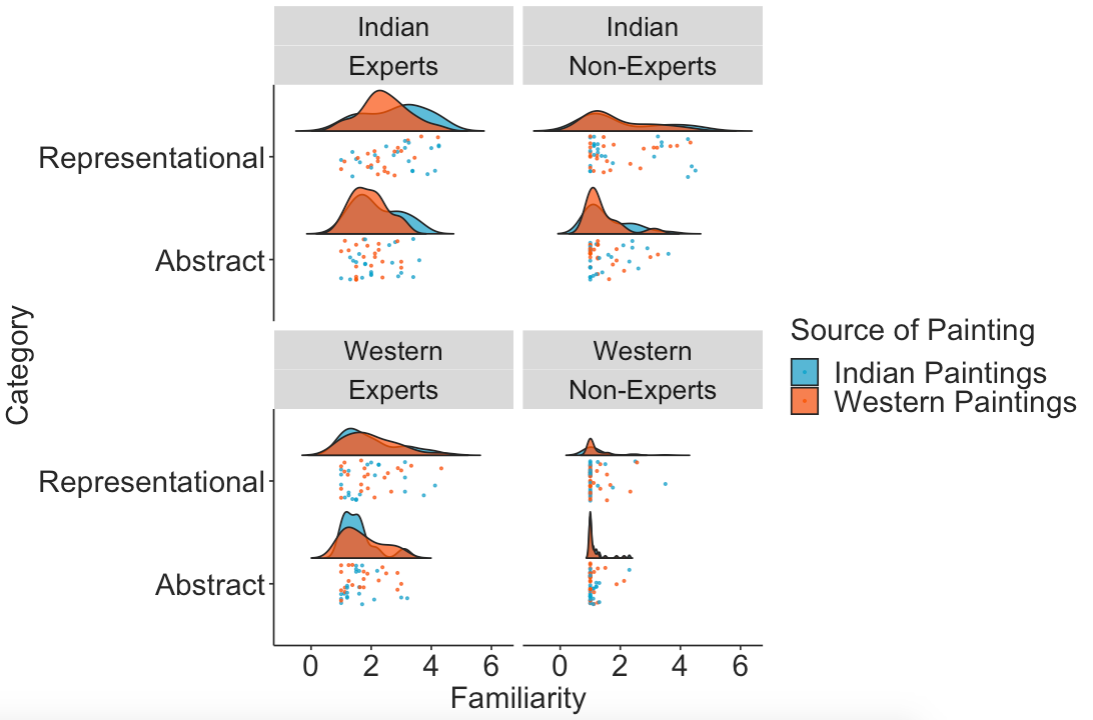
*

*Figure S3. Mean ratings of complexity across category (abstract, representational), culture (Indian/Western), source of painting (Indian, Western), and art expertise (expert, nonexpert).*

*
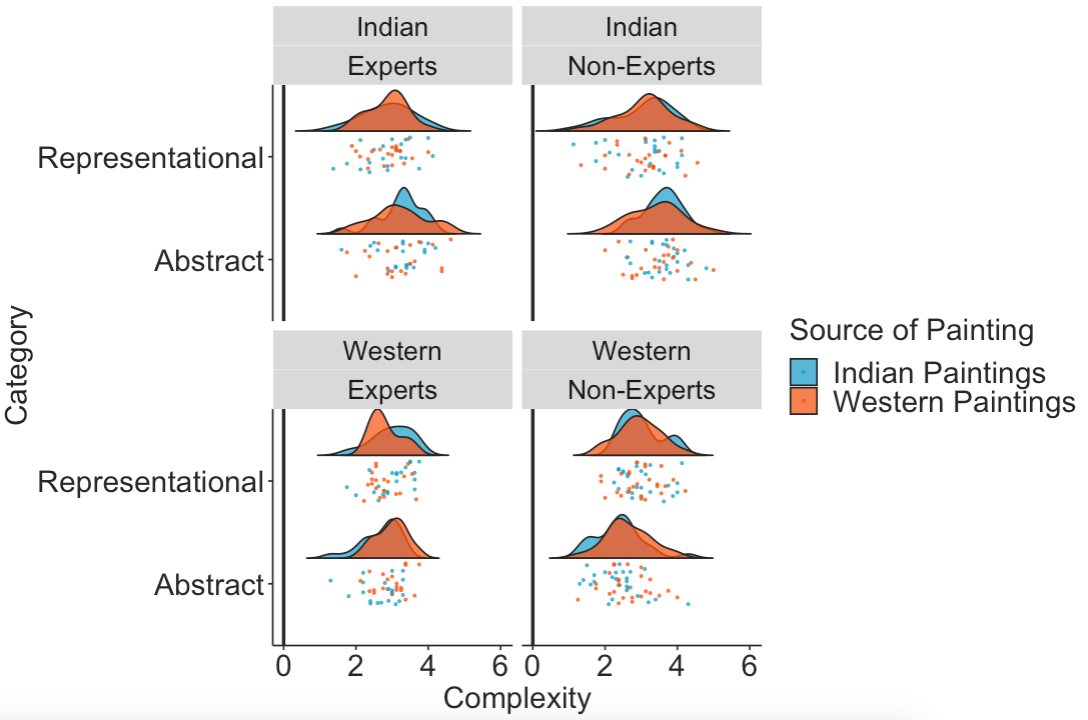
*

*Figure S4. Mean ratings of evocativeness across category (abstract, representational), culture (Indian/Western), source of painting (Indian, Western), and art expertise (expert, nonexpert).*

*
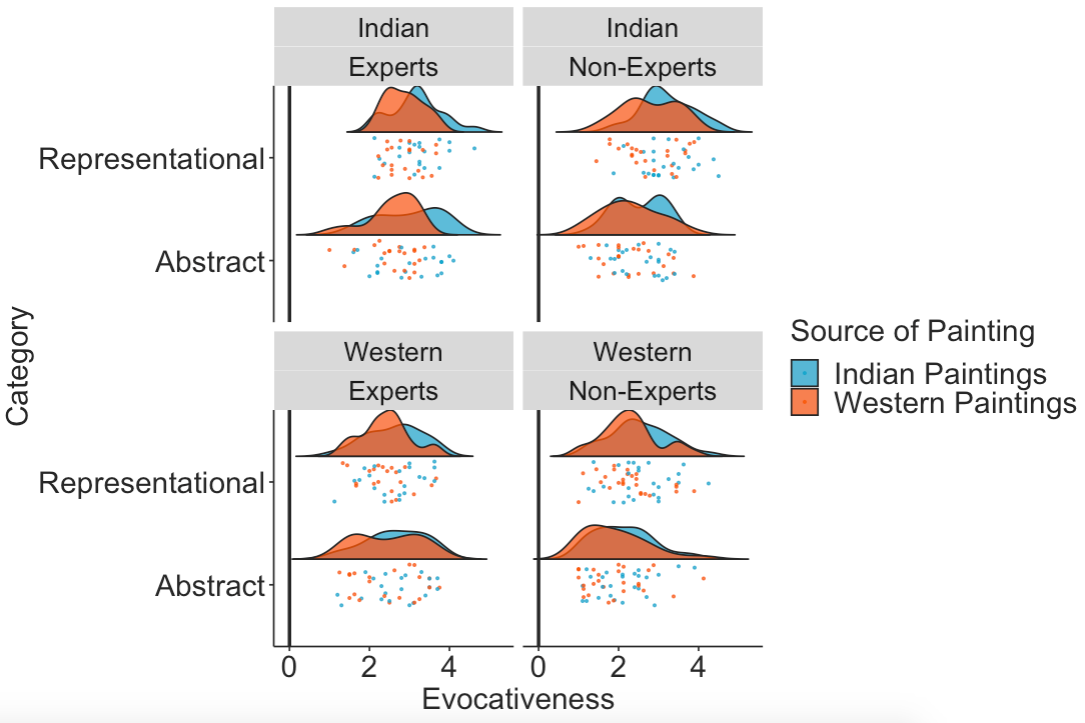
*

*Figure S5. Mean ratings of technical competency across category (abstract, representational), culture (Indian/Western), source of painting (Indian, Western), and art expertise (expert, nonexpert).*

*
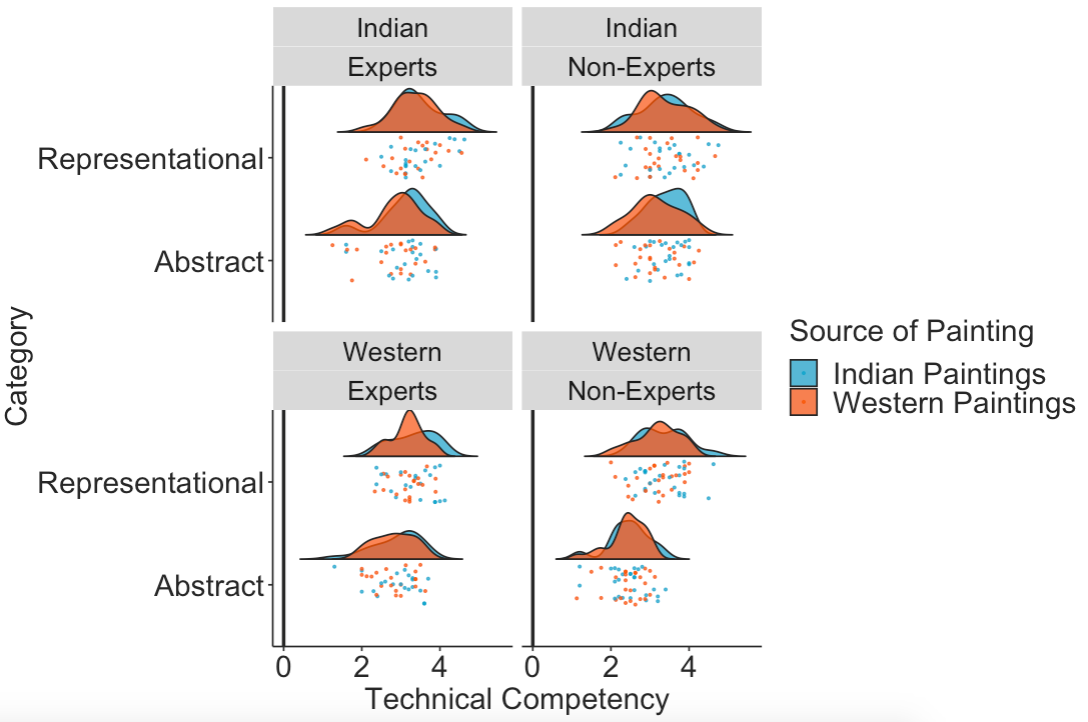
*

*Figure S6. Mean ratings of beauty across category (abstract, representational), culture (Indian/Western), source of painting (Indian, Western), and art expertise (expert, nonexpert).*

*
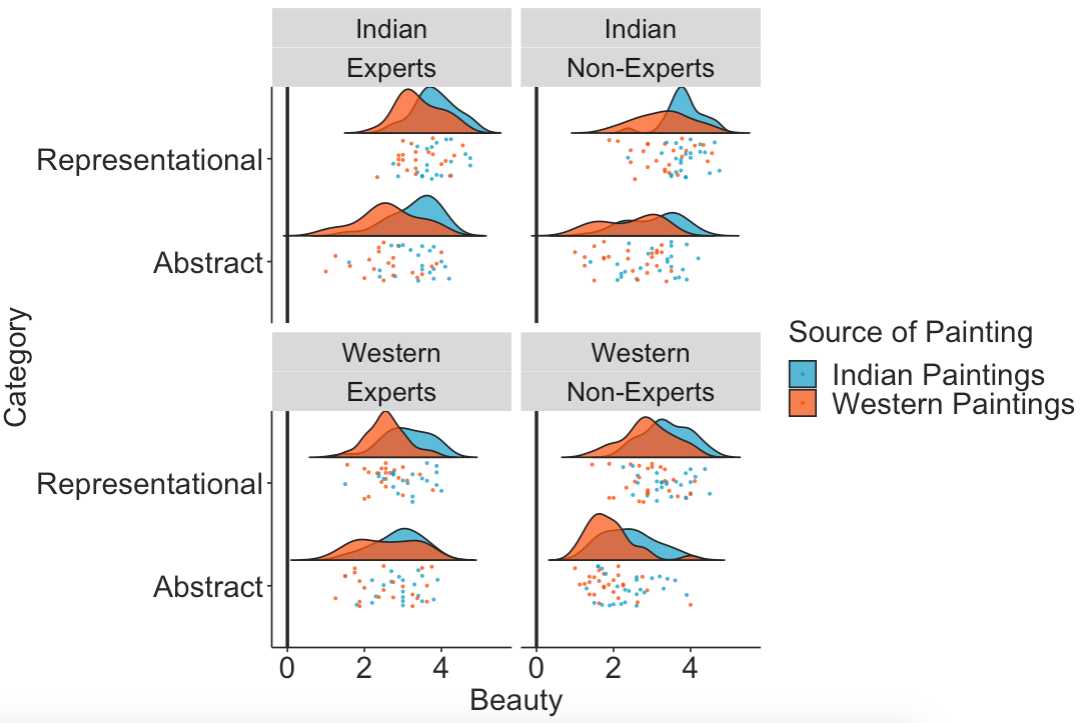
*

*Figure S7. Mean ratings of liking across category (abstract, representational), culture (Indian/Western), source of painting (Indian, Western), and art expertise (expert, nonexpert).*

*
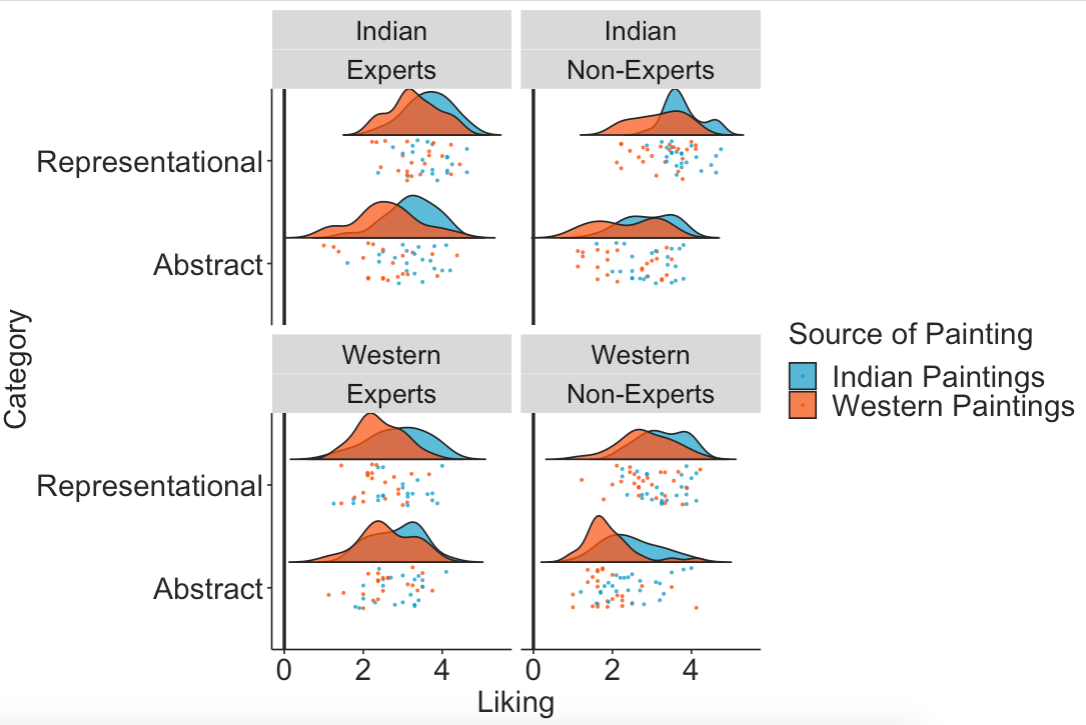
*

*Figure S8. Mean ratings of abstractness across category (abstract, representational), culture (Indian/Western), source of painting (Indian, Western), and art expertise (expert, nonexpert).*

*
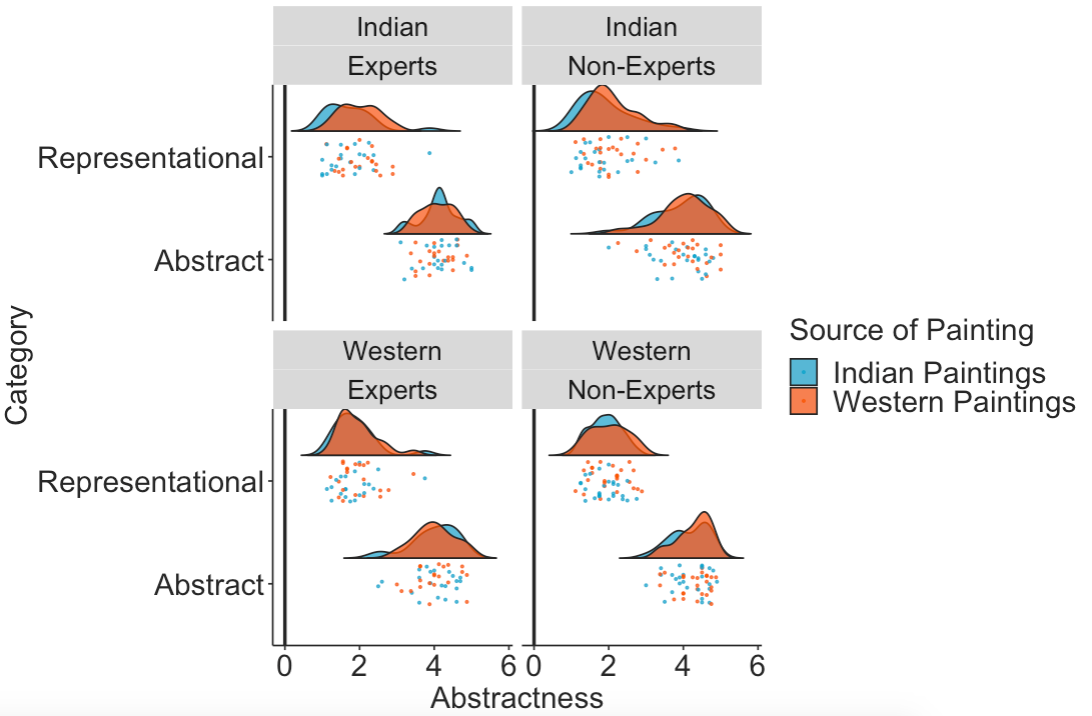
*

C] Cumulative link mixed models statistics for beauty and liking ratings for the main model and the model with subjective variables.

*Table S4. RQ1: Cumulative link mixed model: category*expertise*culture interaction.*

*NB: the 1/2 2/3 3/4 and 4/5 terms in this (and following tables) refer to the likert scale ratings of 1 to 5 (for instance, 1/2 refers to the rating ‘1’ compared to rating ‘2’ as these ratings are entered into the model as a factor variable (as opposed to a numeric variable which is the norm when doing linear mixed effects models).*


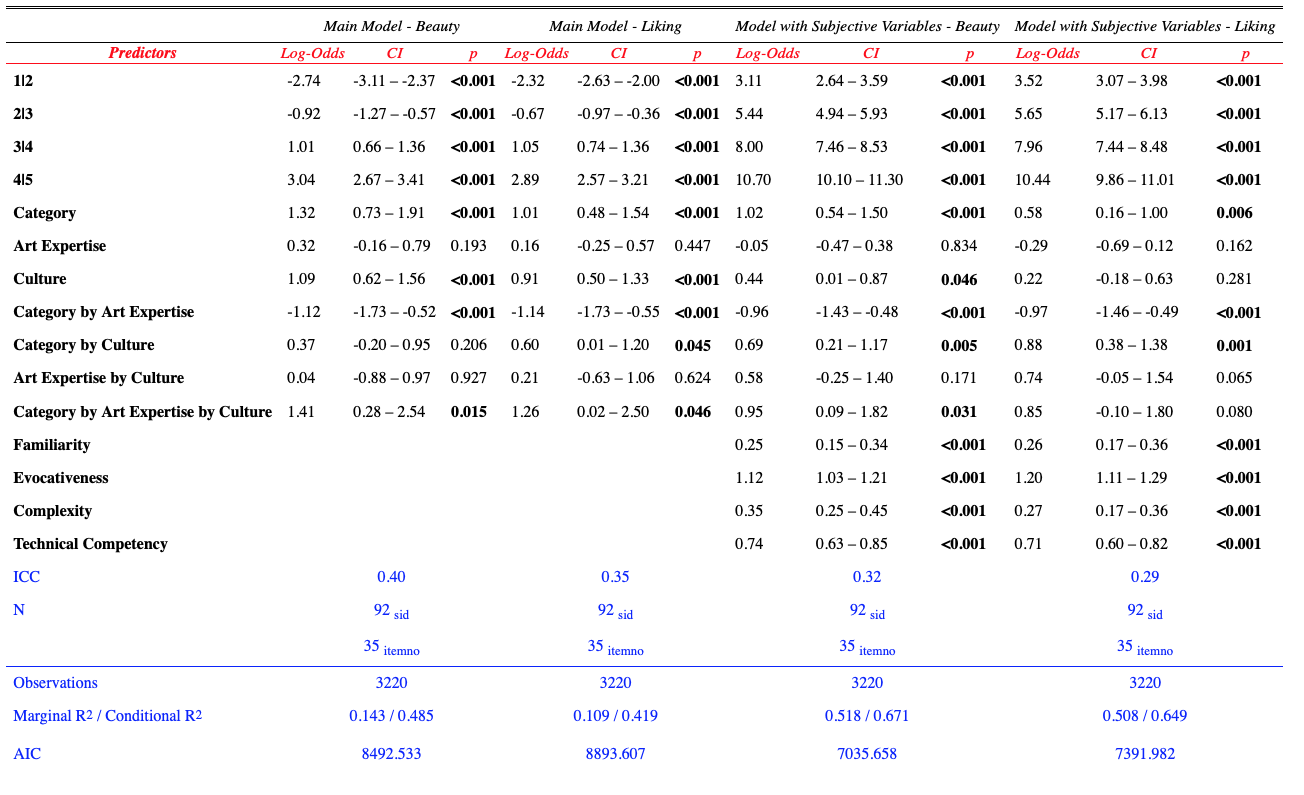


*Table S5. RQ2: Cumulative link mixed model: source of painting*expertise*culture interaction.*

*
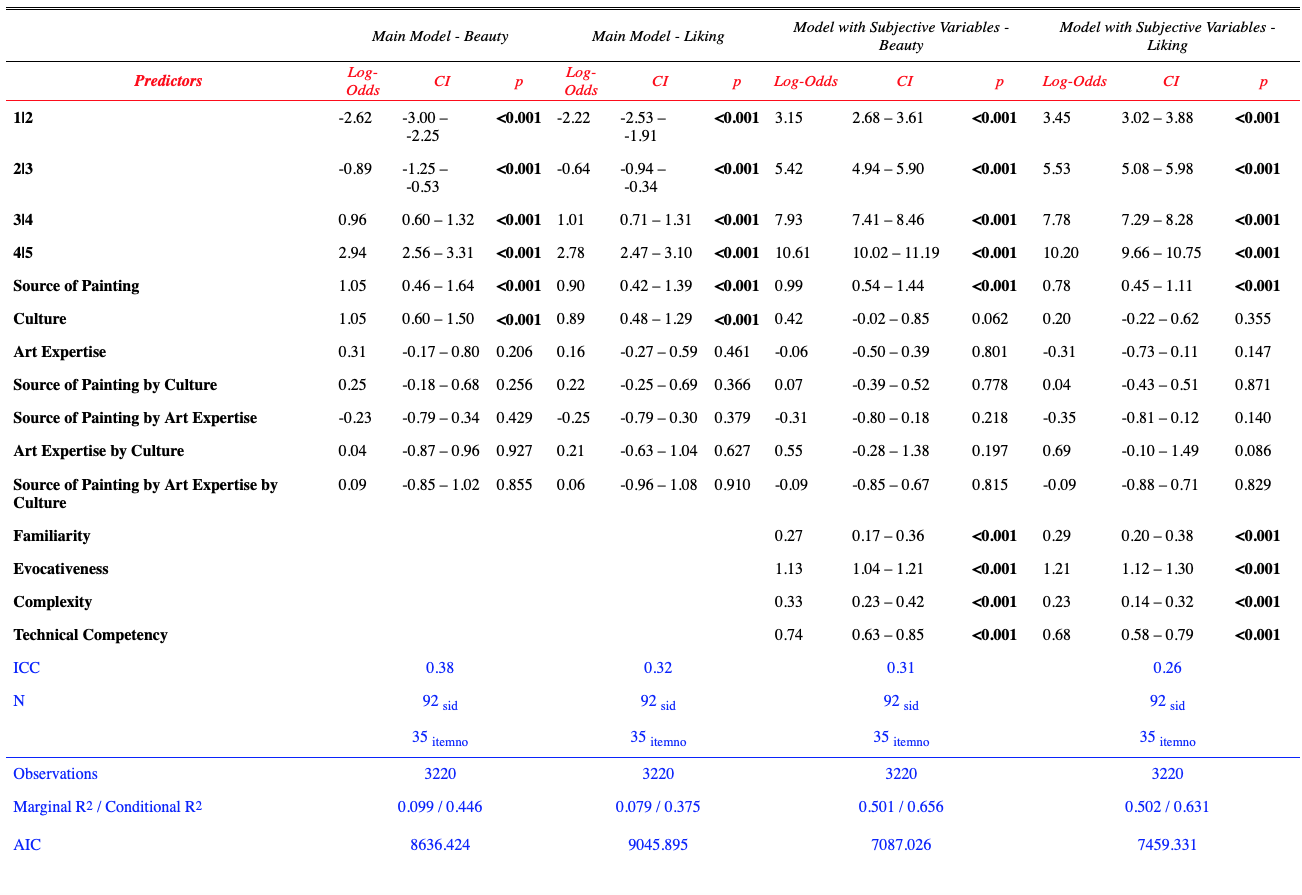
*

*Table S6. RQ2: Cumulative link mixed model: source of painting*expertise*culture interaction, when source of painting is categorized by the participants themselves.*

*
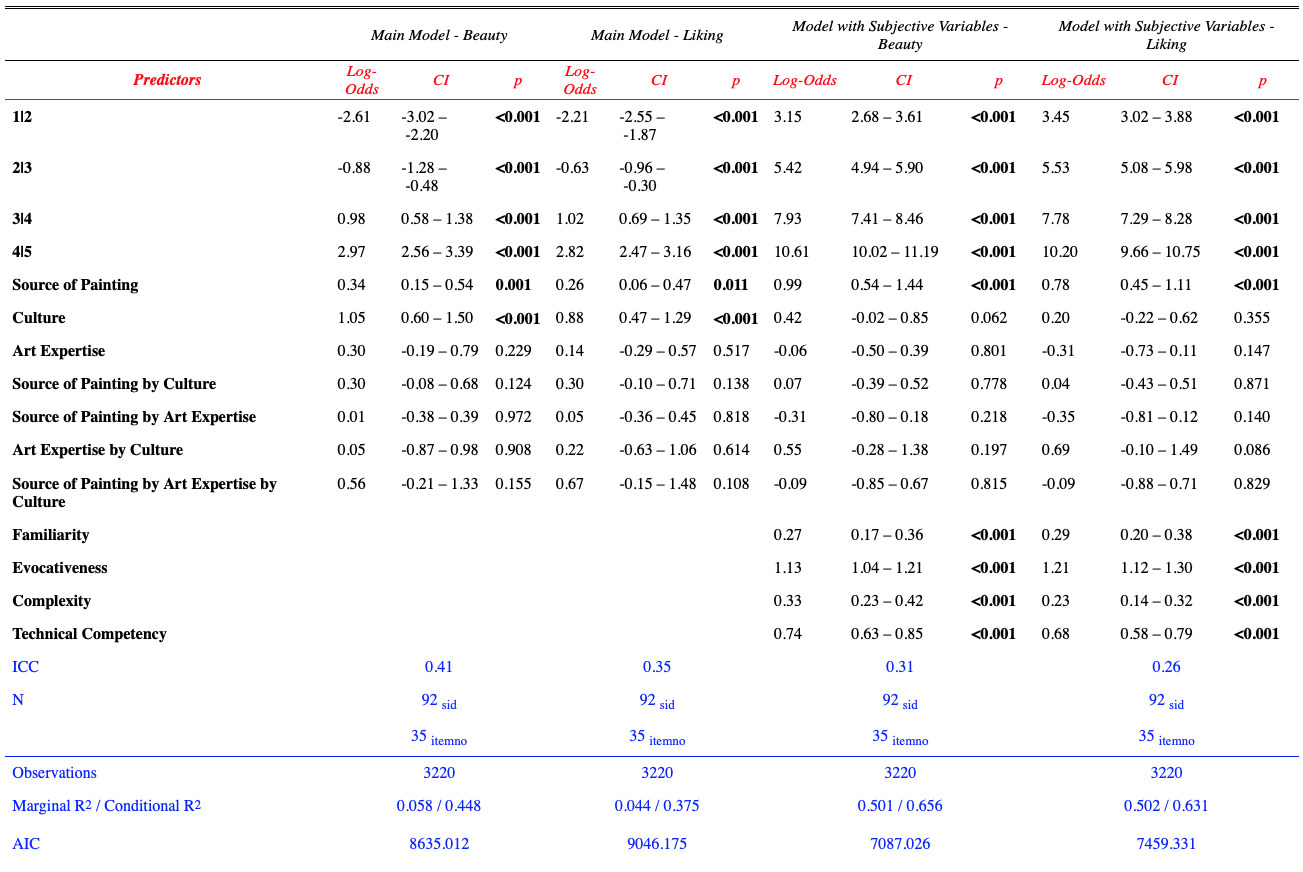
*

*Table S7. RQ1. Cumulative link model statistics for category*art expertise for Indian and Western participants separately.*

*
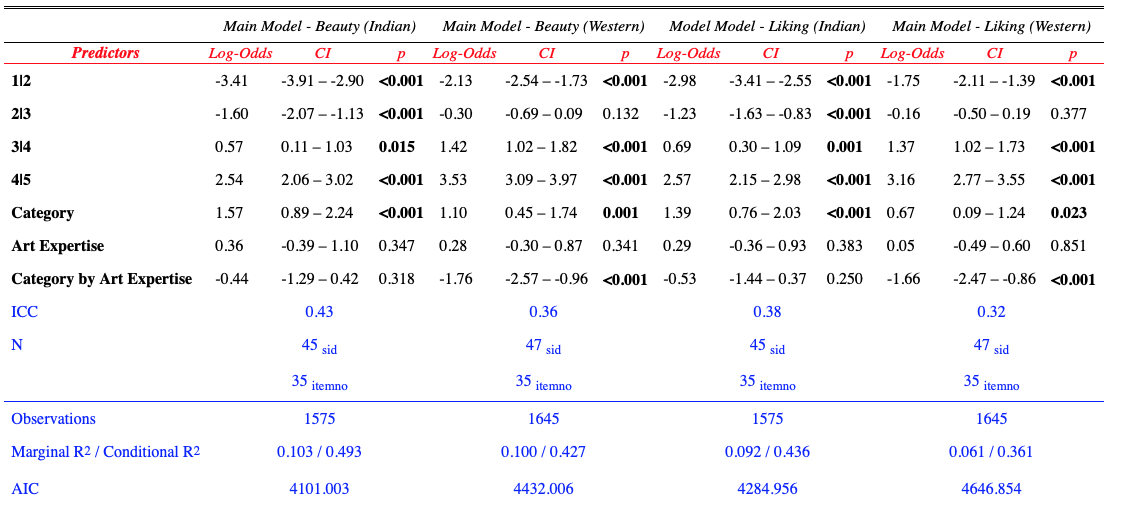
*

*Table S8. RQ1. Means and SDs of beauty and liking ratings for the category x art expertise x culture interaction.*

| Expertise | Culture | Category | Mean_Beauty | Mean_Liking | SD_Beauty | SD_Liking |
| --- | --- | --- | --- | --- | --- | --- |
| Experts | Indian | Abstract | 1.97 | 2.90 | 0.80 | 0.81 |
| Experts | Indian | Representational | 3.63 | 3.48 | 0.59 | 0.62 |
| Experts | Western | Abstract | 2.73 | 2.72 | 0.72 | 0.66 |
| Experts | Western | Representational | 2.84 | 2.64 | 0.62 | 0.70 |
| Non-Experts | Indian | Abstract | 2.73 | 2.64 | 0.82 | 0.77 |
| Non-Experts | Indian | Representational | 3.57 | 3.48 | 0.67 | 0.64 |
| Non-Experts | Western | Abstract | 2.16 | 2.21 | 0.71 | 0.75 |
| Non-Experts | Western | Representational | 3.14 | 3.06 | 0.68 | 0.67 |

**EXPERIMENT 2.**

1. **Stimuli Creation**

We invited a professional dancer trained in classical ballet and Bharatanatyam, Sophia Salingaros (https://www.instagram.com/sophiasalingaros/), to record Bharatanatyam and ballet dance videos. We recorded both ballet and Bharatanatyam dance videos featuring movement sequences that either were intended to represent something in the external world (e.g. humans, animals, birds, nature, etc.) or not represent anything in particular (pure dance or non-representational abstract dance). The videos were edited in iMovie into 10-12 second clips, and the first 0.5 seconds of the video faded in, and the last 0.5 seconds faded out to a black screen. An independent sample of participants (N=13, 8 females, Mean_age_=28.85, SD_age_=8.93) rated the first pool of stimuli of abstract and representational dance videos on familiarity, complexity, and evocativeness. For the ratings of familiarity, evocativeness, and complexity, a likert scale from 1 (low/not at all) to 5 (high/extremely) was used.

Out of a total of 91 videos (46 Bharatanatyam/Indian dance videos, out of which 18 were abstract, and 45 ballet/Western dance videos, out of which 25 were abstract), to choose the final set of dance videos, we used Macros in Microsoft Excel (code is available on OSF: <https://osf.io/vtw54/>). Out of the 91 dance videos, two videos were extracted as they were not accurately categorized as abstract or representational by more than 50% of participants. Out of the remaining 89 videos, we identified dance videos (with the requirement that there should be a minimum 7 videos per condition: Indian Abstract, Western Abstract, Indian Representational, Western Representational) that matched most closely on their average ratings of familiarity, evocativeness, and complexity. This was because we wanted all paintings to be as similar to each other as possible such that any differences between abstract and representational paintings of Indian and Western cultures could not be accounted for by these subjective variables (we also controlled for these variables in the main analyses).

The final stimuli consisted of 38 dance videos – 11 Indian abstract, 8 Western abstract, 11 Indian representational, and 8 Western representational dances, matched on the variables of familiarity, complexity, and evocativeness. Average ratings on familiarity, evocativeness, complexity, are provided in Table S9. The final stimulus set of 38 videos is available on the OSF (https://osf.io/vtw54/).

*Table S9*. Mean ratings (for Pilot 1) on familiarity, evocativeness, and complexity for the selected 38 dance videos that are used in the main experiment.

|  | Mean Familiarity | Mean Complexity | Mean Evocativeness |
| --- | --- | --- | --- |
| Ballet Abstract | 2.50 | 2.70 | 2.60 |
| Bharatanatyam Abstract | 2.56 | 2.79 | 2.91 |
| Ballet Representational | 2.20 | 2.66 | 2.90 |
| Bharatanatyam Representational | 2.34 | 2.44 | 2.85 |

1. **Power Analyses**

We determined the sample size based on a simulation-based power analysis approach using the simr R package (Green & Macleod, 2016). First, we used pilot data (N=21, 17 females, 12 dance experts, Mean_age_ = 29.71, SD_age_= 9.86) for beta weight estimation for the following mixed effects model: beauty ~ category*expertise + (1|subject) + (1|item). Second, we simulated data by extending along the sample size, i.e., as a function of different sample sizes (see Figure S9). Our focus was the interaction between the category of the painting and the art expertise of participants. We used the test “fcompare” i.e. we determined the sample size required for us to detect a significant category by art expertise interaction in the model compared to a simpler model of just the main effects and random effects of subject and item. The power analysis suggested that we required a sample size of 50 participants (25 experts and 25 non-experts) with 38 items to have >80% power to detect a significant category*expertise interaction. We therefore aimed to stop data collection when over 100 participants finished the entire survey, with an aim to recruit approximately 50 Indian participants and 50 Western participants with 25 experts and 25 non-experts within each culture.

*
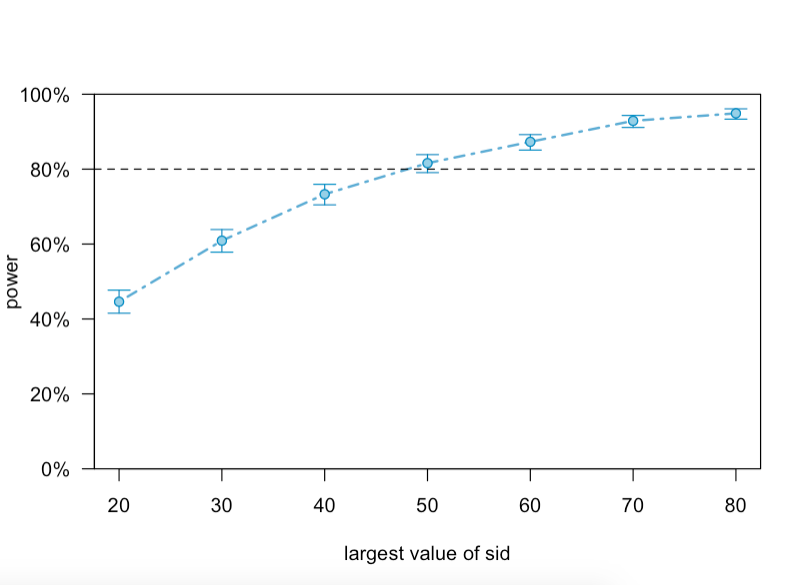
*

*Figure S9.* Power for the category*art expertise interaction (on the y-axis) as a function of sample size (on the x-axis) for beauty ratings.

1. **Main Experiment**

A] Participant sample geographic distribution.

*Table S10. Geographic distribution of the participant sample for the main experiment (N=90).*

| Geographic region | N |
| --- | --- |
| India | 48 |
| Pakistan* | 1 |
| China* | 1 |
| United Kingdom | 29 |
| United States of America | 3 |
| Canada | 2 |
| Argentina | 1 |
| Brazil | 1 |
| Greece | 1 |
| Ireland | 1 |
| Italy | 1 |
| Slovenia | 1 |
| Slovakia | 1 |
| Spain | 1 |

*2 participants from Pakistan and China were excluded from the analyses. Results are similar with and without the participants being included in the analysis.

B] Mean ratings for each category and dance style across culture and expertise.

*Table S11.* Mean ratings for each category and dance style on familiarity (mean_fam), complexity (mean_com), evocativeness (mean_evo), technical competency (mean_techcomp), reproducibility (mean_reprod), beauty (mean_beauty), liking (mean_liking), abstractness (mean_abs) and enjoyability (mean_enjoy) across culture and expertise.

| Expertise | Culture | Dance Style | Category | Mean_fam | Mean_evo | Mean_com | Mean_techcomp | Mean_beauty | Mean_liking | Mean_abs | Mean_reprod | Mean_enjoy |
| --- | --- | --- | --- | --- | --- | --- | --- | --- | --- | --- | --- | --- |
| Experts | Indian | Bharatanatyam | Abstract | 4.316 | 2.727 | 2.383 | 3.430 | 3.719 | 3.679842 | 3.083 | 1.861 | 3.652 |
| Experts | Indian | Bharatanatyam | Representational | 3.932 | 3.335 | 2.154 | 3.031 | 3.660 | 3.501976 | 2.221 | 1.857 | 3.498 |
| Experts | Indian | Ballet | Abstract | 2.364 | 2.657 | 3.168 | 3.478 | 3.581 | 3.423913 | 3.532 | 3.336 | 3.375 |
| Experts | Indian | Ballet | Representational | 2.657 | 3.054 | 2.880 | 3.201 | 3.608 | 3.391304 | 2.711 | 2.809 | 3.451 |
| Experts | Western | Bharatanatyam | Abstract | 1.752 | 2.628 | 3.252 | 3.595 | 3.454 | 3.557851 | 2.780 | 3.078 | 3.508 |
| Experts | Western | Bharatanatyam | Representational | 1.863 | 2.731 | 2.318 | 2.830 | 3.090 | 3.152893 | 2.566 | 2.152 | 3.194 |
| Experts | Western | Ballet | Abstract | 3.954 | 2.176 | 2.426 | 3.00 | 3.238 | 3.142046 | 2.352 | 2.062 | 3.170 |
| Experts | Western | Ballet | Representational | 3.250 | 2.732 | 2.204 | 2.767 | 3.250 | 3.153409 | 2.159 | 1.948 | 3.056 |
| Non-Experts | Indian | Bharatanatyam | Abstract | 3.243 | 2.680 | 3.363 | 3.705 | 3.647 | 3.461818 | 3.520 | 3.363 | 3.440 |
| Non-Experts | Indian | Bharatanatyam | Representational | 2.978 | 3.243 | 2.374 | 2.981 | 3.280 | 3.112 | 2.440 | 2.450 | 3.160 |
| Non-Experts | Indian | Ballet | Abstract | 2.385 | 2.520 | 3.035 | 3.390 | 3.290 | 3.045 | 3.610 | 3.090 | 3.020 |
| Non-Experts | Indian | Ballet | Representational | 2.525 | 3.130 | 2.775 | 3.090 | 3.410 | 3.195 | 2.880 | 2.890 | 3.170 |
| Non-Experts | Western | Bharatanatyam | Abstract | 2.254 | 2.768 | 3.222 | 3.290 | 3.122 | 3.140 | 3.254 | 3.300 | 3.122 |
| Non-Experts | Western | Bharatanatyam | Representational | 1.804 | 2.777 | 2.404 | 2.450 | 2.754 | 2.613 | 2.959 | 2.404 | 2.659 |
| Non-Experts | Western | Ballet | Abstract | 2.900 | 2.987 | 2.706 | 2.937 | 3.556 | 3.356 | 2.987 | 2.868 | 3.281 |
| Non-Experts | Western | Ballet | Representational | 2.475 | 3.043 | 2.500 | 2.681 | 3.350 | 3.118 | 2.918 | 2.531 | 3.012 |

*Figure S10. Mean ratings of familiarity across category (abstract, representational), culture (Indian/Western), source of painting (Indian, Western), and art expertise (expert, nonexpert).*


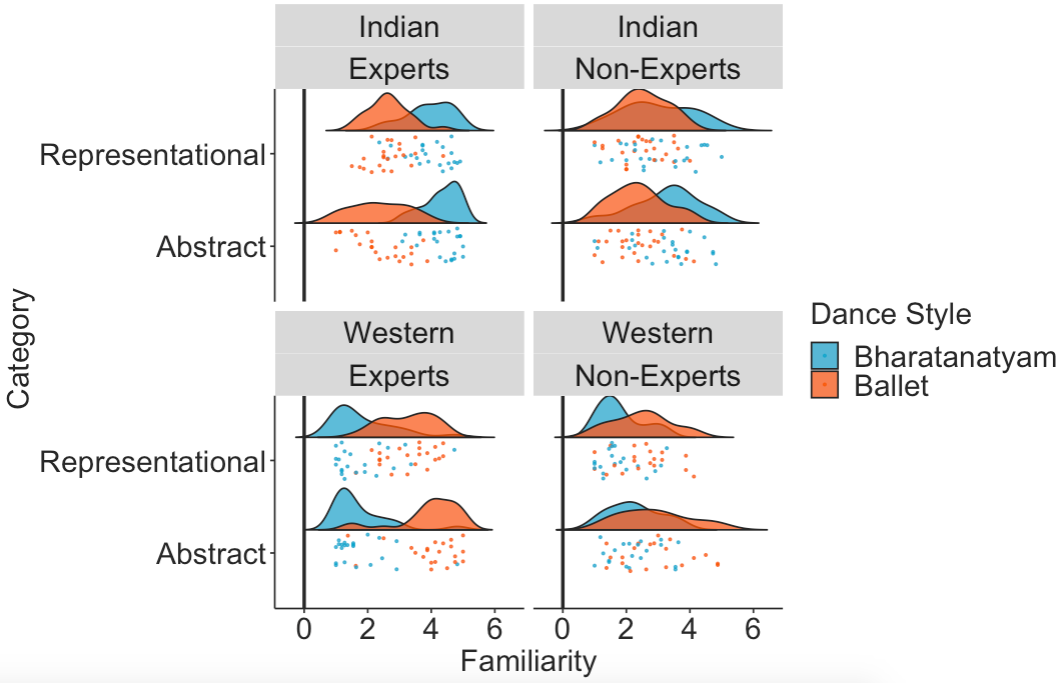


*Figure S11. Mean ratings of evocativeness across category (abstract, representational), culture (Indian/Western), source of painting (Indian, Western), and art expertise (expert, nonexpert).*

*
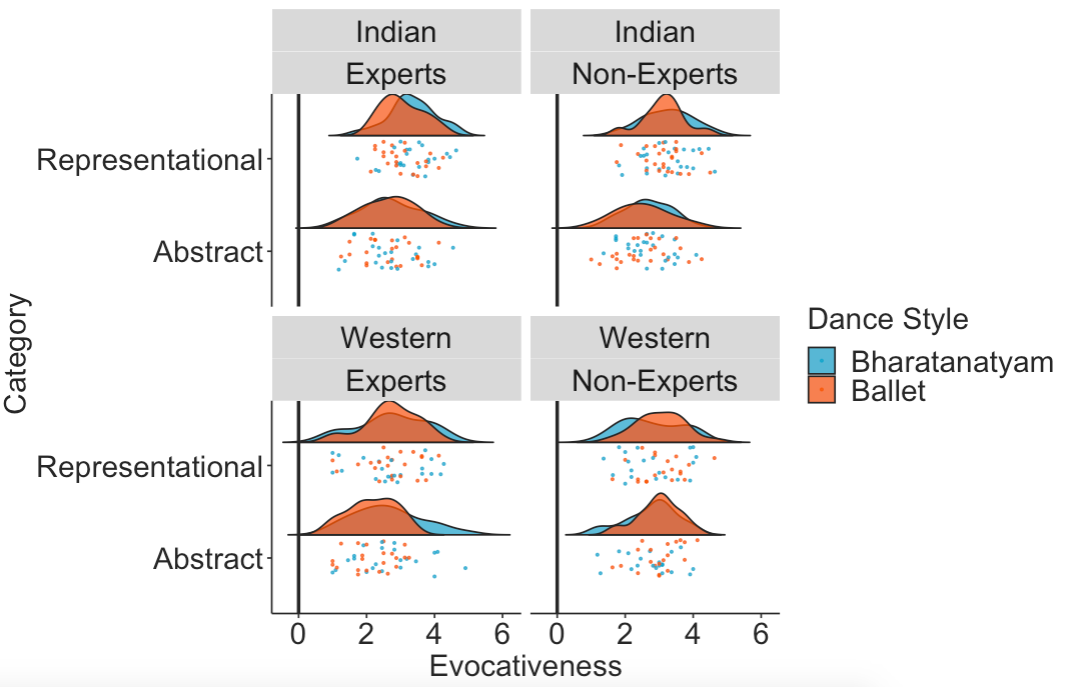
*

*Figure S12. Mean ratings of complexity across category (abstract, representational), culture (Indian/Western), source of painting (Indian, Western), and art expertise (expert, nonexpert).*

*
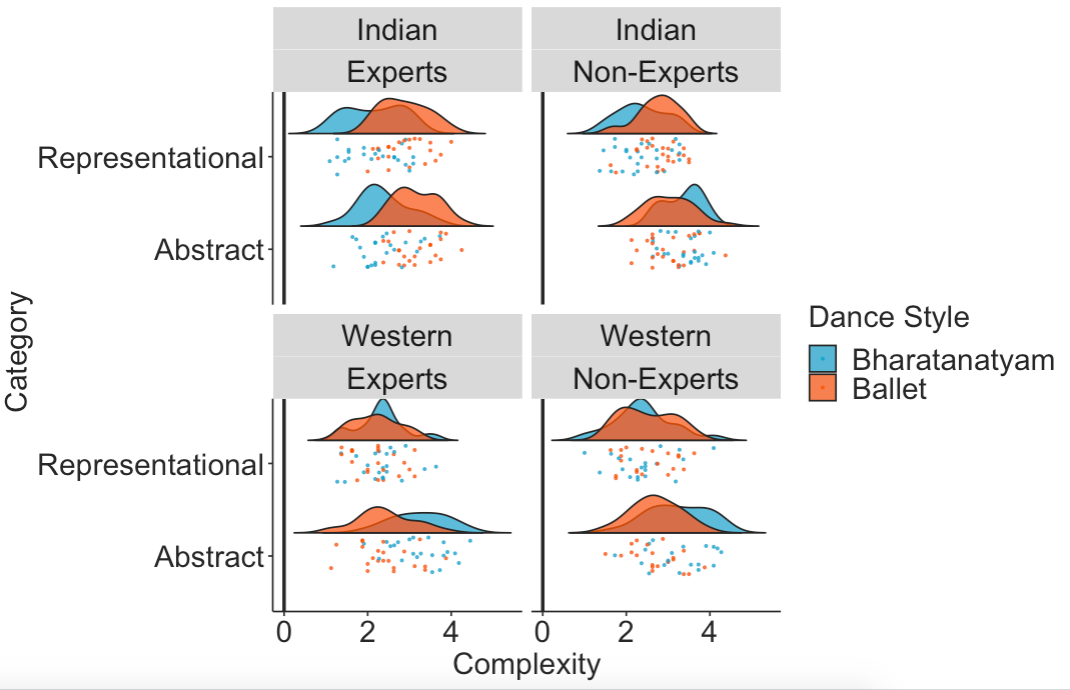
*

*Figure S13. Mean ratings of technical complexity across category (abstract, representational), culture (Indian/Western), source of painting (Indian, Western), and art expertise (expert, nonexpert).*

*
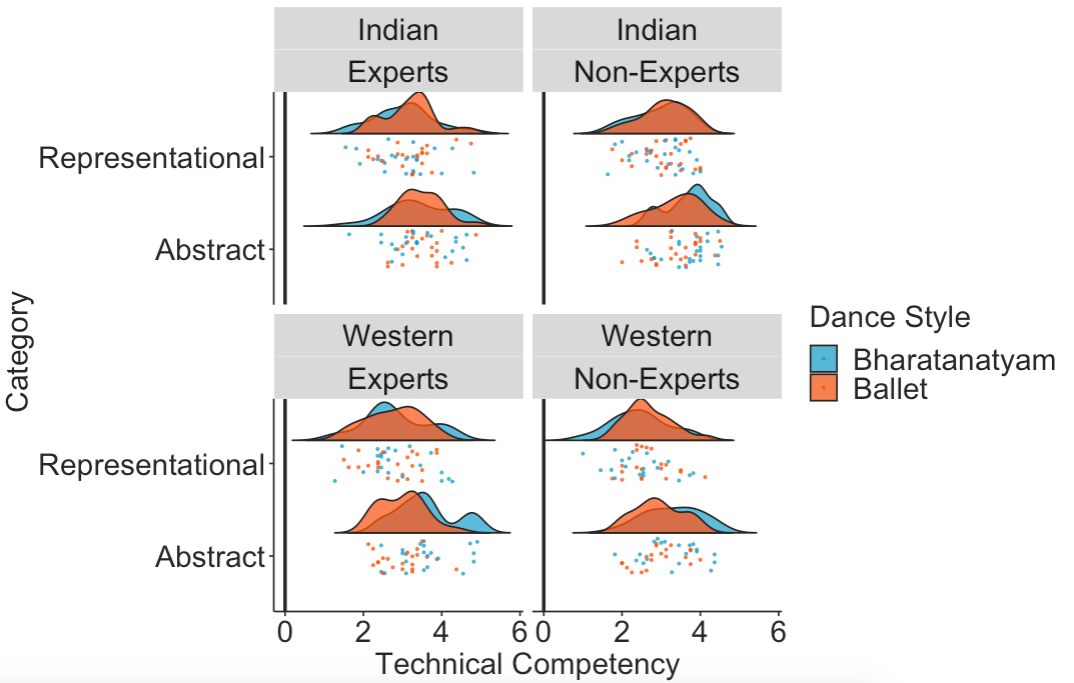
*

*Figure S14. Mean ratings of reproducibility across category (abstract, representational), culture (Indian/Western), source of painting (Indian, Western), and art expertise (expert, nonexpert).*

*
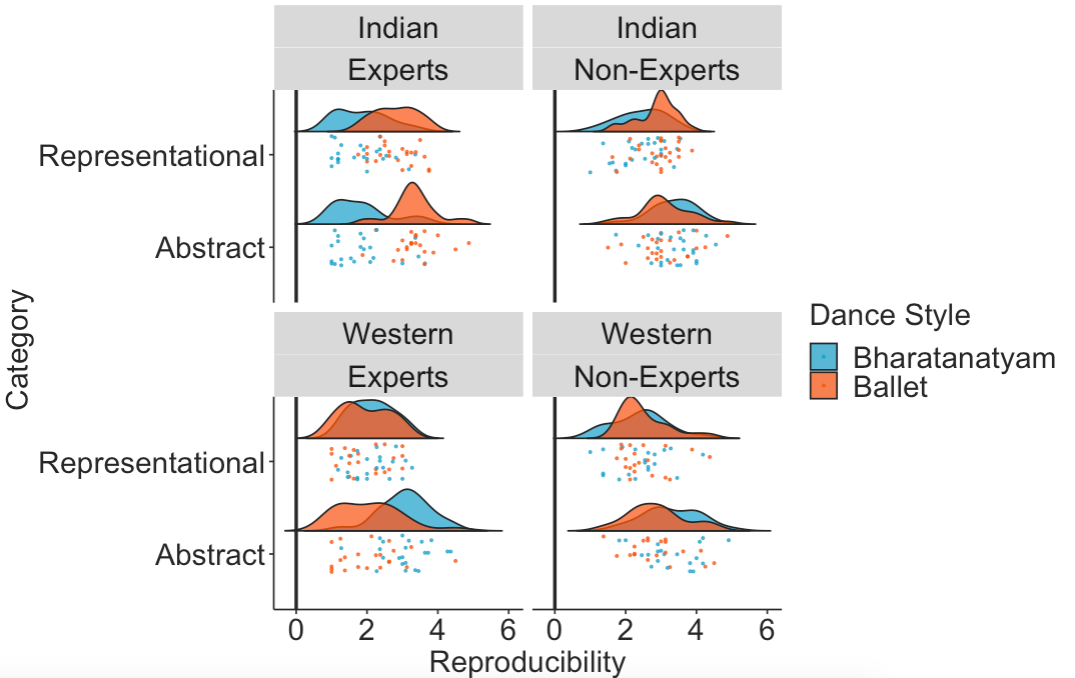
*

*Figure S15. Mean ratings of abstractness across category (abstract, representational), culture (Indian/Western), source of painting (Indian, Western), and art expertise (expert, nonexpert).*

*
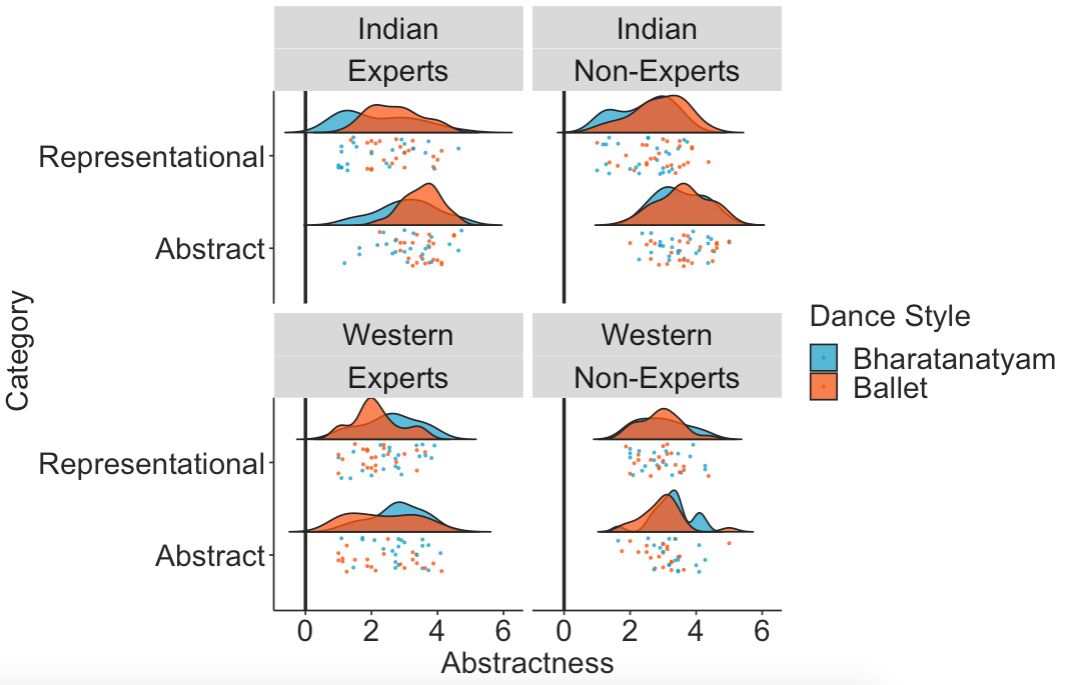
*

*Figure S16. Mean ratings of beauty across category (abstract, representational), culture (Indian/Western), source of painting (Indian, Western), and art expertise (expert, nonexpert).*

*
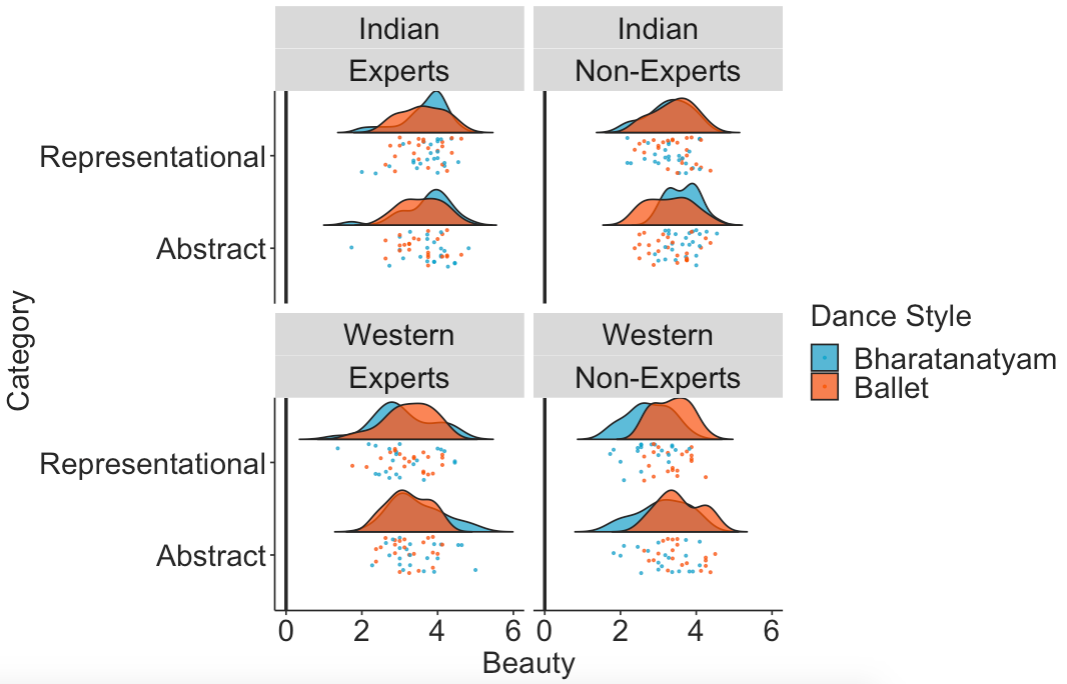
*

*Figure S17. Mean ratings of liking across category (abstract, representational), culture (Indian/Western), source of painting (Indian, Western), and art expertise (expert, nonexpert).*

*
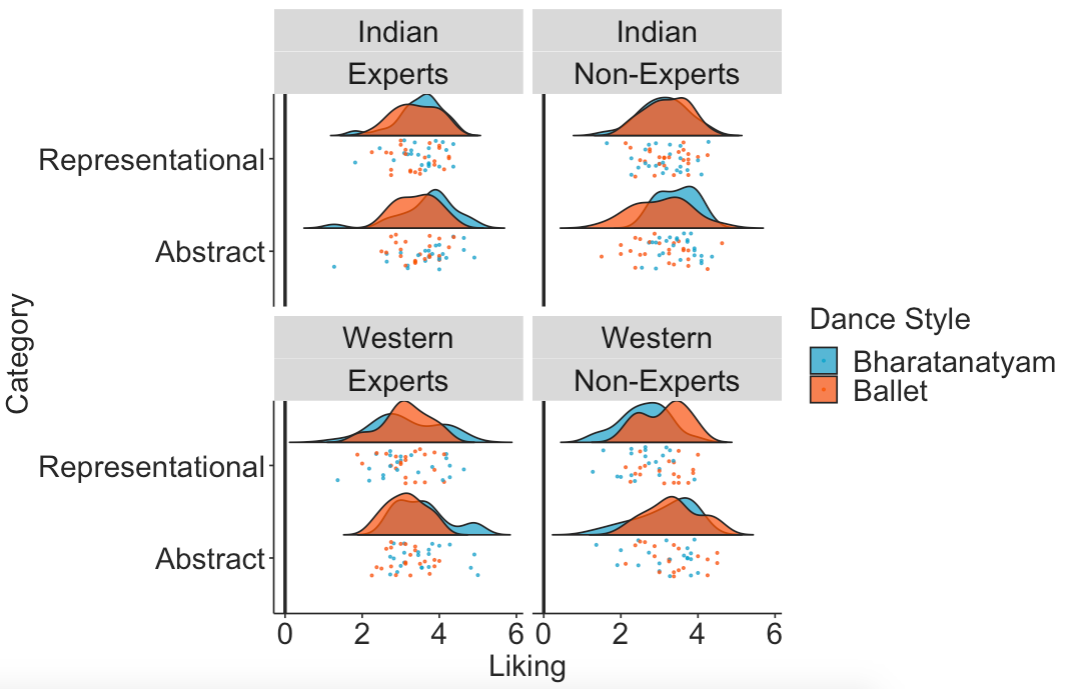
*

*Figure S18. Mean ratings of enjoyability across category (abstract, representational), culture (Indian/Western), source of painting (Indian, Western), and art expertise (expert, nonexpert).*

*
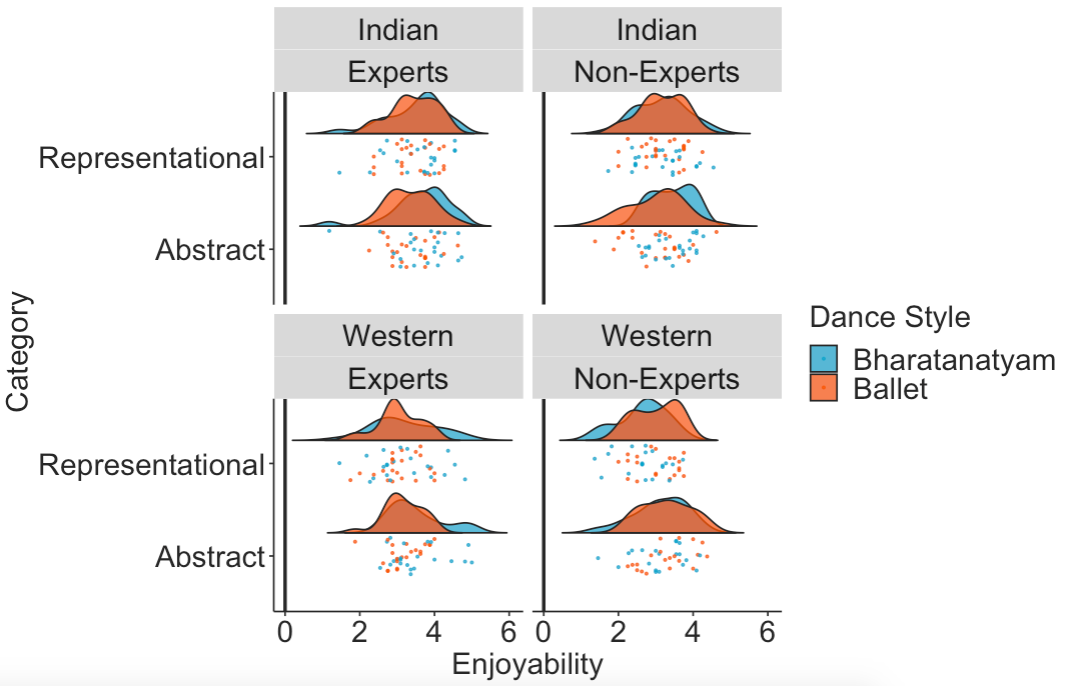
*

*Figure 19. The effect of dance style when categorized by participants (Bharatanatyam and ballet depicted in red and blue respectively), expertise (art experts or non-experts) and culture (Indian participants or Western participants) on the ratings of beauty (A), liking (B), and enjoyability (C).*

*
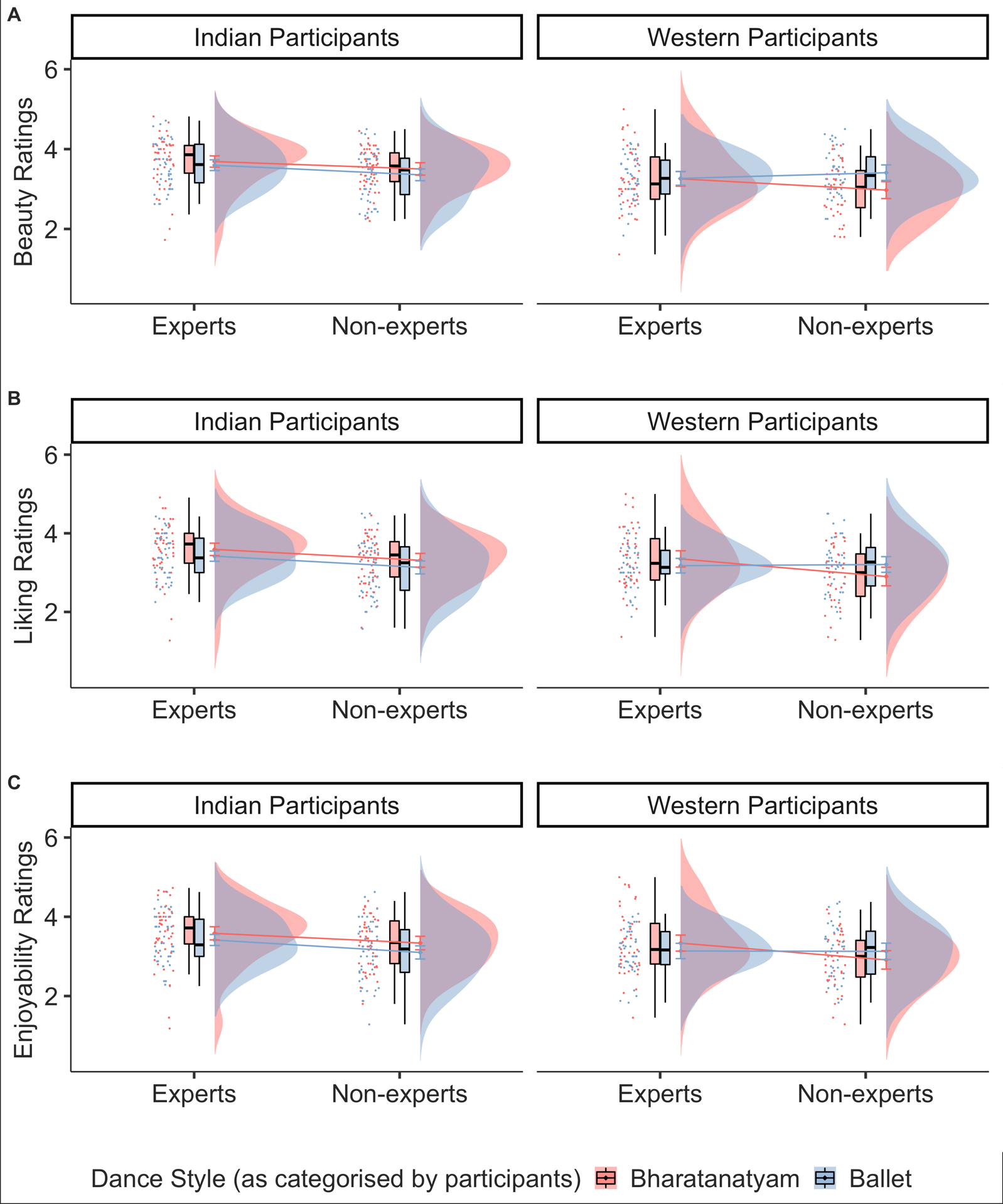
*

*Figure S20. RQ2: The effect of Expertise, Culture, and Dance Style when dance style is categorized by the participants themselves. For the outcome variables of beauty (A), liking (B), and enjoyability (C) beta estimates for the main model (in aqua blue) and the model with subjective variables (in purple) are plotted for each predictor variable along with their corresponding uncertainties (95% confidence interval width for a normal distribution for each estimate). Distributions are rescaled to match the height of each distribution.*

*
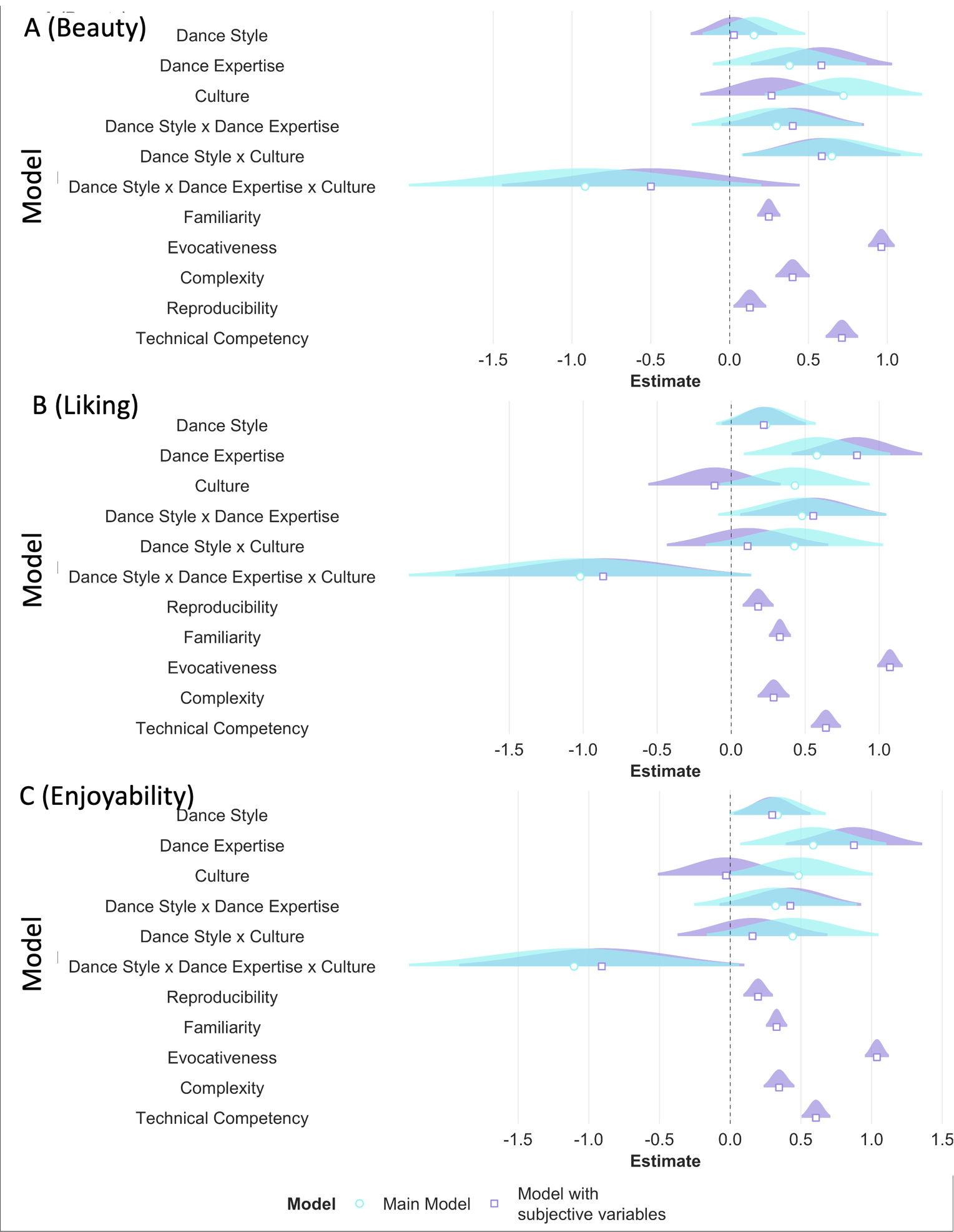
*

*Figure S21. For the outcome variables of beauty (A), liking (B), and enjoyability (C), beta estimates for the model with dance style as categorized by the experimenter (in pink) and the model with dance style as categorized by the participant (in green) are plotted for each predictor variable along with their corresponding uncertainties (95% confidence interval width for a normal distribution for each estimate). Distributions are rescaled to match the height of each distribution.*

*
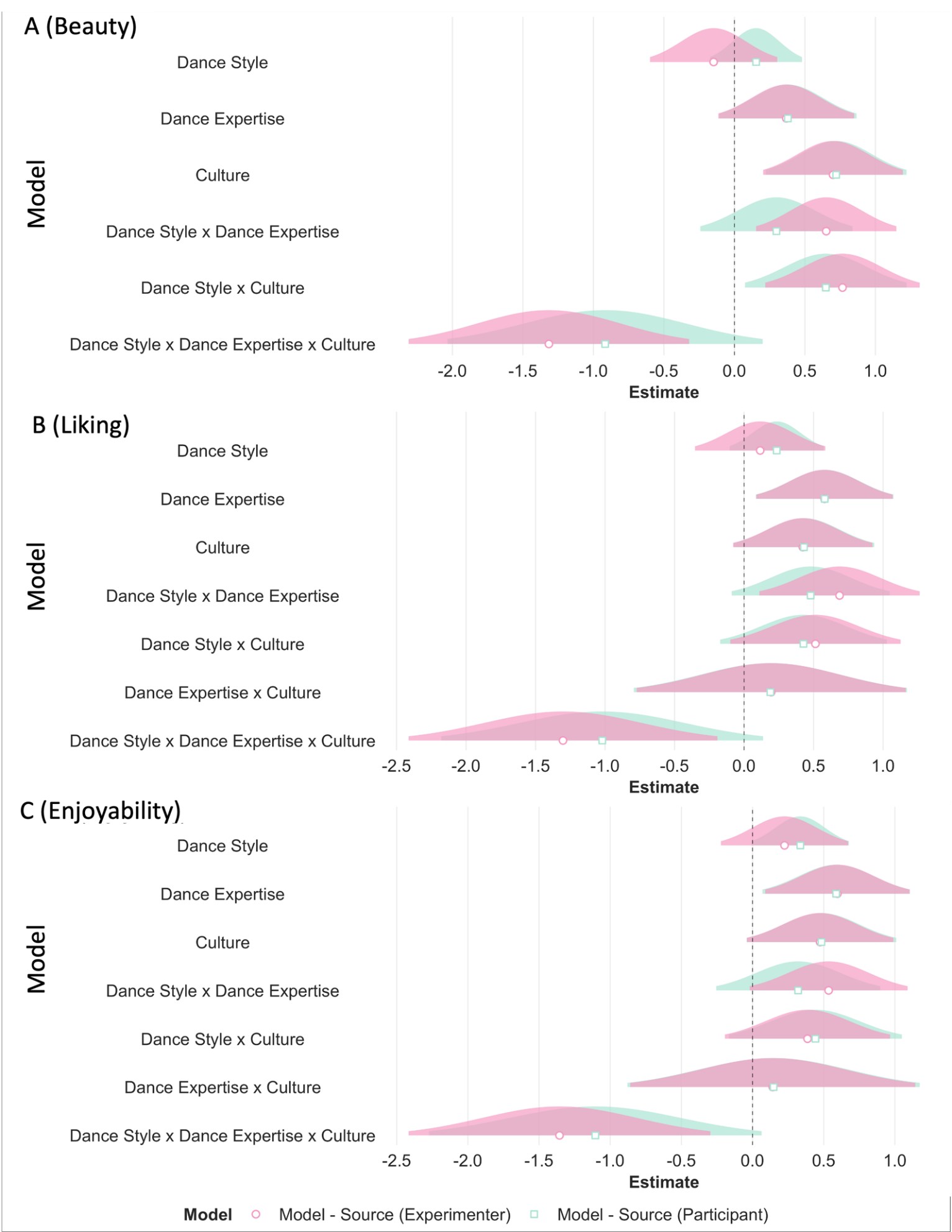
*

*Table S12. RQ1: Cumulative link mixed model: category*expertise*culture interaction – Experiment 2 – Dance.*

|  | *Main Model - Beauty* | | | *Main Model - Liking* | | | *Main Model - Enjoyability* | | | *Model with Subjective Variables - Beauty* | | | *Model with Subjective Variables - Liking* | | | *Model with Subjective Variables - Enjoyability* | | |
| --- | --- | --- | --- | --- | --- | --- | --- | --- | --- | --- | --- | --- | --- | --- | --- | --- | --- | --- |
| *Predictors* | *Log-Odds* | *CI* | *p* | *Log-Odds* | *CI* | *p* | *Log-Odds* | *CI* | *p* | *Log-Odds* | *CI* | *p* | *Log-Odds* | *CI* | *p* | *Log-Odds* | *CI* | *p* |
| 1\|2 | -4.25 | -4.61 – -3.89 | **<0.001** | -3.56 | -3.89 – -3.23 | **<0.001** | -3.53 | -3.87 – -3.19 | **<0.001** | 1.72 | 1.25 – 2.18 | **<0.001** | 2.56 | 2.11 – 3.00 | **<0.001** | 2.61 | 2.16 – 3.06 | **<0.001** |
| 2\|3 | -1.93 | -2.24 – -1.62 | **<0.001** | -1.59 | -1.89 – -1.29 | **<0.001** | -1.59 | -1.91 – -1.28 | **<0.001** | 4.69 | 4.22 – 5.15 | **<0.001** | 5.15 | 4.70 – 5.61 | **<0.001** | 5.14 | 4.67 – 5.60 | **<0.001** |
| 3\|4 | 0.11 | -0.20 – 0.41 | 0.499 | 0.34 | 0.05 – 0.64 | **0.023** | 0.40 | 0.10 – 0.71 | **0.010** | 7.37 | 6.86 – 7.88 | **<0.001** | 7.72 | 7.22 – 8.22 | **<0.001** | 7.76 | 7.25 – 8.26 | **<0.001** |
| 4\|5 | 2.71 | 2.39 – 3.03 | **<0.001** | 2.78 | 2.46 – 3.09 | **<0.001** | 2.72 | 2.40 – 3.04 | **<0.001** | 10.70 | 10.12 – 11.28 | **<0.001** | 10.85 | 10.28 – 11.42 | **<0.001** | 10.73 | 10.16 – 11.30 | **<0.001** |
| Category | -0.41 | -0.84 – 0.02 | 0.060 | -0.51 | -0.90 – -0.12 | **0.011** | -0.43 | -0.84 – -0.03 | **0.035** | -0.15 | -0.46 – 0.16 | 0.342 | -0.40 | -0.66 – -0.14 | **0.002** | -0.26 | -0.51 – -0.01 | **0.040** |
| Art Expertise | 0.42 | -0.07 – 0.92 | 0.094 | 0.62 | 0.13 – 1.12 | **0.013** | 0.63 | 0.12 – 1.15 | **0.015** | 0.58 | 0.12 – 1.04 | **0.013** | 0.87 | 0.43 – 1.32 | **<0.001** | 0.90 | 0.42 – 1.38 | **<0.001** |
| Culture | 0.75 | 0.24 – 1.27 | **0.004** | 0.46 | -0.04 – 0.96 | 0.070 | 0.50 | -0.01 – 1.02 | 0.057 | 0.27 | -0.20 – 0.74 | 0.260 | -0.09 | -0.54 – 0.36 | 0.689 | -0.02 | -0.49 – 0.46 | 0.950 |
| Category by Art Expertise | 0.27 | -0.23 – 0.77 | 0.296 | 0.20 | -0.27 – 0.68 | 0.401 | 0.21 | -0.28 – 0.70 | 0.400 | 0.04 | -0.41 – 0.50 | 0.850 | -0.01 | -0.46 – 0.44 | 0.971 | -0.04 | -0.48 – 0.39 | 0.841 |
| Category by Culture | 0.36 | -0.22 – 0.95 | 0.219 | 0.42 | -0.08 – 0.92 | 0.098 | 0.51 | 0.01 – 1.01 | **0.046** | -0.15 | -0.65 – 0.34 | 0.546 | -0.15 | -0.60 – 0.29 | 0.505 | -0.02 | -0.44 – 0.41 | 0.942 |
| Art Expertise by Culture | 0.31 | -0.69 – 1.30 | 0.548 | 0.10 | -0.88 – 1.07 | 0.848 | 0.06 | -0.96 – 1.09 | 0.903 | 0.24 | -0.67 – 1.15 | 0.605 | -0.10 | -0.97 – 0.78 | 0.827 | -0.14 | -1.10 – 0.81 | 0.767 |
| Category by Art Expertise by Culture | 0.11 | -0.93 – 1.14 | 0.838 | -0.33 | -1.25 – 0.60 | 0.491 | -0.23 | -1.20 – 0.75 | 0.650 | 0.19 | -0.71 – 1.10 | 0.676 | -0.18 | -1.02 – 0.66 | 0.671 | -0.11 | -0.96 – 0.75 | 0.809 |
| Familiarity |  |  |  |  |  |  |  |  |  | 0.28 | 0.22 – 0.35 | **<0.001** | 0.30 | 0.24 – 0.37 | **<0.001** | 0.30 | 0.23 – 0.37 | **<0.001** |
| Evocativeness |  |  |  |  |  |  |  |  |  | 0.98 | 0.89 – 1.06 | **<0.001** | 1.12 | 1.03 – 1.21 | **<0.001** | 1.07 | 0.98 – 1.16 | **<0.001** |
| Complexity |  |  |  |  |  |  |  |  |  | 0.39 | 0.28 – 0.50 | **<0.001** | 0.28 | 0.17 – 0.38 | **<0.001** | 0.34 | 0.23 – 0.44 | **<0.001** |
| Technical Competency |  |  |  |  |  |  |  |  |  | 0.74 | 0.64 – 0.85 | **<0.001** | 0.67 | 0.56 – 0.77 | **<0.001** | 0.65 | 0.55 – 0.75 | **<0.001** |
| Reproducibility |  |  |  |  |  |  |  |  |  | 0.11 | 0.01 – 0.21 | **0.031** | 0.16 | 0.06 – 0.26 | **0.002** | 0.16 | 0.06 – 0.26 | **0.002** |
| ICC | 0.37 | | | 0.35 | | | 0.37 | | | 0.30 | | | 0.27 | | | 0.30 | | |
| N | 90 _sid_ | | | 90 _sid_ | | | 90 _sid_ | | | 90 _sid_ | | | 90 _sid_ | | | 90 _sid_ | | |
|  | 38 _itemno_ | | | 38 _itemno_ | | | 38 _itemno_ | | | 38 _itemno_ | | | 38 _itemno_ | | | 38 _itemno_ | | |
| Observations | 3420 | | | 3420 | | | 3420 | | | 3420 | | | 3420 | | | 3420 | | |
| Marginal R^2^ / Conditional R^2^ | 0.044 / 0.397 | | | 0.042 / 0.377 | | | 0.040 / 0.396 | | | 0.462 / 0.622 | | | 0.479 / 0.620 | | | 0.464 / 0.624 | | |
| AIC | 8469.903 | | | 8862.563 | | | 8889.050 | | | 7001.706 | | | 7307.131 | | | 7366.135 | | |

*Table S13. RQ2: Cumulative link mixed model: source of painting*expertise*culture interaction- Experiment 2 - Dance.*

|  | *Main Model - Beauty* | | | *Main Model - Liking* | | | *Main Model - Enjoyability* | | | *Model with Subjective Variables - Beauty* | | | *Model with Subjective Variables - Liking* | | | *Model with Subjective Variables - Enjoyability* | | |
| --- | --- | --- | --- | --- | --- | --- | --- | --- | --- | --- | --- | --- | --- | --- | --- | --- | --- | --- |
| ***Predictors*** | *Log-Odds* | *CI* | *p* | *Log-Odds* | *CI* | *p* | *Log-Odds* | *CI* | *p* | *Log-Odds* | *CI* | *p* | *Log-Odds* | *CI* | *p* | *Log-Odds* | *CI* | *p* |
| **1\|2** | -4.25 | -4.61 – -3.88 | **<0.001** | -3.60 | -3.95 – -3.26 | **<0.001** | -3.53 | -3.87 – -3.18 | **<0.001** | 1.59 | 1.12 – 2.05 | **<0.001** | 2.46 | 2.01 – 2.92 | **<0.001** | 2.62 | 2.17 – 3.08 | **<0.001** |
| **2\|3** | -1.95 | -2.26 – -1.63 | **<0.001** | -1.63 | -1.94 – -1.31 | **<0.001** | -1.60 | -1.91 – -1.28 | **<0.001** | 4.54 | 4.07 – 5.00 | **<0.001** | 5.07 | 4.60 – 5.53 | **<0.001** | 5.15 | 4.68 – 5.62 | **<0.001** |
| **3\|4** | 0.09 | -0.22 – 0.40 | 0.574 | 0.34 | 0.03 – 0.65 | **0.033** | 0.41 | 0.09 – 0.72 | **0.011** | 7.22 | 6.71 – 7.72 | **<0.001** | 7.66 | 7.16 – 8.17 | **<0.001** | 7.79 | 7.28 – 8.30 | **<0.001** |
| **4\|5** | 2.72 | 2.39 – 3.04 | **<0.001** | 2.87 | 2.54 – 3.20 | **<0.001** | 2.79 | 2.46 – 3.12 | **<0.001** | 10.57 | 10.00 – 11.14 | **<0.001** | 10.88 | 10.31 – 11.45 | **<0.001** | 10.85 | 10.27 – 11.42 | **<0.001** |
| **Dance Style** | -0.15 | -0.60 – 0.30 | 0.522 | 0.12 | -0.35 – 0.58 | 0.628 | 0.22 | -0.22 – 0.67 | 0.325 | -0.29 | -0.58 – 0.00 | 0.051 | 0.05 | -0.27 – 0.37 | 0.766 | 0.20 | -0.08 – 0.48 | 0.156 |
| **Culture** | 0.37 | -0.11 – 0.85 | 0.134 | 0.58 | 0.09 – 1.07 | **0.021** | 0.60 | 0.09 – 1.10 | **0.021** | 0.56 | 0.11 – 1.00 | **0.014** | 0.83 | 0.39 – 1.27 | **<0.001** | 0.88 | 0.40 – 1.36 | **<0.001** |
| **Art Expertise** | 0.70 | 0.20 – 1.19 | **0.006** | 0.42 | -0.08 – 0.92 | 0.099 | 0.47 | -0.04 – 0.99 | 0.071 | 0.24 | -0.21 – 0.70 | 0.291 | -0.12 | -0.56 – 0.33 | 0.606 | -0.04 | -0.52 – 0.44 | 0.879 |
| **Dance Style by Culture** | 0.65 | 0.15 – 1.15 | **0.010** | 0.69 | 0.11 – 1.26 | **0.020** | 0.53 | -0.02 – 1.09 | 0.059 | 0.74 | 0.32 – 1.15 | **0.001** | 0.77 | 0.28 – 1.26 | **0.002** | 0.61 | 0.12 – 1.10 | **0.015** |
| **Dance Style by Art Expertise** | 0.77 | 0.22 – 1.31 | **0.006** | 0.51 | -0.10 – 1.13 | 0.100 | 0.39 | -0.19 – 0.97 | 0.192 | 0.82 | 0.33 – 1.31 | **0.001** | 0.27 | -0.28 – 0.83 | 0.338 | 0.13 | -0.41 – 0.66 | 0.638 |
| **Art Expertise by Culture** | 0.41 | -0.55 – 1.37 | 0.402 | 0.20 | -0.77 – 1.16 | 0.693 | 0.14 | -0.86 – 1.14 | 0.781 | 0.32 | -0.57 – 1.22 | 0.481 | -0.03 | -0.89 – 0.84 | 0.951 | -0.10 | -1.05 – 0.85 | 0.839 |
| **Dance Style by Art Expertise by Culture** | -1.32 | -2.31 – -0.32 | **0.010** | -1.30 | -2.41 – -0.19 | **0.021** | -1.36 | -2.42 – -0.30 | **0.012** | -0.84 | -1.72 – 0.05 | 0.064 | -1.00 | -1.98 – -0.01 | **0.048** | -0.98 | -1.97 – 0.02 | 0.054 |
| **Familiarity** |  |  |  |  |  |  |  |  |  | 0.25 | 0.17 – 0.32 | **<0.001** | 0.32 | 0.25 – 0.40 | **<0.001** | 0.33 | 0.26 – 0.41 | **<0.001** |
| **Evocativeness** |  |  |  |  |  |  |  |  |  | 0.96 | 0.88 – 1.05 | **<0.001** | 1.07 | 0.99 – 1.16 | **<0.001** | 1.04 | 0.96 – 1.13 | **<0.001** |
| **Complexity** |  |  |  |  |  |  |  |  |  | 0.40 | 0.30 – 0.51 | **<0.001** | 0.29 | 0.18 – 0.40 | **<0.001** | 0.35 | 0.24 – 0.46 | **<0.001** |
| **Technical Competency** |  |  |  |  |  |  |  |  |  | 0.72 | 0.61 – 0.82 | **<0.001** | 0.64 | 0.53 – 0.74 | **<0.001** | 0.60 | 0.50 – 0.70 | **<0.001** |
| **Reproducibility** |  |  |  |  |  |  |  |  |  | 0.13 | 0.03 – 0.24 | **0.013** | 0.19 | 0.08 – 0.29 | **<0.001** | 0.21 | 0.10 – 0.31 | **<0.001** |
| ICC | 0.37 | | | 0.38 | | | 0.39 | | | 0.29 | | | 0.30 | | | 0.32 | | |
| N | 90 _sid_ | | | 90 _sid_ | | | 90 _sid_ | | | 90 _sid_ | | | 90 _sid_ | | | 90 _sid_ | | |
|  | 38 _itemno_ | | | 38 _itemno_ | | | 38 _itemno_ | | | 38 _itemno_ | | | 38 _itemno_ | | | 38 _itemno_ | | |
| Observations | 3420 | | | 3420 | | | 3420 | | | 3420 | | | 3420 | | | 3420 | | |
| Marginal R^2^ / Conditional R^2^ | 0.050 / 0.398 | | | 0.039 / 0.408 | | | 0.040 / 0.413 | | | 0.467 / 0.620 | | | 0.465 / 0.623 | | | 0.457 / 0.631 | | |
| AIC | 8436.326 | | | 8743.253 | | | 8814.701 | | | 6977.282 | | | 7241.428 | | | 7307.679 | | |

*Table S14. RQ2: Cumulative link mixed model: source of painting*expertise*culture interaction, when source of painting is categorized by the participants themselves - Experiment 2 - Dance.*

|  | *Main Model - Beauty* | | | *Main Model - Liking* | | | *Main Model - enjoyability* | | | *Model with Subjective Variables - Beauty* | | | *Model with Subjective Variables - Liking* | | | *Model with Subjective Variables - enjoyability* | | |
| --- | --- | --- | --- | --- | --- | --- | --- | --- | --- | --- | --- | --- | --- | --- | --- | --- | --- | --- |
| ***Predictors*** | *Log-Odds* | *CI* | *p* | *Log-Odds* | *CI* | *p* | *Log-Odds* | *CI* | *p* | *Log-Odds* | *CI* | *p* | *Log-Odds* | *CI* | *p* | *Log-Odds* | *CI* | *p* |
| **1\|2** | -4.25 | -4.62 – -3.88 | **<0.001** | -3.60 | -3.95 – -3.25 | **<0.001** | -3.55 | -3.91 – -3.20 | **<0.001** | 1.59 | 1.12 – 2.05 | **<0.001** | 2.46 | 2.01 – 2.92 | **<0.001** | 2.62 | 2.17 – 3.08 | **<0.001** |
| **2\|3** | -1.94 | -2.26 – -1.62 | **<0.001** | -1.63 | -1.94 – -1.31 | **<0.001** | -1.61 | -1.93 – -1.29 | **<0.001** | 4.54 | 4.07 – 5.00 | **<0.001** | 5.07 | 4.60 – 5.53 | **<0.001** | 5.15 | 4.68 – 5.62 | **<0.001** |
| **3\|4** | 0.11 | -0.20 – 0.42 | 0.491 | 0.34 | 0.03 – 0.65 | **0.033** | 0.41 | 0.09 – 0.73 | **0.011** | 7.22 | 6.71 – 7.72 | **<0.001** | 7.66 | 7.16 – 8.17 | **<0.001** | 7.79 | 7.28 – 8.30 | **<0.001** |
| **4\|5** | 2.76 | 2.43 – 3.09 | **<0.001** | 2.88 | 2.54 – 3.21 | **<0.001** | 2.82 | 2.48 – 3.15 | **<0.001** | 10.57 | 10.00 – 11.14 | **<0.001** | 10.88 | 10.31 – 11.45 | **<0.001** | 10.85 | 10.27 – 11.42 | **<0.001** |
| **Dance Style** | 0.15 | -0.17 – 0.48 | 0.354 | 0.23 | -0.10 – 0.57 | 0.173 | 0.34 | -0.01 – 0.68 | 0.054 | -0.29 | -0.58 – 0.00 | 0.051 | 0.05 | -0.27 – 0.37 | 0.766 | 0.20 | -0.08 – 0.48 | 0.156 |
| **Culture** | 0.38 | -0.11 – 0.87 | 0.127 | 0.58 | 0.08 – 1.07 | **0.022** | 0.59 | 0.07 – 1.10 | **0.026** | 0.56 | 0.11 – 1.00 | **0.014** | 0.83 | 0.39 – 1.27 | **<0.001** | 0.88 | 0.40 – 1.36 | **<0.001** |
| **Art Expertise** | 0.72 | 0.22 – 1.22 | **0.005** | 0.43 | -0.07 – 0.94 | 0.095 | 0.48 | -0.04 – 1.01 | 0.071 | 0.24 | -0.21 – 0.70 | 0.291 | -0.12 | -0.56 – 0.33 | 0.606 | -0.04 | -0.52 – 0.44 | 0.879 |
| **Dance Style by Culture** | 0.30 | -0.24 – 0.84 | 0.279 | 0.48 | -0.09 – 1.05 | 0.098 | 0.32 | -0.26 – 0.90 | 0.276 | 0.74 | 0.32 – 1.15 | **0.001** | 0.77 | 0.28 – 1.26 | **0.002** | 0.61 | 0.12 – 1.10 | **0.015** |
| **Dance Style by Art Expertise** | 0.65 | 0.08 – 1.22 | **0.027** | 0.43 | -0.17 – 1.03 | 0.162 | 0.44 | -0.17 – 1.05 | 0.154 | 0.82 | 0.33 – 1.31 | **0.001** | 0.27 | -0.28 – 0.83 | 0.338 | 0.13 | -0.41 – 0.66 | 0.638 |
| **Art Expertise by Culture** | 0.38 | -0.60 – 1.36 | 0.445 | 0.19 | -0.79 – 1.17 | 0.706 | 0.15 | -0.88 – 1.17 | 0.778 | 0.32 | -0.57 – 1.22 | 0.481 | -0.03 | -0.89 – 0.84 | 0.951 | -0.10 | -1.05 – 0.85 | 0.839 |
| **Dance Style by Art Expertise by Culture** | -0.92 | -2.03 – 0.20 | 0.108 | -1.02 | -2.18 – 0.14 | 0.084 | -1.10 | -2.27 – 0.06 | 0.064 | -0.84 | -1.72 – 0.05 | 0.064 | -1.00 | -1.98 – -0.01 | **0.048** | -0.98 | -1.97 – 0.02 | 0.054 |
| **Familiarity** |  |  |  |  |  |  |  |  |  | 0.25 | 0.17 – 0.32 | **<0.001** | 0.32 | 0.25 – 0.40 | **<0.001** | 0.33 | 0.26 – 0.41 | **<0.001** |
| **Evocativeness** |  |  |  |  |  |  |  |  |  | 0.96 | 0.88 – 1.05 | **<0.001** | 1.07 | 0.99 – 1.16 | **<0.001** | 1.04 | 0.96 – 1.13 | **<0.001** |
| **Complexity** |  |  |  |  |  |  |  |  |  | 0.40 | 0.30 – 0.51 | **<0.001** | 0.29 | 0.18 – 0.40 | **<0.001** | 0.35 | 0.24 – 0.46 | **<0.001** |
| **Technical Competency** |  |  |  |  |  |  |  |  |  | 0.72 | 0.61 – 0.82 | **<0.001** | 0.64 | 0.53 – 0.74 | **<0.001** | 0.60 | 0.50 – 0.70 | **<0.001** |
| **Reproducibility** |  |  |  |  |  |  |  |  |  | 0.13 | 0.03 – 0.24 | **0.013** | 0.19 | 0.08 – 0.29 | **<0.001** | 0.21 | 0.10 – 0.31 | **<0.001** |
| ICC | 0.38 | | | 0.39 | | | 0.40 | | | 0.29 | | | 0.30 | | | 0.32 | | |
| N | 90 _sid_ | | | 90 _sid_ | | | 90 _sid_ | | | 90 _sid_ | | | 90 _sid_ | | | 90 _sid_ | | |
|  | 38 _itemno_ | | | 38 _itemno_ | | | 38 _itemno_ | | | 38 _itemno_ | | | 38 _itemno_ | | | 38 _itemno_ | | |
| Observations | 3420 | | | 3420 | | | 3420 | | | 3420 | | | 3420 | | | 3420 | | |
| Marginal R^2^ / Conditional R^2^ | 0.044 / 0.403 | | | 0.036 / 0.408 | | | 0.039 / 0.425 | | | 0.467 / 0.620 | | | 0.465 / 0.623 | | | 0.457 / 0.631 | | |
| AIC | 8420.739 | | | 8750.097 | | | 8790.285 | | | 6977.282 | | | 7241.428 | | | 7307.679 | | |

*Table S15. RQ1. Cumulative link model statistics for category*art expertise for Indian and Western participants separately.*

|  | *Main Model - Beauty (Indian)* | | | *Main Model - Beauty (Western)* | | | *Main Model - Liking (Indian)* | | | *Main Model - Liking (Western)* | | | *Main Model - Enjoyability (Indian)* | | | *Main Model - Enjoyability (Western)* | | |
| --- | --- | --- | --- | --- | --- | --- | --- | --- | --- | --- | --- | --- | --- | --- | --- | --- | --- | --- |
| ***Predictors*** | *Log-Odds* | *CI* | *p* | *Log-Odds* | *CI* | *p* | *Log-Odds* | *CI* | *p* | *Log-Odds* | *CI* | *p* | *Log-Odds* | *CI* | *p* | *Log-Odds* | *CI* | *p* |
| **1\|2** | -4.58 | -5.08 – -4.08 | **<0.001** | -3.90 | -4.38 – -3.41 | **<0.001** | -3.74 | -4.18 – -3.31 | **<0.001** | -3.37 | -3.82 – -2.91 | **<0.001** | -3.80 | -4.27 – -3.33 | **<0.001** | -3.26 | -3.70 – -2.82 | **<0.001** |
| **2\|3** | -2.35 | -2.75 – -1.94 | **<0.001** | -1.51 | -1.93 – -1.09 | **<0.001** | -1.86 | -2.25 – -1.48 | **<0.001** | -1.31 | -1.72 – -0.90 | **<0.001** | -1.82 | -2.24 – -1.40 | **<0.001** | -1.36 | -1.76 – -0.96 | **<0.001** |
| **3\|4** | -0.33 | -0.72 – 0.06 | 0.095 | 0.54 | 0.13 – 0.96 | **0.011** | 0.08 | -0.29 – 0.46 | 0.654 | 0.61 | 0.20 – 1.01 | **0.003** | 0.13 | -0.28 – 0.54 | 0.538 | 0.69 | 0.29 – 1.08 | **0.001** |
| **4\|5** | 2.47 | 2.07 – 2.88 | **<0.001** | 2.87 | 2.42 – 3.31 | **<0.001** | 2.65 | 2.25 – 3.04 | **<0.001** | 2.86 | 2.43 – 3.30 | **<0.001** | 2.49 | 2.07 – 2.92 | **<0.001** | 2.93 | 2.50 – 3.36 | **<0.001** |
| **Category** | -0.23 | -0.68 – 0.23 | 0.337 | -0.59 | -1.16 – -0.01 | **0.045** | -0.30 | -0.73 – 0.13 | 0.169 | -0.71 | -1.21 – -0.21 | **0.005** | -0.18 | -0.64 – 0.29 | 0.463 | -0.69 | -1.18 – -0.21 | **0.005** |
| **Art Expertise** | 0.59 | -0.08 – 1.27 | 0.086 | 0.26 | -0.46 – 0.98 | 0.475 | 0.69 | 0.03 – 1.34 | **0.041** | 0.57 | -0.16 – 1.30 | 0.124 | 0.67 | -0.05 – 1.39 | 0.070 | 0.60 | -0.12 – 1.32 | 0.102 |
| **Category by Art Expertise** | 0.33 | -0.26 – 0.91 | 0.275 | 0.20 | -0.64 – 1.04 | 0.634 | 0.03 | -0.53 – 0.60 | 0.905 | 0.37 | -0.37 – 1.12 | 0.327 | 0.09 | -0.51 – 0.69 | 0.763 | 0.33 | -0.44 – 1.09 | 0.406 |
| ICC | 0.35 | | | 0.38 | | | 0.34 | | | 0.36 | | | 0.38 | | | 0.36 | | |
| N | 48 _sid_ | | | 42 _sid_ | | | 48 _sid_ | | | 42 _sid_ | | | 48 _sid_ | | | 42 _sid_ | | |
|  | 38 _itemno_ | | | 38 _itemno_ | | | 38 _itemno_ | | | 38 _itemno_ | | | 38 _itemno_ | | | 38 _itemno_ | | |
| Observations | 1824 | | | 1596 | | | 1824 | | | 1596 | | | 1824 | | | 1596 | | |
| Marginal R^2^ / Conditional R^2^ | 0.021 / 0.368 | | | 0.019 / 0.396 | | | 0.028 / 0.355 | | | 0.040 / 0.388 | | | 0.022 / 0.395 | | | 0.040 / 0.382 | | |
| AIC | 4368.038 | | | 4104.831 | | | 4648.615 | | | 4230.471 | | | 4700.388 | | | 4213.339 | | |

*Table S16. RQ1. Means and SDs of beauty, liking, enjoyability ratings for the category x art expertise x culture interaction.*

| Expertise | culture | category | mean_beauty | mean_liking | mean_enjoyability | sd_beauty | sd_liking | sd_enjoyability |
| --- | --- | --- | --- | --- | --- | --- | --- | --- |
| Experts | Indian | Abstract | 3.650 | 3.551 | 3.513 | 0.615 | 0.676 | 0.700 |
| Experts | Indian | Representational | 3.634 | 3.446 | 3.474 | 0.595 | 0.579 | 0.681 |
| Experts | Western | Abstract | 3.346 | 3.349 | 3.339 | 0.626 | 0.636 | 0.643 |
| Experts | Western | Representational | 3.170 | 3.153 | 3.125 | 0.716 | 0.734 | 0.732 |
| Non-Experts | Indian | Abstract | 3.468 | 3.253 | 3.230 | 0.540 | 0.667 | 0.688 |
| Non-Experts | Indian | Representational | 3.345 | 3.153 | 3.165 | 0.557 | 0.591 | 0.638 |
| Non-Experts | Western | Abstract | 3.339 | 3.248 | 3.201 | 0.660 | 0.728 | 0.682 |
| Non-Experts | Western | Representational | 3.052 | 2.866 | 2.835 | 0.606 | 0.661 | 0.637 |

D] Dance Expertise Questionnaire

The following questions were used to gauge the dance expertise of participants. The total expertise score was the sum total of scores for each individual item (min=0, max=20).

On average, you see a dance performance once every:

0 = Never

1 = Year

2 = 6 months

3 = 2 months

4 = Month

5 = Week

On average, you perform dance (on-stage/live) once every:

0 = Never

1 = Year

2 = 6 months

3 = 2 months

4 = Month

5 = Week

On average, you read about dance theory once every:

0 = Never

1 = Year

2 = 6 months

3 = 2 months

4 = Month

5 = Week

In the average week, how many hours do you spend dancing (as a hobby or practicing, **not performing**)

0 = Never

1 = Year

2 = 6 months

3 = 2 months

4 = Month

5 = Week

**Categorisation task accuracy.**

*Table S17. Accuracy on the Categorisation Task (categorise as Indian painting or Western painting) for Experiment 1 across category, art expertise, culture, and source of painting.*

| Expertise | Culture | Source of Painting | Category | Mean Accuracy | SD Accuracy |
| --- | --- | --- | --- | --- | --- |
| expert | indian | indian | abstract | 0.742 | 0.143 |
| expert | indian | indian | representational | 0.476 | 0.157 |
| expert | indian | western | abstract | 0.631 | 0.282 |
| expert | indian | western | representational | 0.871 | 0.212 |
| expert | western | indian | abstract | 0.623 | 0.181 |
| expert | western | indian | representational | 0.421 | 0.165 |
| expert | western | western | abstract | 0.577 | 0.235 |
| expert | western | western | representational | 0.845 | 0.157 |
| nonexpert | indian | indian | abstract | 0.621 | 0.144 |
| nonexpert | indian | indian | representational | 0.523 | 0.154 |
| nonexpert | indian | western | abstract | 0.697 | 0.229 |
| nonexpert | indian | western | representational | 0.614 | 0.204 |
| nonexpert | western | indian | abstract | 0.615 | 0.207 |
| nonexpert | western | indian | representational | 0.396 | 0.167 |
| nonexpert | western | western | abstract | 0.543 | 0.183 |
| nonexpert | western | western | representational | 0.846 | 0.151 |

*Table S18. Accuracy on the Categorisation Task (categorise as Bharatanatyam or Ballet) for Experiment 2 across category, dance expertise, culture, and dance style.*

| Expertise | Culture | Dance Style | Category | Mean Accuracy | SD Accuracy |
| --- | --- | --- | --- | --- | --- |
| expert | indian | bnat | abstract | 0.996 | 0.018 |
| expert | indian | bnat | representational | 0.940 | 0.093 |
| expert | indian | ballet | abstract | 1.000 | 0.000 |
| expert | indian | ballet | representational | 0.858 | 0.137 |
| expert | western | bnat | abstract | 0.987 | 0.031 |
| expert | western | bnat | representational | 0.921 | 0.149 |
| expert | western | ballet | abstract | 0.988 | 0.036 |
| expert | western | ballet | representational | 0.863 | 0.153 |
| nonexpert | indian | bnat | abstract | 0.989 | 0.030 |
| nonexpert | indian | bnat | representational | 0.814 | 0.245 |
| nonexpert | indian | ballet | abstract | 0.980 | 0.046 |
| nonexpert | indian | ballet | representational | 0.825 | 0.260 |
| nonexpert | western | bnat | abstract | 0.977 | 0.065 |
| nonexpert | western | bnat | representational | 0.800 | 0.160 |
| nonexpert | western | ballet | abstract | 0.950 | 0.085 |
| nonexpert | western | ballet | representational | 0.812 | 0.200 |

**Art expertise of all participants**

*Table S19. Distribution of art expertise across participants in the main experiment (Experiment 1).*

| Expertise | Culture | mean_score on the dance expertise questionnaire | sd_score on the dance expertise questionnaire |
| --- | --- | --- | --- |
| Expert | Indian | 31.190 | 9.646 |
| Expert | Western | 31.666 | 9.906 |
| Non-Expert | Indian | 15.333 | 6.938 |
| Non-Expert | Western | 12.807 | 4.664 |

*Table S20. Dance expertise across participants in the main experiment (Experiment 2).*

| Expertise | Culture | mean_score on the dance expertise questionnaire | sd_score on the dance expertise questionnaire | Mean Years of training | SD Years of training |
| --- | --- | --- | --- | --- | --- |
| Expert | Indian | 14.478 | 4.907 | 9.94 | 5.99 |
| Expert | Western | 14.409 | 5.474 | 14.48 | 12.77 |
| Non-Expert | Indian | 10.320 | 5.444 |  |  |
| Non-Expert | Western | 7.250 | 3.176 |  |  |

24 Indian participants and 21 Western participants received formal training in Bharatanatyam and Ballet. Out of 45 non-expert participants across both the cultures, 32 participants did not receive any formal training in any dance style, while 13 participants received limited training in other styles outside of ballet and Bharatanatyam. One participant trained in ballet also had limited training in Bharatanatyam (<1 year) and street dance (1 year). Three participants who were trained in Bharatanatyam had limited training in other dance styles.

Participants responded to the following question–

Artist Q – Please select the dance style in which you have received formal training

1 Bharatanatyam

2 Ballet

3 Both Ballet & Bharatanatyam

4 Ballet, Bharatanatyam, & Other Styles

5 Styles other than Ballet & Bharatanatyam

6 I have received no formal training in any dance style
